# Supplementary material for: Mitochondrial‐derived vesicles retain membrane potential and contain a functional ATP synthase
Source: EMBO Rep. 2023 Mar 17;24(5):e56114. doi: 10.15252/embr.202256114 (PMC10157309; doi:10.15252/embr.202256114)
Supplement: Supplementary file 1 — Expanded View Figures PDF [file EMBR-24-e56114-s005.pdf]

Expanded View Figures

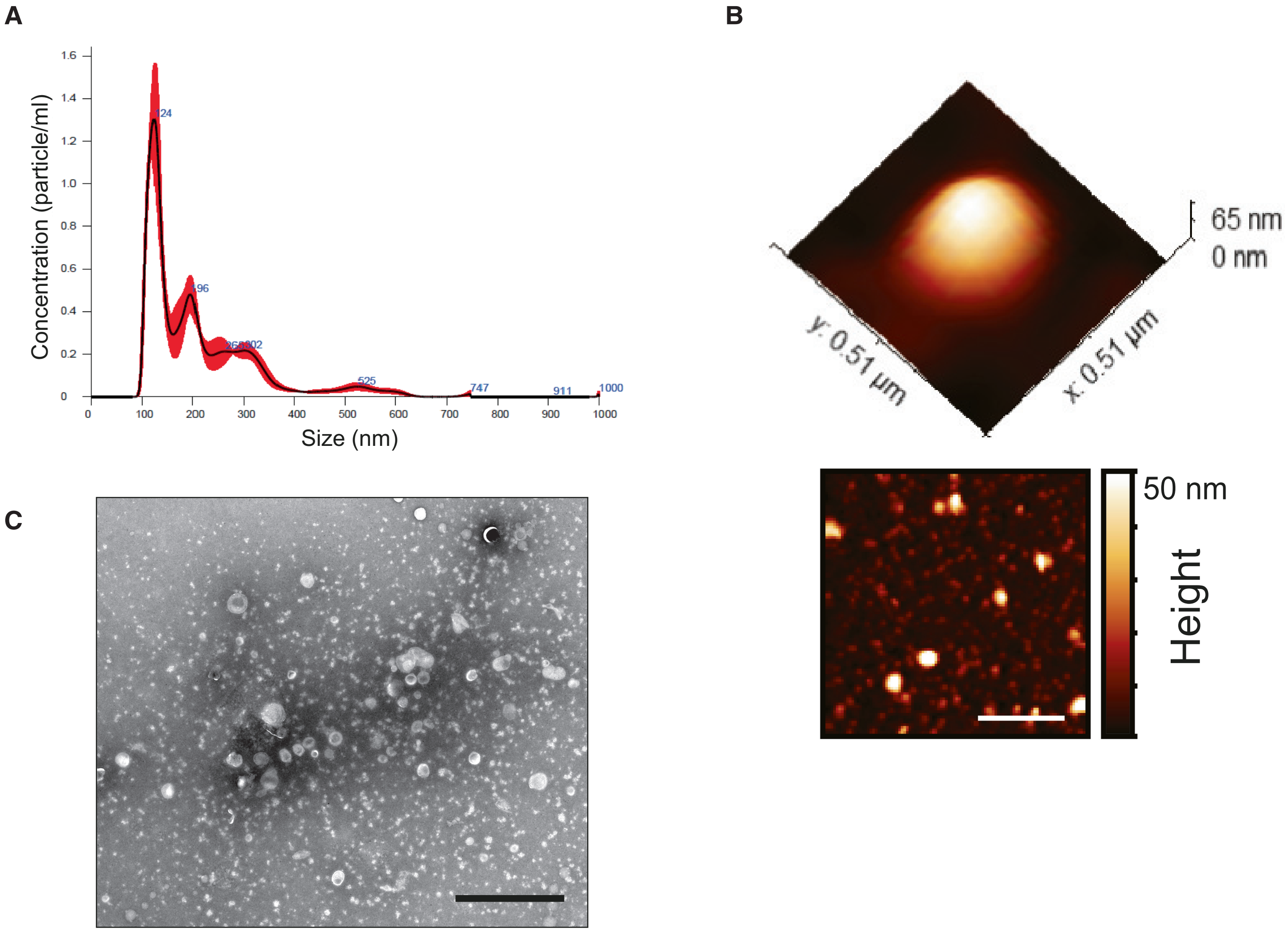

Figure EV1. Characterization of MDVs derived from Dnm1 KO mitochondria.

A Nanoparticle tracking analysis. Vesicle size distribution and concentration were performed using nanoparticle tracking analysis (NTA; Malvern Instruments, Nanosight NS300). Sample size distributions were calibrated in a liquid suspension by the analysis of Brownian motion via light scattering. Nanosight provides single particle size and concentration measurements.

B Atomic force microscopy. Representative AFM image and a 3D AFM image of one representative vesicle (Dnm1 KO), adsorbed on a mica modified with Mg^{2+} and imaged under PBS.

C TEM images of MDVs. Samples were stained with 2.0% uranyl acetate or 2.0% phosphotungstic acid. 5 µl of vesicles was placed on Formvar/carbon-coated copper 200 mesh grids (EMS), mixed with 5 µl of PTA for 10–20 s, while excess stain was blotted off and grids were dried. Samples were examined with Jeol (Jem-1400 Plus) transmission electron microscope. Scale bar—2 µm.

Source data are available online for this figure.

© 2023 Weizmann Institute of Science

Figure EV2. Vesicle characterization.

A Vesicle concentrations according to nanoparticle tracking analysis over time. Vesicle concentration was measured at the indicated time points, n = two repeats.
B Mitochondria ATP levels with time. Isolated mitochondria from wild-type cells were incubated for 0, 4 h, and 24 h at 30°C prior to measuring ATP production, in the absence or presence of succinate, by luciferin-luciferase luminometry as described in the methods section. Each value represents the mean ± SD for n = 3 technical repeats. Significant differences were detected using t-test, P-value = 1.9558E-05/1.03993E-06 for 24 and 4 h mitochondria with succinate, respectively.
C Western blot analysis of isolated MDVs. MDVs were purified from wild-type or KO-isolated mitochondria as mentioned, and the presence of Mdh1p and Por1p was assessed by western blot analysis using Mdh1 antisera.
D Vesicles count in WT and ΔDnm1 strains. MDVs isolated from Wild-type and Dnm1 KO strains were analyzed using nanoparticle tracking analysis (NTA; Malvern Instruments, Nanosight NS300). n = two repeats.

Source data are available online for this figure.

© 2023 Weizmann Institute of Science

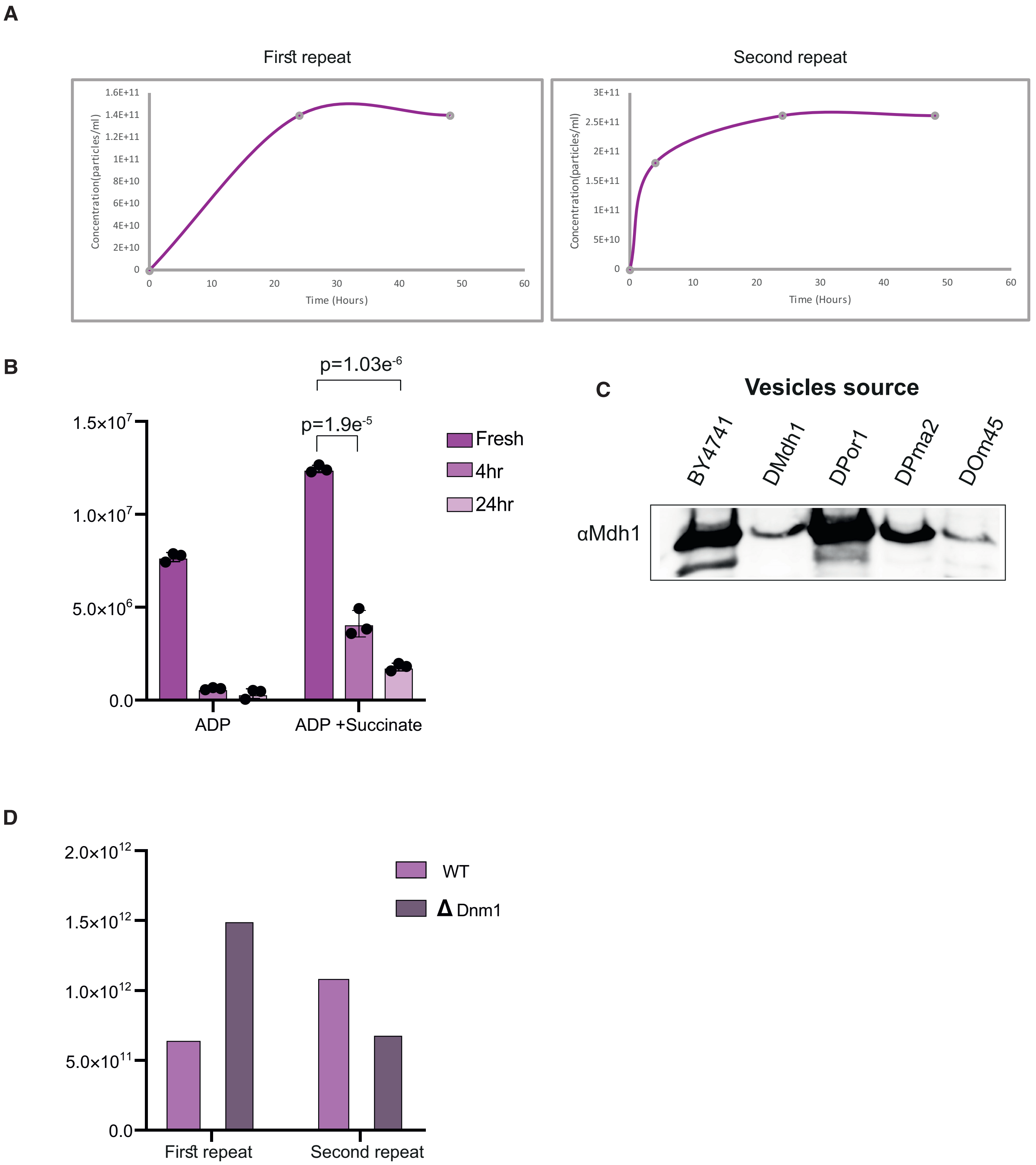
A
First repeat
Second repeat
Concentration(particles/ml)
Time (Hours)
B
p=1.03e-6
p=1.9e-5
Fresh
4hr
24hr
ADP
ADP +Succinate
C
Vesicles source
BY4741
DMdh1
DPor1
DPma2
DOm45
αMdh1
D
WT
Δ Dnm1
First repeat
Second repeat

Figure EV2.

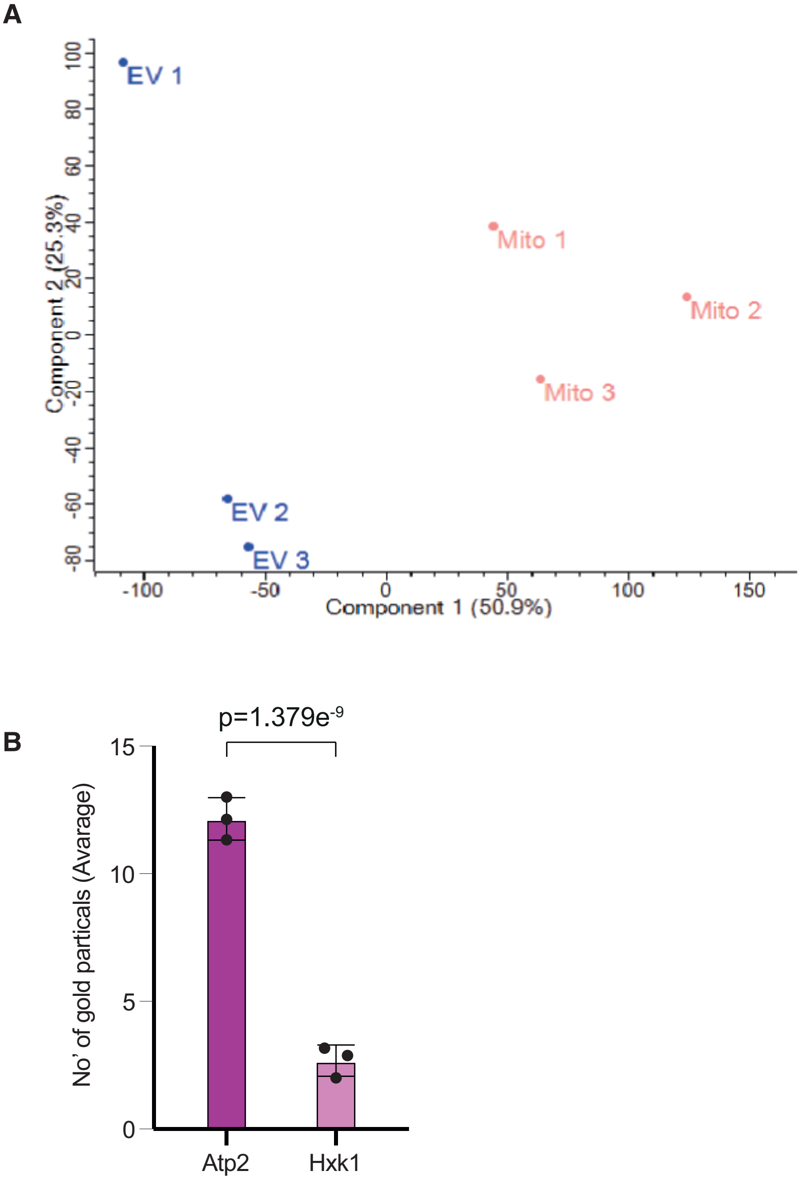

Figure EV3. Data for MDVs from yeast wild-type mitochondria.

A Principal component analysis (PCA) visualizes the projection of the dataset defined by PCA in 2-dimensional viewers, transforming the large set of protein information into a protein profile of each sample. The analysis was done by Perseus software.

B The average number of gold particles of Atp2 is significantly higher compared with the controls. The number of gold particles was measured using ImageJ software (ImageJ.nih.gov). Each value represents the mean ± SD for $n = 3$ biological repeats. Significant differences were detected using *t*-test, *P*-value = 1.379E-09.

Source data are available online for this figure.

 © 2023 Weizmann Institute of Science

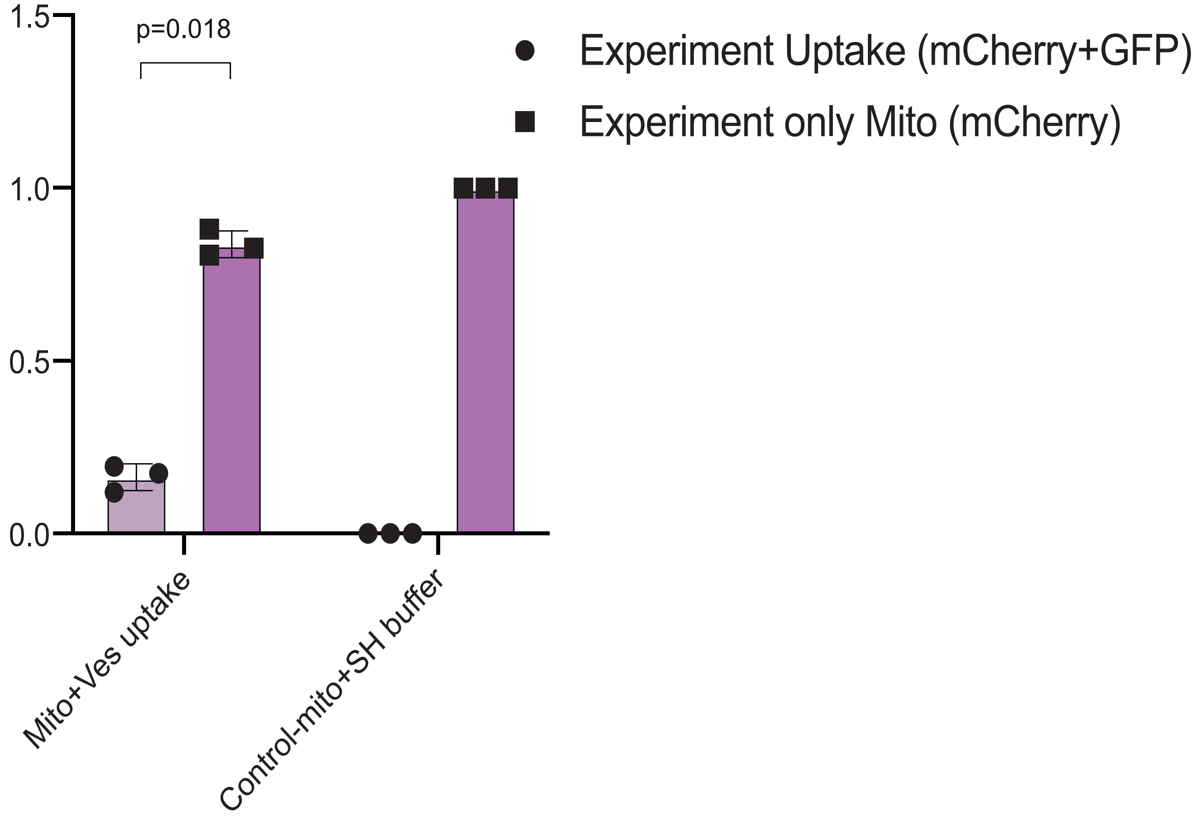

Figure EV4. Co-localization of mitochondria and MDVs as visualized by imaging flow cytometry.

mCherry-labeled mitochondria were preincubated in the presence or absence of GFP-labeled MDVs, washed, and imaged by ImageStreamX mark II (Amnis, Part of Luminex, Au. TX). Each value represents the mean ± SD for $n = 3$ biological repeats. Significant differences were detected using *t*-test, *P*-value = 0.018. Mito, mitochondria; Ves, MDVs.

Source data are available online for this figure.

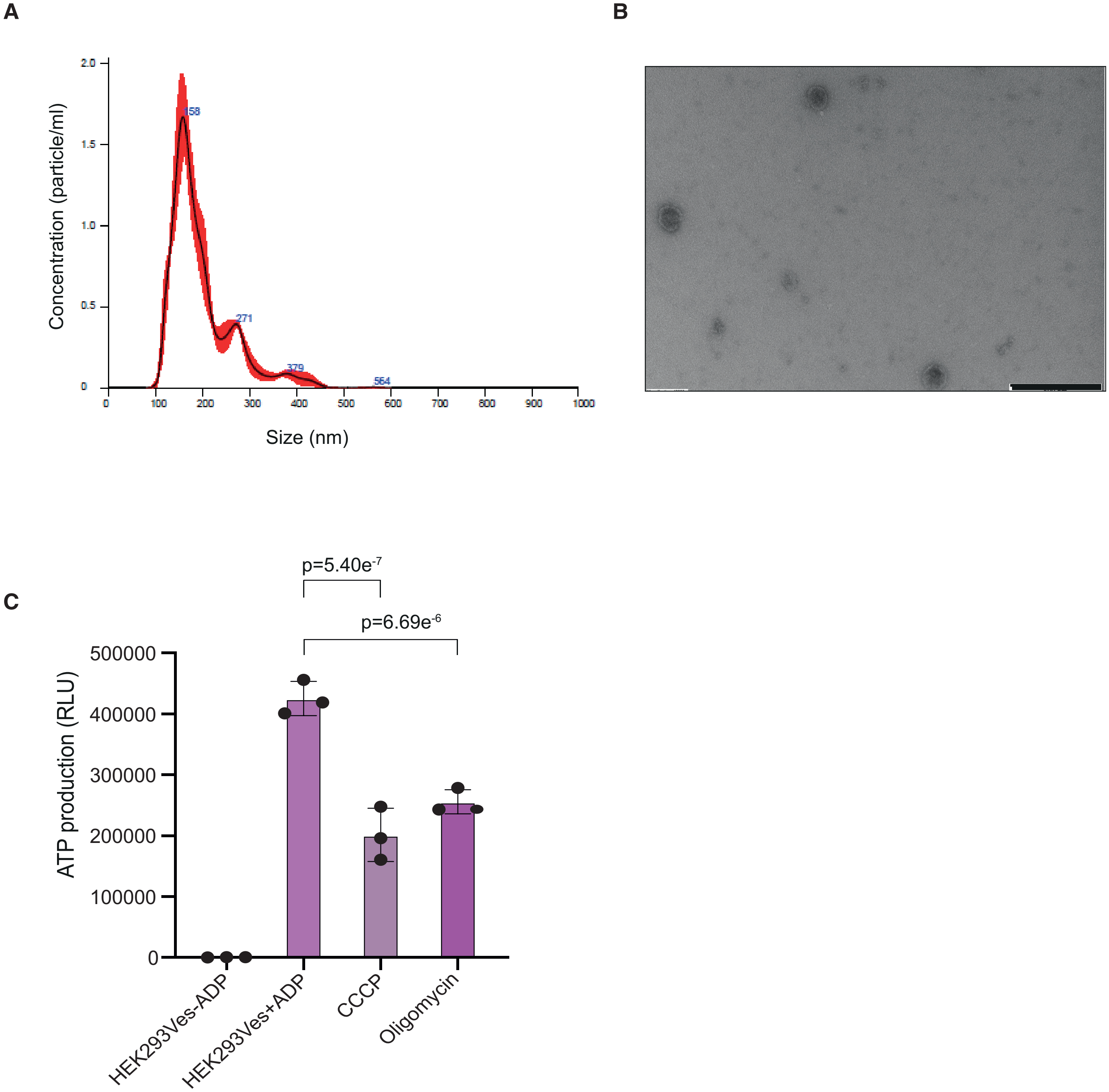

Figure EV5. MDVs derived from HEK293-isolated mitochondria.

A Nanoparticle tracking analysis of HEK293-derived MDVs. Vesicles derived from HEK293-isolated mitochondria were subjected to size distribution and concentration using nanoparticle tracking analysis (NTA).

B TEM images of HEK293-derived MDVs. Samples were stained with 2.0% uranyl acetate or 2.0% phosphotungstic acid. 5 μl of vesicles was placed on Formvar/carbon-coated copper 200 mesh grids (EMS), mixed with 5 μl of PTA for 10–20 s, while excess stain was blotted off and grids were dried. Samples were examined with Jeol (Jem-1400 Plus) transmission electron microscope. Scale bar—500 nm.

C HEK293-derived MDVs, produce ATP in an enzymatic reaction dependent on membrane potential. Mitochondrial-derived vesicles from HEK293-isolated mitochondria were incubated in the presence or absence of ADP, CCCP, or oligomycin for 15 min at 37°C. Subsequently, ATP was measured by luciferin-luciferase luminometry. Each value represents the mean ± SD for $n = 3$ biological repeats. Significant differences were detected using *t*-test, *P*-value = 6.69E-06/5.40E-07 for oligomycin and CCCP, respectively.

Source data are available online for this figure.

© 2023 Weizmann Institute of Science
