# Supplementary material for: Mitochondrial‐derived vesicles retain membrane potential and contain a functional ATP synthase
Source: EMBO Rep. 2023 Mar 17;24(5):e56114. doi: 10.15252/embr.202256114 (PMC10157309; doi:10.15252/embr.202256114)
Supplement: Supplementary file 8 — PDF+ [file EMBR-24-e56114-s001.pdf]

Report

SOURCE DATA | TRANSPARENT PROCESS | OPEN ACCESS

EMBO reports

Mitochondrial-derived vesicles retain membrane potential and contain a functional ATP synthase

Reut Hazan (Ben-Menachem)[1,†], Dvora Lintzer[1,†], Tamar Ziv[2], Koyeli Das[1], Irit Rosenhek-Goldian[3], Ziv Porat[4], Hila Ben Ami Pilo[5], Sharon Karniely[6], Ann Saada[7,*], Neta Regev-Rudzki[5,**] & Ophry Pines[1,***]

Abstract

Vesicular transport is a means of communication. While cells can communicate with each other via secretion of extracellular vesicles, less is known regarding organelle-to organelle communication, particularly in the case of mitochondria. Mitochondria are responsible for the production of energy and for essential metabolic pathways in the cell, as well as fundamental processes such as apoptosis and aging. Here, we show that functional mitochondria isolated from *Saccharomyces cerevisiae* release vesicles, independent of the fission machinery. We isolate these mitochondrial-derived vesicles (MDVs) and find that they are relatively uniform in size, of about 100 nm, and carry selective protein cargo enriched for ATP synthase subunits. Remarkably, we further find that these MDVs harbor a functional ATP synthase complex. We demonstrate that these vesicles have a membrane potential, produce ATP, and seem to fuse with naive mitochondria. Our findings reveal a possible delivery mechanism of ATP-producing vesicles, which can potentially regenerate ATP-deficient mitochondria and may participate in organelle-to-organelle communication.

Keywords ATP synthase; membrane potential; mitochondria; mitochondrial-derived vesicles; protein distribution

Subject Category Membrane & Trafficking, Metabolism

DOI 10.15252/embr.202256114 | Received 12 September 2022 | Revised 27 February 2023 | Accepted 28 February 2023 | Published online 17 March 2023

EMBO Reports (2023) 24: e56114

Introduction

Eukaryotic cells are defined by the existence of subcellular compartments and organelles, which allow the partitioning of various biochemical pathways out of the cytosolic milieu into discrete organelles. In this sense, each cellular compartment has a specific protein composition that is vital for its function. Molecules of one protein can be located in several subcellular locations, a phenomenon termed dual targeting or dual localization. These identical or nearly identical forms of proteins, localized to different subcellular compartments are termed echoforms or echoproteins (to distinguish them from isoforms/isoproteins; Yogev & Pines, 2011; Kalderon & Pines, 2014; Morgenstern *et al*, 2017). Dual targeting has been shown to be highly abundant and we now estimate that in yeast, more than one-third of the mitochondrial proteome is dual-targeted (Ben-Menachem *et al*, 2011; Kisslov *et al*, 2014). The vast majority of mitochondrial proteins are nuclear-encoded and are synthesized on cytosolic polysomes. A composite group of cytosolic and mitochondrial proteins takes part in recognition, targeting, and translocation of mitochondrial proteins from the cytosol to their destined organelle. Within mitochondria, proteins are sorted into one of their four sub-compartments: The outer membrane (OM), the intermembrane space (IMS), the inner membrane (IM), and the matrix. While there is thorough research on the import of proteins into mitochondria, protein export from the organelle is perceived as nonexistent (Neupert & Herrmann, 2007; Grevel *et al*, 2019; Schneider, 2020).

Mitochondria occupy a substantial portion of the cytoplasmic volume of eukaryotic cells, and they have been essential for the evolution of eukaryotes. Mitochondria fulfill many crucial functions in the eukaryotic cell such as oxidative ATP production, providing building blocks for biosynthesis of macromolecules, β-oxidation of fatty acids, heme biosynthesis, and formation of iron–sulfur (Fe-S) clusters (Reichert & Neupert, 2004). Apart from their normal physiological functions, mitochondria also play an important role in pathological phenomena in cells, including programmed cell death (apoptosis; Jiang & Wang, 2004) and aging (Balaban *et al*, 2005). All these reactions involve the flow of substrates and products between

1 Department of Molecular Genetics and Microbiology, IMRIC, Faculty of Medicine, Hebrew University, Jerusalem, Israel
2 Smoler Proteomics Center, Technion, Haifa, Israel
3 Departments of Chemical Research Support, Weizmann Institute of Science, Rehovot, Israel
4 Flow Cytometry Unit, Department of Life Sciences Core Facilities, Weizmann Institute of Science, Rehovot, Israel
5 Department of Biomolecular Sciences, Weizmann Institute of Science, Rehovot, Israel
6 Division of Virology, Kimron Veterinary Institute, Bet Dagan, Israel
7 Department of Genetics, Hadassah Medical Center and Faculty of Medicine, Hebrew University, Jerusalem, Israel
*Corresponding author. Tel: +972 26776844; E-mail: annsr@hadassah.org.il
**Corresponding author. Tel: +972 89343160; E-mail: neta.regev-rudzki@weizmann.ac.il
***Corresponding author. Tel: +972 26757203; E-mail: ophryp@ekmd.huji.ac.il
†These authors contributed equally to this work

© 2023 Weizmann Institute of Science. Published under the terms of the CC BY 4.0 license

the organelles within the cell, and direct inter-organellar contact is frequently required. Membrane contact sites (Scorrano *et al*, 2019) are a major mode of interaction between organelles and a means to maintain cellular homeostasis (Rizzuto *et al*, 1998; Zhang *et al*, 2005). Another possible mode of mitochondrial cross-talk was indicated by a report that mitochondrial cargo proteins are sorted into, at the time, uncharacterized mitochondrially-derived vesicles (Neuspiel *et al*, 2008). Mitochondrial-derived vesicles (MDVs) can be generated either from the outer membrane of mitochondria and may include both outer and inner membranes, and matrix content (Neuspiel *et al*, 2008; Soubannier *et al*, 2012a,b). These MDVs are defined by their diameter (between 70 and 150 nm), their independence of their formation on the mitochondrial fission GTPase Drp1 (Dnm1 in yeast), and by selective incorporation of protein cargo (Soubannier *et al*, 2012b). Of particular note is that MDVs formed *in vitro* are selectively enriched in oxidized cargo and are bound for late endosomes/lysosomes for degradation (Neuspiel *et al*, 2008; McLelland *et al*, 2014). In yeast and fungi, vesicles termed ectosomes were reported in 2007 (Rodrigues *et al*, 2007), but since then, little has been done to unravel the structure and function of these vesicles.

In the current study, we wished to gain an understanding regarding the function of MDVs in protein localization and signaling, and to this end, we developed a method to isolate the secreted vesicles directly from isolated and pure yeast mitochondria. We then confirm that yeast mitochondria can generate vesicles, which display selectivity in protein cargo including several ATPase subunits. Moreover, we describe for the first time the presence of an active ATPase machinery in these vesicles and their capability to generate ATP independently, and remarkably transfer this ability to other naïve mitochondria.

Results and Discussion

Isolated and purified mitochondria from *S. cerevisiae* generate vesicles

In order to determine the function of MDVs in signaling, it was essential to purify large amounts of these vesicles and determine their cargo. To this end, we characterized and investigated the role of MDVs from *S. cerevisiae*. Initially, we isolated mitochondria from yeast cells using standard isolation methods (Daum *et al*, 1982), following a further purification step by percoll gradient, in order to separate mitochondria from contaminating organelles (e.g., ER; Cunningham & Wickner, 1989; Trotter & Voelker, 1995). The purity of our isolated mitochondria was monitored with two methods—western blotting and LC/MS/MS. As shown in Fig 1A, the mitochondrial fraction is essentially free of cytosolic contamination as detected by antibodies against mitochondrial [Tim44 and Tim23 (Translocases of the Inner Mitochondrial membrane) Atp2 (Beta subunit of the F1 sector of mitochondrial F1F0 ATP synthase) and Nfs1 (Mitochondrial cysteine desulfurase)], cytosolic [hexokinase 1 (Hxk1) and Tdh1 (Glyceraldehyde-3-phosphate dehydrogenase (GAPDH), isozyme 1)] and peroxisomal [Peroxisomal importomer complex component (Pex13)] markers, indicating the efficiency of our isolation method. Moreover, based on our LC/MS/MS results, the summed intensity of the mitochondrial proteins in the isolated mitochondria fraction, was more than 99% of the total intensity of the proteins (Fig 1B and Table EV1), reinforcing the purity of our isolated mitochondria.

The quality of our isolated mitochondria was examined by an *in vitro* import assay (Daum *et al*, 1982) of the TCA cycle protein, aconitase (Aco1). In this experiment (Fig 1C), the labeled *in vitro*-transcribed Aco1 precursor was incubated with our pure mitochondria, and the efficiency of the import process was monitored by the existence of the mature form of the protein. As shown in Fig 1C, the mature form of the protein was detected after 2 min, while after 15 min, almost 50% of the protein population was processed, indicating that the mitochondria are intact and have a membrane potential. Taken together, these results indicate that our mitochondria are pure and active and suitable for MDV isolation.

Previous work (Neuspiel *et al*, 2008) suggested that MDVs should be defined by at least three criteria: (i) relatively uniform diameter of 70–150 nm, (ii), independence of the mitochondrial fission protein Drp1 (Dnm1 in yeast), and (iii) selective incorporation of protein cargo. We proceeded to isolate yeast MDVs (Fig 1D) and examined our results according to these criteria. In order to determine whether mitochondrial-derived vesicles are released from mitochondria, we used differential centrifugation to distinguish MDVs from intact mitochondria (Daum *et al*, 1982). Isolated and purified mitochondria were incubated in an isotonic buffer for 24 h and separated from the supernatant by centrifugation. The supernatant was freed from debris by viva-cell centrifugation (through vertical filtration membranes; Sisquella *et al*, 2017), which was then centrifuged at high speed (Fig 1D). Size and population analysis were determined by using four different strategies, as shown in Fig 2. Nanosight particle analysis, revealed a particle diameter distribution between 80 and 200 nm with a peak at 105.8 ± 2.2 nm, which corresponds to the typical size of mitochondrial vesicles (Fig 2A; Neuspiel *et al*, 2008). Imaging analyses of mitochondrial-derived vesicles by atomic force microscopy (AFM) and transmission electron microscopy (TEM), verified that these vesicles are within a range of 50 to 200 nm diameter (Fig 2B and C, respectively). Moreover, Cryo-transmission electron microscopy (cryo-TEM) showed that these vesicles are composed of single or potentially double membranes (Fig 2D) as described previously (Neuspiel *et al*, 2008). Together, these results indicate that vesicles released by isolated mitochondria are of the expected size. To determine whether mitochondrial vesicle formation requires the mitochondrial fission GTPase Dnm1, we isolated mitochondria from KO cells of this protein and repeated the vesicle isolation procedure. Size and population analysis were determined as described (Fig 2), and the results were similar to the *wild-type* strain (Fig EV1A–C), indicating vesicle scission does not require this fission machinery.

To further validate the physiological relevance of MDVs, we conducted experiments to isolate vesicles at 4 h, 24 h, and 48 h. As shown in Fig EV2A, our nanoparticle tracking analysis (NTA) measurements revealed that the concentration of MDVs reached 1.8×10^{11} particles/ml at 4 h, 8×10^{10} particles/ml at 24 h and remained unchanged at 48 h. This saturation trend suggests a decline in vesicle formation capacity over time. Together these results suggest that nonfunctional mitochondria (e.g., 48 h at 30°C) are incapable of MDV production and these results were further supported by measuring the ATP generation capacity of remnant mitochondrial (Fig EV2B). Our results indicated that after 4 h of incubation, the mitochondria retained 33% of their original ATP

 © 2023 Weizmann Institute of Science

production capacity, while after 24 h, the capacity was significantly reduced to 14% of that of fresh mitochondria. These findings provide further evidence that nonfunctional mitochondria (e.g., 48 h at 30°C) are unable to produce MDVs and that the remnant mitochondria are less active in terms of ATP production and vesicle formation.

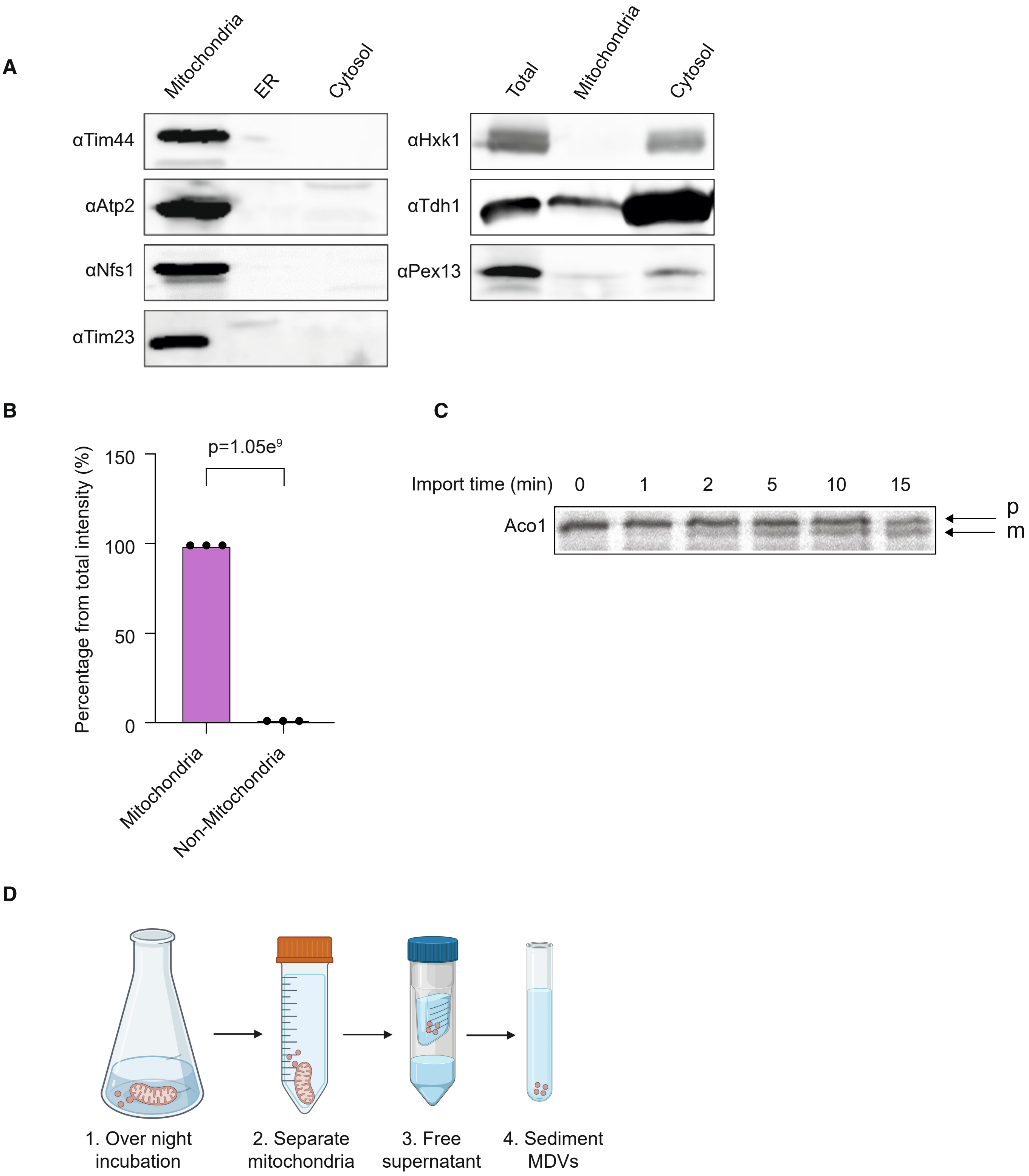

Figure 1.

Figure 1. Isolation and characterization of mitochondria from *Saccharomyces cerevisiae*.

A Western blot analysis of purified mitochondria. Mitochondria were isolated from yeast BY4741 wild-type strain, and their purity was assessed by western blot analysis using Tim44 (49 kDa), Tim23 (23 kDa), Nfs1 (54 kDa), and Atp2 (55 kDa) antisera as mitochondrial markers, Hxk1 (54 kDa) and Tdh1 (36 kDa) antisera as cytosolic markers and Pex13 (42.7 kDa) as a peroxisomal marker.

B Percentage of the summed intensity of mitochondrial proteins in the mitochondrial fractions according to LC/MS/MS analysis. Each value represents the mean ± SD for $n = 3$ biological repeats, P-value = 1.05e9.

C *In vitro* import of Aco1. Aco1 mRNA was translated in rabbit reticulocyte lysate in the presence of [^{35}S] methionine. The labeled protein products were added to purified mitochondria for the indicated times, in order to measure the mitochondrial import efficiency. Samples were analyzed by SDS–PAGE and autoradiography. Processing of the Precursor, p form to a Mature, m form, indicated a functional import machinery (Vitrobot mark IV, FEI).

D Representation of the vesicle isolation method. Isolated and purified mitochondria were incubated in an isotonic buffer (1) and separated from the supernatant by centrifugation (2). The supernatant was freed from debris by viva-cell centrifugation (through vertical filtration membranes; Sisquella *et al*, 2017; 3), which was then ultracentrifuged at high speed to sediment MDVs (4). The figure was generated by the BioRender software (https://app.biorender.com/).

Source data are available online for this figure.

MDVs show selectivity in protein cargo

It is well known that MDVs and extracellular vesicles (EVs) contain a variety of proteins and nucleic acid cargoes (Neuspiel *et al*, 2008; Abels & Breakefield, 2016; Ofir-Birin *et al*, 2017; Gezsi *et al*, 2019; Hernandez-Barranco *et al*, 2021). In order to determine the cargo selectivity of MDVs, isolated vesicles were purified by an OptiPrep (OP) gradient, an established method for EV purification (Regev-Rudzki *et al*, 2013), and fractions were loaded on acrylamide gels for validation of gradient efficiency. As shown in Fig 3A, fractions 3–5 in which most MDVs were concentrated, were chosen for further analysis by LC/MS/MS. Comparison between the LC/MS/MS results of three biological repeats (Fig 3B Venn diagrams and Fig EV3A, PCA plot) led to the identification of 520 mitochondrial proteins in the mitochondrial fractions, from which 444 were detected in all three repeats (Fig 3B, top right Venn diagram and Dataset EV1). The summed intensity of the mitochondrial proteins was more than 99% of the total intensity of the proteins. Our analysis revealed that out of these 520, only 239 mitochondrial proteins were identified in MDVs, from which 168 were detected in all three repeats (Fig 3B, top left and bottom Venn diagrams, Dataset EV2). The summed intensity of the mitochondrial proteins of the MDVs was more than 90% of the total intensity of the proteins (Table EV1).

This group of 168 mitochondrial proteins includes inner and outer membrane proteins, as well as matrix soluble proteins, suggesting that vesicle proteins are acquired from internal mitochondrial sub-compartments (Fig 3C). As the protein profile of the vesicles was not similar to the profile of the mitochondrial fractions, and the percentage of each protein from the total intensity of mitochondrial proteins in each sample was calculated. Comparison between the vesicles and the mitochondrial fraction revealed that proteins are unevenly distributed between those fractions. For example, the levels of ribosomal proteins in vesicle fractions were significantly lower than their levels in the mitochondrial fractions. Conversely, outer membrane proteins showed a higher percentage from the total in the vesicles (Fig 3D). Those differential protein profiles suggest that there is a selectivity of mitochondrial proteins in MDVs. Specific examination shows proteins that are clearly identified in all three mitochondrial samples but absent from MDV samples (Dataset EV3). Conversely, there are a relatively few proteins clearly detected in vesicles (e.g., Icl1, according to number of peptides) but on the threshold of detection in the mitochondrial fraction. Those differential protein profiles suggest the presence of selected mitochondrial proteins in MDVs.

MDV proteins affect vesicles formation and content

After characterizing the vesicles and examining their protein cargo, we attempted to approach the functional importance of such MDV proteins. Initially, we asked whether mutations in genes encoding for proteins present in MDVs would have an effect on the formation of these vesicles. In order to address this question, we isolated vesicles from two different KO strains; Mdh1 and Om45. Mdh1 is a mitochondrial matrix TCA enzyme while Om45 is an outer mitochondrial membrane protein. According to our mass-spec results, these proteins are found to be clearly detected in MDVs and represent different locations of mitochondrial proteins that exist in MDVs (Dataset EV2). After validating by western blot analysis, the presence of Mdh1 in MDVs (Fig EV2C), we compared MDVs levels generated by these KO strains versus wild-type strains, by Nanosight (NTA) particle analysis (Gezsi *et al*, 2019; Dekel *et al*, 2021). As shown in Fig 3E, vesicle concentration was lower in the two KO strains, as compared to the wild-type strain (P-value < 0.05), suggesting that these proteins may affect vesicle formation. In contrast, vesicle concentration was not reduced in ΔDnm1, which is not expected to influence vesicle production and concentration (as shown in Fig EV2D). Nevertheless, the effect of these KO mutations is most probably indirect and caused by effects on general mitochondrial function.

Functional importance of MDVs

It is well known that vesicles can be transferred between cells and function in many different cellular activities, such as cell–cell communication, virulence, cargo, genetic material transfer, etc, but less is known regarding the functions of MDVs (Andrade-Navarro *et al*, 2009; Ryan *et al*, 2020; Picca *et al*, 2022). To assess the functional importance of MDVs, we performed a GO-annotation enrichment—analysis of biological processes using the STRING software, on the 168 mitochondrial proteins that were identified in our MDVs. As shown in Fig 4A and Dataset EV4, a number of metabolic and biosynthetic pathways are represented in the vesicle proteome, while the ATP production-related processes were one of the most significant. The mitochondrial F1Fo-ATP synthase produces the bulk of cellular ATP, and in yeast, it consists of 17 different structural subunits of dual genetic origin (Song *et al*, 2018). Thus, we decided to focus on this fascinating complex which comprises complex V of the mitochondrial respiratory chain. According to our mass-spec results, most subunits were detected in MDVs (13

 © 2023 Weizmann Institute of Science

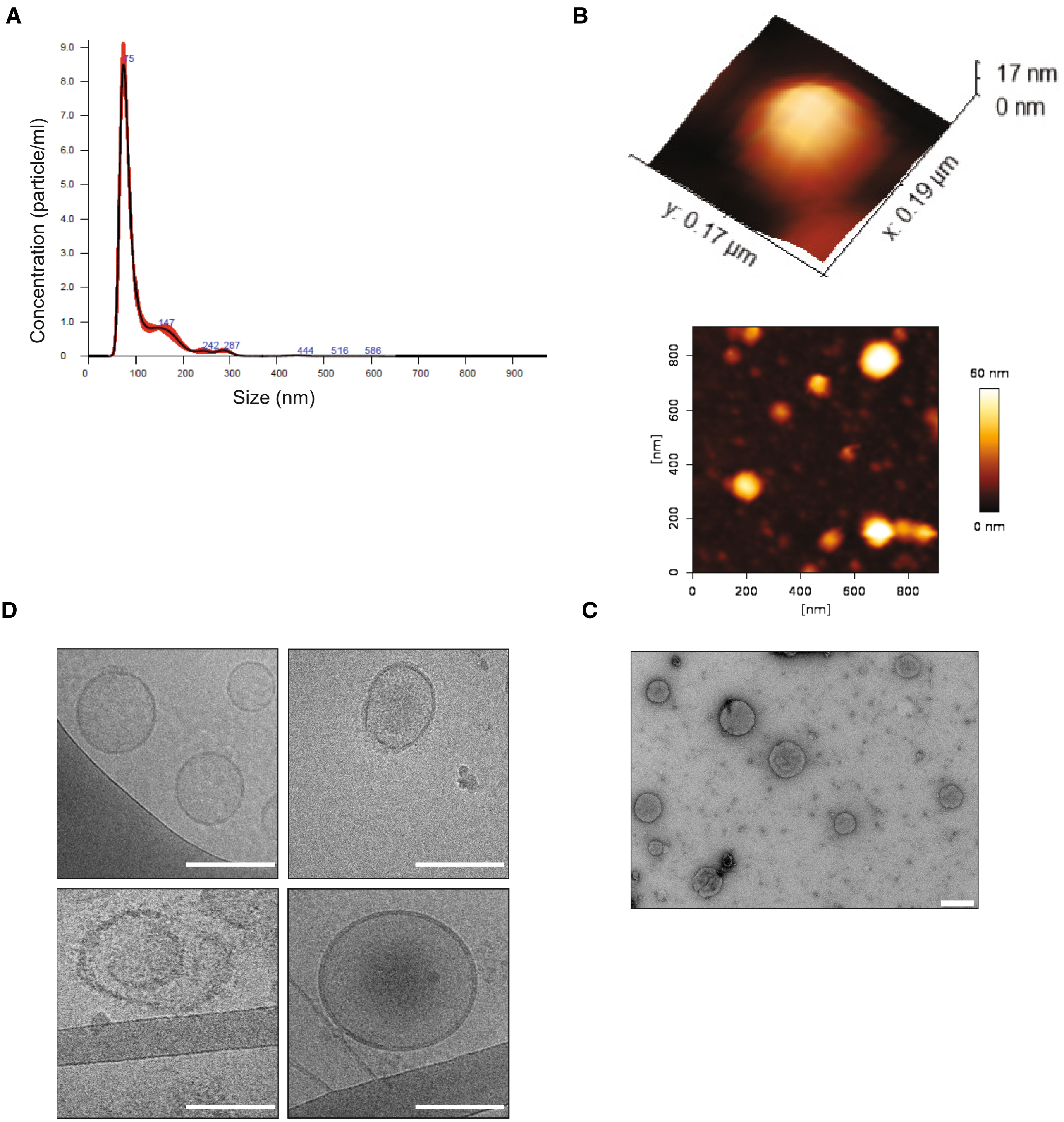

Figure 2. Characterization of MDVs from wild-type mitochondria.

A Nanoparticle tracking analysis. Vesicle size distribution and concentration were performed using nanoparticle tracking analysis (NTA; Malvern Instruments, Nanosight NS300). Sample size distributions were calibrated in a liquid suspension by the analysis of Brownian motion via light scattering. Nanosight provides single particle size and concentration measurements.

B Atomic force microscopy. Representative AFM image and a 3D AFM image of one representative vesicle (WT), adsorbed on a mica modified with Mg^{2+} and imaged under PBS.

C TEM images of MDVs. Samples were stained with 2.0% uranyl acetate or 2.0% phosphotungstic acid. 5 µl of vesicles was placed on Formvar/carbon-coated copper 200 mesh grids (EMS), mixed with 5 µl of PTA for 10–20 s, while excess stain was blotted off and grids were dried. Samples were examined with Jeol (Jem-1400 Plus) transmission electron microscope. Scale bar—200 nm.

D Cryo-TEM analysis of mitochondrial-derived vesicles, which can be detected to contain single and potentially double membranes. Samples were prepared using a vitrification robot system. Scale bar—100 nm.

Source data are available online for this figure.

© 2023 Weizmann Institute of Science

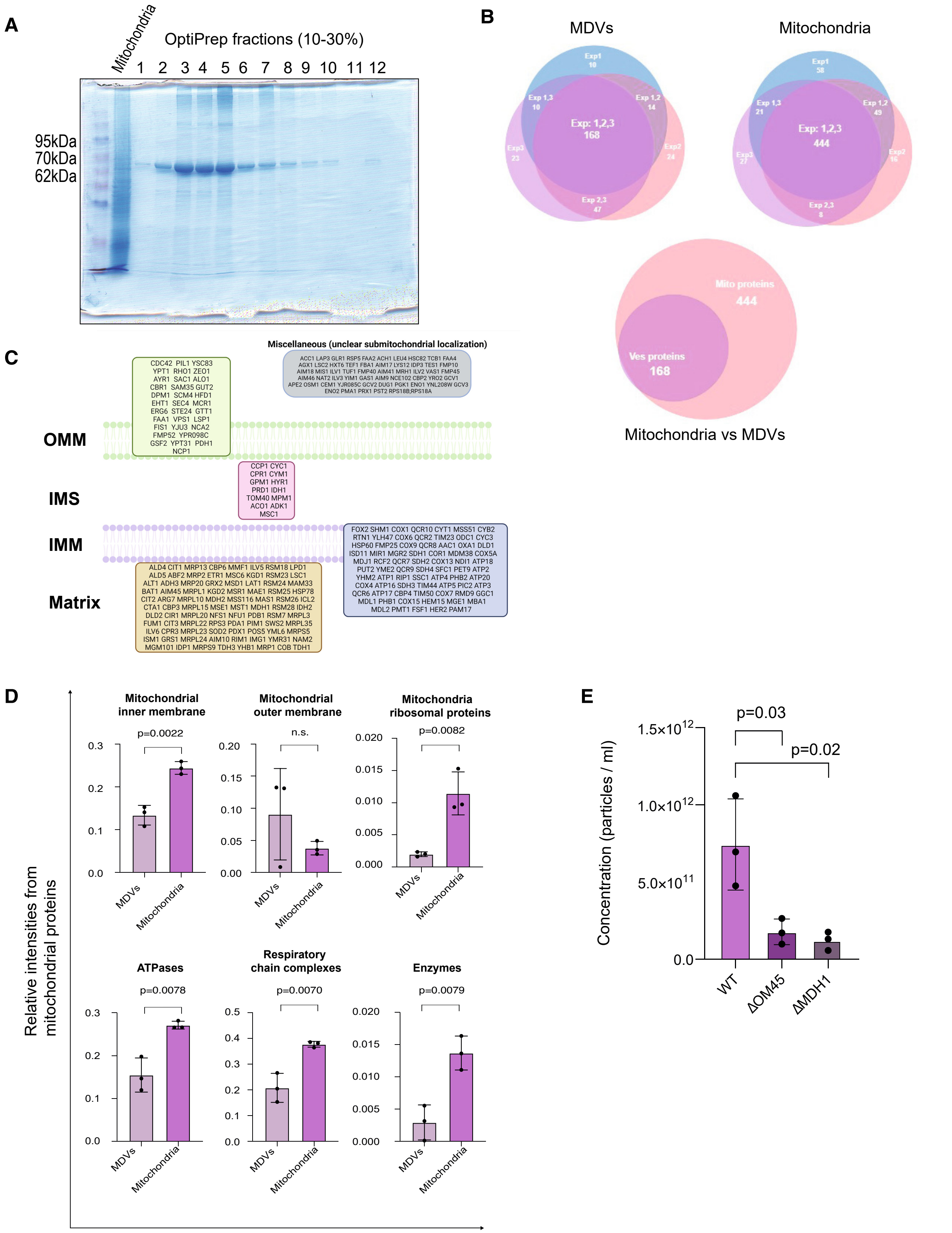

Figure 3.

© 2023 Weizmann Institute of Science

Figure 3. Selectivity and specificity of MDV protein cargo as shown by LC/MS/MS analysis.

A OptiPrep gradient purification of MDVs. Media components were fractionated by centrifugation (100,000 *g*, 18 h, 4°C) through a continuous 10–30% OptiPrep (Axis-Shield) gradient. Fractions (1 ml) were collected from the top of the gradient and run on acrylamide gel for further analysis.

B Venn diagram of three different repeats of LC/MS/MS analysis. 444 mitochondrial proteins were observed in mitochondrial fractions from three different repeats, and out of this group, 168 mitochondrial proteins were observed in MDVs.

C Representation of sub-compartmental localization of the MDV proteome. OMM—outer mitochondrial membrane; IMM—inner mitochondrial membrane; IMS—inter-membrane space.

D There is a selectivity in protein cargo of MDVs versus mitochondrial fractions. Percentage of representative mitochondrial proteins from the total intensity of mitochondrial proteins from three different repeats was calculated. EV = MDVs. Mito = mitochondria. Each value represents the mean ± SD for $n = 3$ biological repeats. Mitochondrial inner membrane *P*-value = 0.0022. Mitochondrial outer membrane *P*-value = n.s. Mitochondria ribosomal proteins *P*-value = 0.0082. ATPases *P*-value = 0.0078. Respiratory chain complexes *P*-value = 0.0070. Enzymes *P*-value = 0.0079.

E Compromised mitochondria produce less MDVs. MDVs isolated from Wild-type and KO strains (ΔomM45 and Δmdh1) were analyzed using nanoparticle tracking analysis (NTA; Malvern Instruments, Nanosight NS300). Each value represents the mean ± SD for $n = 3$ biological repeats. Significant differences were detected using *t*-test, *P*-value = 0.033/0.023 for ΔOM45 and ΔMdh1, respectively.

Source data are available online for this figure.

subunits, Dataset EV2). To verify the presence of some of these subunits, we subjected yeast MDVs to western blot analysis, with anti-Atp2 antibody, which is a crucial structural subunit of the mitochondrial ATP synthase. As shown in Fig 4B, we detected Atp2 in all three main OptiPrep fractions of the secreted vesicles. Additionally, we examined the presence of Atp2 and Atp1 in vesicles by using immunogold labeling (Fig 4C). Isolated vesicles were first incubated with anti-Atp1 or anti-Atp2 antibodies, and then with 12 nm gold goat anti-rabbit antibodies. Samples were inspected by transition electron microscopy and as shown in Fig 4C, our results confirmed that both Atp1 and Atp2 are present in vesicles, as compared to the appropriate controls (without the first antibody and anti-Hxk1 antibody, cytosolic protein, nonmitochondrial). Moreover, the average number of gold particles of Atp2 was significantly higher compared with the controls [P-value < 0.05] (Fig EV3B). Together, these results further support the presence of F1Fo-ATP synthase subunits in our vesicles.

We hypothesized that if MDVs contain functional F1Fo-ATP synthase in their membranes, it is possible that they may be able to generate ATP. If this intriguing possibility is true, one may ask whether vesicles would be capable to transfer this ability to generate ATP between healthy and damaged mitochondria and rescue their phenotype. To address these issues, isolated MDVs from *wild-type* mitochondria were incubated with or without ADP in sorbitol buffer isotonic for mitochondria, and after washing and centrifugation steps, ATP production was determined by luminometry using luciferin-luciferase. As shown in Fig 5A, during the 15 minutes incubation time in 30°C, a significant amount of ATP was produced compared with the control (no added ADP). Notably, a decreased amount of ATP was produced at 4°C compatible with an enzymatic reaction. Moreover, in the presence of the uncoupler CCCP or the ATP synthase (complex V) inhibitor oligomycin, the reaction was partially but significantly inhibited, indicating mitochondrial ATP production. Furthermore, insignificant amounts of ATP were detected in the second wash, suggesting that no MDVs remained in the reaction mix. Taken together these results indicate that yeast MDVs can produce ATP in an enzymatic reaction, which is dependent on F1Fo-ATP synthase and membrane potential. When comparing the ATP production capacity of MDVs (140,260 ± 4,623 RLU/min/μg protein) to mitochondria (246,496 ± 7,824 RLU/min/μg protein), MDVs retained 57% capacity when normalized to protein content.

The existence of membrane potential was also corroborated using image stream analysis, as shown in Fig 5B. Atp2-GFP vesicles treated with TMRE (tetramethylrhodamine, ethyl ester) showed the accumulation of this dye, and moreover, adding the uncoupler CCCP, reduces TMRE accumulation, indicating the existence of an electrochemical proton gradient. While setting up the ATP production experiments, we noted to our surprise, that the addition of TCA cycle substrates such as succinate, pyruvate or glycerol had no or insignificant effects on vesicle ATP production (Fig 5A). Moreover, MDVs from yeast devoid of malate dehydrogenase (a TCA cycle enzyme) were able to efficiently drive the production of ATP (Fig 5A). Accordingly, we deduce that the membrane potential, *per se*, was sufficient to drive ATP production.

To assess whether wild-type MDVs were able to compensate for impaired ATP production of F1Fo-ATP synthase deficient mitochondria, we preincubated wild-type MDVs with wild-type mitochondrial versus Atp2 respiratory deficient mitochondria (ΔAtp2), prior to the ATP production assay. As shown in Fig 5C, ATP production by wild-type mitochondria increased to some extent (30%). The major finding here was the effect of wild-type MDVs on ΔAtp2 mitochondria, which increased more than 5-fold (560%; Fig 5C). Regretfully we could not perform the reciprocal experiments because we were not able to isolate (despite several attempts) MDVs from ΔAtp2 and we assume that most probably an intact F1Fo-ATP synthase may influence MDV production. Importantly, mitochondria were washed twice prior to the ATP production measurements for all the complementation experiments. In order to validate the efficiency of the washes and to demonstrate that no vesicles are detected after the second wash, we measured uptake efficiency by using image stream analysis. As can be shown in Fig EV4, mCherry-labeled mitochondria and GFP-labeled MDVs show significant co-localization, suggesting that the mitochondria and the vesicles are indeed co-localized.

Taken together, these results suggest that MDVs may rejuvenate damaged mitochondria by sending MDVs between healthy and damaged mitochondria. To corroborate some of the above results in another system, we were able to isolate MDVs from isolated mitochondria derived from HEK293 cells and analyzed their size by Nanosight particle analysis. As shown in Fig EV5A, this analysis revealed a typical size of mitochondrial vesicles (Neuspiel *et al*, 2008). Moreover, imaging analyses of mitochondrial-derived vesicles through transmission electron microscopy (TEM) verified that these vesicles are within a range of 50–200 nm diameter (Fig EV5B). Next, we measured ATP production in HEK293-derived MDVs and, in accord, we observed ATP production, which was also significantly decreased in the presence of CCCP or oligomycin (Fig EV5C).

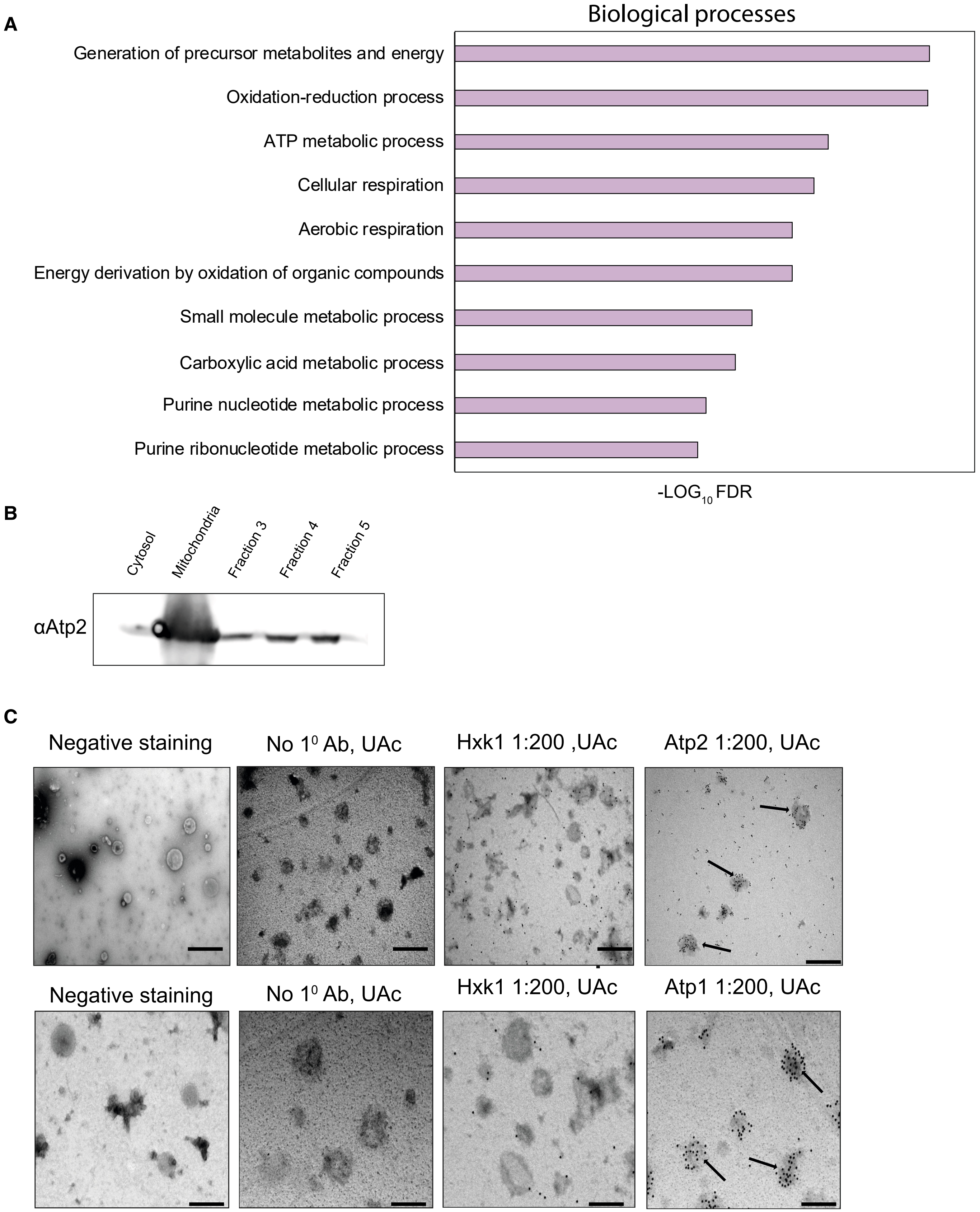

Figure 4.

© 2023 Weizmann Institute of Science

Figure 4. F1Fo-ATP synthase subunits are present in MDVs.

A GO annotation of biological processes of the MDV proteome. A list of 168 MDV proteins (based on three different repeats) was analyzed by the "STRING" tool. The results were filtered by strength > 0.5 and FDR < 0.05. Here, we represent the top 10 high significant values.

B Western blot analysis of gradient purified MDVs. Media components were fractionated by centrifugation (100,000 *g*, 18 h, 4°C) through a continuous 10–30% OptiPrep (Axis-Shield) gradient. Fractions (1 ml) were collected from the top of the gradient, ran on acrylamide gels, and assessed by western blot analysis using Atp2 antisera.

C Immunogold labeling of MDVs. Isolated mitochondrial-derived vesicles were incubated with specific primary antibodies (anti-Atp1 or anti-Atp2) and secondary antibodies conjugated to 12 nm gold (goat anti-rabbit). Samples were viewed by Tecnai 12 TEM 100 kV (Phillips, Eindhoven, the Netherlands). AUc—uranyl acetate. Scale bar—500 nm. Arrows indicate MDVs.

Source data are available online for this figure.

One of the challenges when dealing with the upcoming field of MDVs is not only to define their composition and specific structure but also as we show here demonstrate an activity and function associated with MDVs. This gives us confidence against the arguments that in some cases MDVs are simply ruptured degradation products of mitochondria.

A recent study (D'Acunzo *et al*, 2022) describes a detailed protocol for the isolation of EVs from both murine and human brains. By using an iodixanol-based gradient, the authors were able to separate distinct EV species including subpopulations of mitovesicles. Here we describe a novel and different method for the isolation of MDVs directly from isolated mitochondria and hence, they may not be identical to those generated in the cytoplasm.

Initially, we characterized the proposed and highly purified MDVs by a number of approaches (NTA—nanoparticle tracking analysis; TEM—transmission electron microscopy; AFM—atomic force microscopy [3D]; Cryo-TEM analysis). These MDVs indeed resemble classical mitochondrial secreted vesicles and have a relatively uniform size of about 100 nm with single or double membranes. Another important point worth discussing is that less than half of the identified mitochondrial proteome were detected in MDVs. Specific examination of this list of proteins shows that there are 72 proteins were nicely identified in all three experiments but are absent from MDVs. These results suggest that there appears to be a selection of mitochondrial proteins as vesicle cargo and this finding is consistent with the proteins identified in mitovesicles (D'Acunzo *et al*, 2022). A recent study (Konig *et al*, 2021) showed that DRP1 (Dnm1 in yeast) is required for TOM20-positive MDVs (albeit at lower levels than for fission). Based on this study, we were able to detect MDVs in ΔDnm1 yeast cells, as shown in Figs 2 and EV1 and as described in earlier publications (Neuspiel *et al*, 2008). Moreover, based on our LC/MS/MS, Tom20 is not detected in our MDVs, which may imply that in yeast the formation mechanism of MDVs may be different than in high eukaryotic cells. Of the number of biological processes, represented in the vesicle proteome, the mitochondrial F1Fo-ATP synthase was clearly outstanding. According to our results, most subunits were detected by mass-spec of MDVs (13 subunits, Dataset EV2) and we confirmed the presence of Atp2 by western blot analysis. Additionally, we detected the presence of Atp2 and Atp1 in vesicles by immunogold labeling.

The major discoveries in this study are that MDVs have a membrane potential and a functional F1Fo-ATP synthase complex with the capability to produce ATP. Like mitochondrial complex V, ATP production of MDVs is sensitive to membrane potential dissipaters and ATP Synthase inhibitors. Thus, MDVs acquire a major activity and function of mitochondria and moreover may possibly transfer this ability from one organelle to the other. This process may allow the rejuvenation of damaged mitochondria. In addition, ATP-producing vesicles may be directed to subcellular locations that require ATP such as the yeast bud or along axons in neurons without the need to translocate whole mitochondria. Another line of thought involves protein targeting or as alluded to earlier, MDVs may be a pathway to export proteins from mitochondria for which such a pathway has not been identified by any other mechanism. In fact, our group has been interested in protein dual targeting or dual localization of mitochondrial proteins and MDVs may be a mechanism by which this can achieve. In this regard, there are reports identifying ATP synthase in the plasma membrane, which could be achieved via an MDV-driven pathway (Cavelier *et al*, 2012). We know (According to our data) that mutations in some mitochondrial protein-encoding genes (e.g., MDH1, OM45) can affect the number of vesicles produced by mitochondria but assume that this is an indirect effect resulting from general mitochondrial wellbeing. We have no idea at present which proteins are mechanistically involved

Figure 5. MDVs produce ATP and can rejuvenate damaged mitochondria.

A MDVs produce ATP in an enzymatic reaction dependent on membrane potential. Mitochondrial-derived vesicles from wild-type (wtMDVs) or devoid of malate dehydrogenase (dMDH MDVs), were incubated in the presence or absence of ADP, CCCP, or oligomycin for 15 min at 30°C or 4°C. Subsequently, ATP was measured by luciferin-luciferase luminometry. Each value represents the mean ± SD for $n = 3$ biological repeats. Significant differences were detected using *t*-test, *P*-value = 0.01/ 0.0004 for CCCP and oligomycin, respectively.

B Membrane potential of MDVs as visualized by imaging flow cytometry. Atp2-GFP MDVs stained with TMRE (tetramethylrhodamine, ethyl ester) were treated with CCCP and imaged by ImageStreamX mark II (Amnis, Part of Luminex, Au. TX). Each value represents the mean ± SD for $n = 3$ biological repeats. Significant differences were detected using *t*-test, *P*-value = 0.0039.

C MDVs can be taken up by mitochondria and rejuvenate damaged mitochondria. Isolated mitochondria from wild-type (WT mito) and respiratory deficient mitochondrial (dATP2 mito) were preincubated in the presence or absence of mitochondrial-derived vesicles from wild-type (WT MDVs), washed and incubated for 15 min at 30°C in the presence or absence of ADP. Subsequently, ATP formation was measured by luciferin-luciferase luminometry. Each value represents the mean ± SD for $n = 3$ biological repeats. Significant differences were detected using *t*-test, *P*-value = 2.38872E-13 (for WT mito+ADP)/ 5.44794E-06 (for WT mito + ADP + MDVs) 2.38872E-13 (for Δatp2 mito + ADP)/ 4.84404E-13 (for Δatp2 mito + ADP + MDVs).

Source data are available online for this figure.

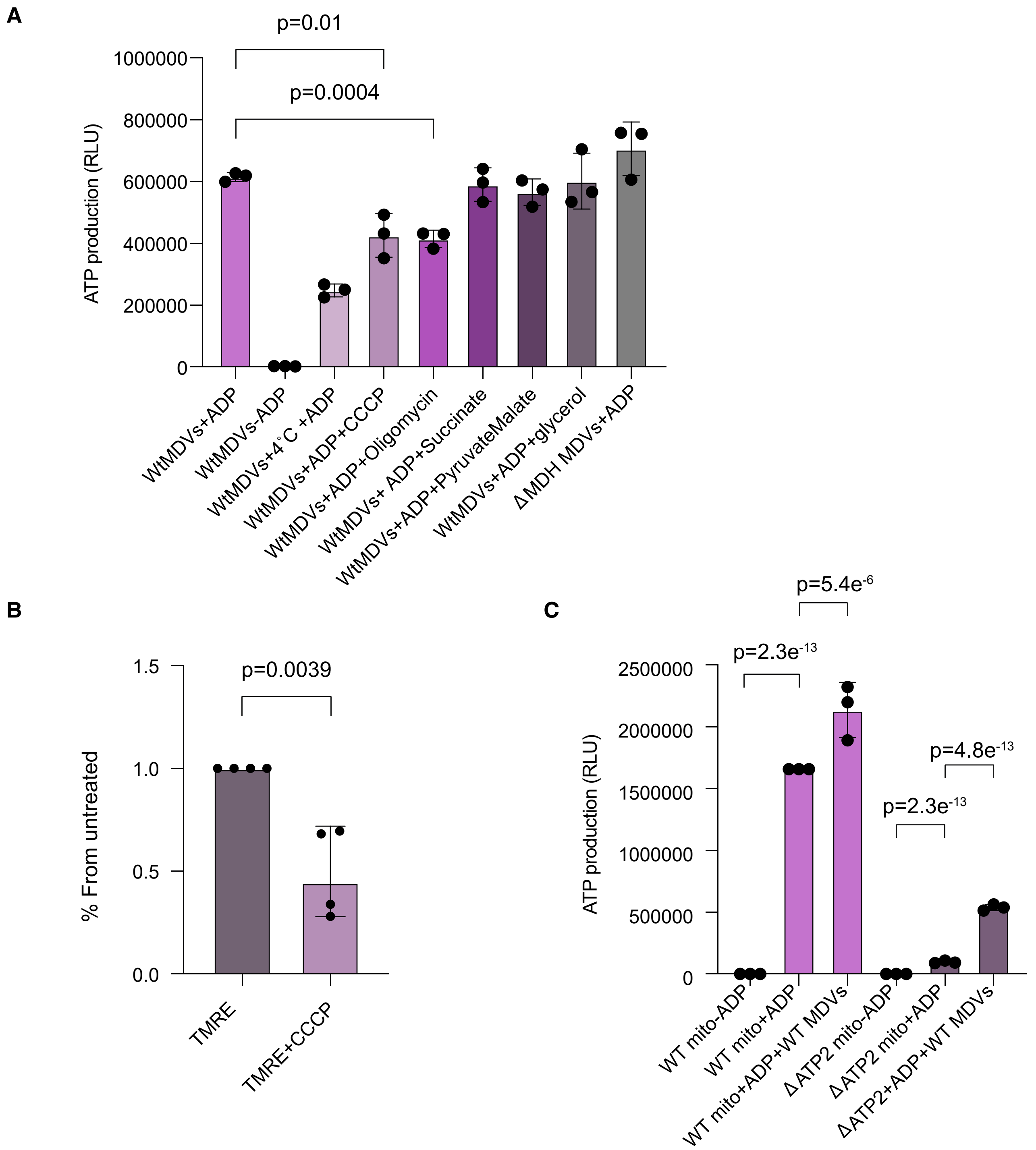

Figure 5.

in MDV formation. Recent studies demonstrated the importance of MDV formation in mitochondrial quality control and immune response (Matheoud *et al*, 2016; Konig *et al*, 2021; Todkar *et al*, 2021) In this study, we have focused on protein biochemistry, cell biology, and function of MDVs as a mean of ATP-producing agents; however, there are many other aspects of MDV biology, which will be addressed by our group in future.

Materials and Methods

Yeast strains

Saccharomyces cerevisiae strains used were: BY4741 (Mat a, his31, leu20, met150 and ura30), ΔDnm1 (BY4741; MATa; his3Δ1; leu2Δ0; met15Δ0; ura3Δ0; LL001w::kanMX4), ΔOm45 (BY4741; MATa;

 © 2023 Weizmann Institute of Science

his3Δ1; leu2Δ0; met15Δ0; ura3Δ0; YIL136w::kanMX4), ΔMdh1 (BY4741; MATa; his3Δ1; leu2Δ0; met15Δ0; ura3Δ0; YKL085w::kanMX4), ΔAtp1 (BY4741; MATa; his3Δ1; leu2Δ0; met15Δ0; ura3Δ0; YBL099w::kanMX4), ΔAtp2 (BY4741; MATa; his3Δ1; leu2Δ0; met15Δ0; ura3Δ0; YJR121w::kanMX4), Atp2-GFP (BY4741; MATa; his3Δ1; leu2Δ0; met15Δ0; ura3Δ0; ATP2-GFP::HIS).

Growth media

In order to isolate mitochondria, all strains were grown overnight at 30°C in synthetic depleted (SG) medium containing 0.67% (w/v) yeast nitrogen base, 2% (w/v) galactose medium, supplemented with the appropriate amino acids (50 µg/ml). The next day the cells were washed and transferred to lactate medium containing 0.67% (w/v) yeast nitrogen base, 2% (v/v) lactate, 0.05% (w/v) glucose, supplemented with the appropriate amino acids (50 µg/ml), and adjusted to pH 5.5 with KOH.

Western blot assay

Proteins were separated on 10% SDS–PAGE and transferred to nitrocellulose membranes. The primary antibodies used for detection were generously provided by Alexander Tzagoloff (Columbia University, USA) [anti-Atp1 and anti-Atp2] and Doron Rappaport (University of Tubingen, Germany) [anti-Mdh1 and anti-Por1]. Anti-Hxk1 was obtained commercially from ROCKLAND (catalog number 100-4159) and anti-Hsp60 was generated in our lab. Secondary antibodies used are anti-Rabbit IgG-HPR (111-035-003, Jackson, dilution 1:10,000). Western blot analyses were repeated at least three times. Image collection was performed using the ThermoScientific MyECL Imager V. 2.2.0.1250 and Amersham Imager 680 (GE Healthcare Life Sciences).

In vitro translation and import into mitochondria

Transcription of pGEM plasmids *in vitro* was carried out according to the manufacturer's instructions (Promega) using SP6 polymerase. Isolated mitochondria were prepared from yeast as described (Daum *et al*, 1982). Protein synthesis was performed in the presence of [^{35}S]methionine in rabbit reticulocyte lysate (Promega). Coupled translation/translocation reactions of 20 µl contained 16.5 µl of reticulocyte lysate, 0.5 µl of amino acid mix minus methionine, 0.5 µl of ribonuclease inhibitor, 1 µl of transcription reaction, and 10 µCi of [^{35}S]methionine. In all experiments, translation was allowed to proceed for 2 min (unless otherwise indicated) before the addition of mitochondria (2–2.5 mg) and the reaction further incubated at 25°C for 0, 1, 2, 5, 10, and 15 min. Following translation, all samples were diluted into SHKCl buffer (600 mm sorbitol, 50 mm Hepes, pH 7.2, 80 mm KCl). Centrifugation at 12,000 *g* for 10 min at 4°C yielded a supernatant fraction, and a mitochondrial pellet, which was washed once before being resuspended in SHKCl buffer. Samples were denatured by boiling in loading buffer containing 1% SDS, 4% β-mercaptoethanol, and 1% dithiothreitol and analyzed on 10% SDS–polyacrylamide gel electrophoresis followed by visualization with the image-analyzing system BAS2000 (Fuji Corp.).

Purification of mitochondrial-derived vesicles

Briefly, yeast mitochondria were purified and isolated as described previously (Daum *et al*, 1982). Next, isolated and purified mitochondria were resuspended in SH buffer (0.6 M sorbitol, 20 mM hepes pH 7.4) overnight at 30°C. Hek 293 Cell mitochondria were purified from 18 confluent T75 flasks of HEK293T (ATCC) cells grown in DMEM with 10% fetal bovine serum supplemented with glutamate and penicillin streptomycin in 5% CO_2 at 37°C. The cells were detached by cell scraper and washed twice in phosphate-buffered saline by centrifugation at 1,800 *g* at room temperature. The resulting cell pellet was suspended in 2 ml ice-cold buffer A (320 mM sucrose, 5 mM Tris, 2 mm EGTA, pH 7.4) and homogenized on ice by 300 strokes at 400 rpm with a potter-elvehjem glass-teflon homogenizer. Nuclei were removed by 3 min centrifugation at 2,000 *g*, 4°C, and mitochondria were pelleted from the supernatant by 10 min centrifugation at 12,000 *g*. After an additional wash, the mitochondria were suspended in 10 ml buffer A containing 2 mg/ml fatty acid-free bovine serum albumin and incubated overnight in 5% CO_2 at 37°C. Subsequently, mitochondrial pellets were removed by centrifugation at 18,000 *g* by two repetitive centrifugations. The supernatant was concentrated using a Vivaflow 100000 MWCO PES (Sartorious Stedium) and centrifuged at 150,000 *g*, 18 h to pellet MDVs. Aliquots were made and MDVs were stored at −20°C until further use.

OptiPrep gradient purification of MDVs

The initial vesicle pellet was resuspended in 2 ml SH buffer and further purified on a 10 ml continuous 10–30% OptiPrep gradient made up of NTE buffer (137 mM NaCl, 1 mM EDTA and 10 mM Tris, pH 7.4), 150,000 *g*, 18 h, 4°C, in an SW41 rotor (Beckman Coulter, Fullerton, CA, USA; Meningher *et al*, 2020). Fractions (1 ml) were collected from the top and 4 ml of Methanol were added before freezing MDVs at −80°C for 18 h. Each fraction was centrifuged for 30 min at 4°C, at 10,000 *g* and pellets were taken for further analysis.

Nanosight particle analysis (NTA)

MDVs size distribution and concentration were performed using nanoparticle tracking analysis (NTA; Malvern Instruments, Nanosight NS300). Sample size distributions were calibrated in a liquid suspension by the analysis of Brownian motion via light scattering. Nanosight provides single particle size and concentration measurements.

Atomic force microscopy

Freshly cleaved mica surface was incubated with 10 mM $MgCl_2$ solution for 2 min, then rinsed with 200 µl PBS. 50 µl of MDV solution was placed on the Mg-modified mica for 10 min. An additional 50 µl PBS solution was added to the sample prior to scanning. Images were captured using the JPK Nanowizard III AFM microscope (Berlin, Germany) in QI mode using qp-BioAC-CI-CB-2 or CB-1 probes, spring constants ≈ 0.1–0.2 N/m (Nanosensors). Image analysis was performed using JPK-SPM data processing software and Gwyddion software (Nečas & Klapetek, 2012).

© 2023 Weizmann Institute of Science

Transition electron microscopy

Samples were fixed with Karnovsky's fixative followed by staining with 2.0% of uranyl acetate. Briefly, 5 μl of MDVs was placed on Formvar/carbon-coated copper 200 mesh grids (EMS) and mixed with 5 μl of uranyl acetate for 10–20 s. Excess stain was blotted off and grids were dried. Samples were examined with Jeol® JEM-1400 Plus TEM (Jeol®, Tokyo, Japan), equipped with ORIUS SC600 CCD camera (Gatan®, Abingdon, United Kingdom), and Gatan Microscopy Suite program (DigitalMicrograph, Gatan®, UK).

Negative staining of MDVs

Vesicles were isolated from mitochondria and resuspended in SH buffer (0.6 M sorbitol, 20 mM hepes pH 7.4). The vesicles were adsorbed to Carbon–Formvar-coated copper grids. Grids were stained with 1% (w/v) uranyl acetate and air-dried. Samples were viewed with Tecnai 12 TEM 100 kV (Phillips, Eindhoven, the Netherlands) equipped with MegaView II CCD camera and Analysis® version 3.0 software (Soft Imaging System GmbH, Münstar, Germany).

Immunolabeling of MDVs

Isolated vesicles were adsorbed to carbon-coated nickel grids. From this point, all steps were performed in drops of different reagents on parafilm within a wet chamber. Grids were washed 5 times in a Tris-buffered saline (TBS) containing 20 mM Tris-base, 0.9% NaCl, 0.5% BSA, 0.5% Tween-20, and 0.13% NaN3, pH 8.2, and then incubated 30 min in a blocking solution containing 5% normal goat serum (NGS) in TBS. Grids were then exposed to antibodies for 16 h at 4°C. The antibodies were diluted 1:200 with 1% NGS in TBS. Girds were then washed five times with TBS. For immunogold labeling grids were incubated with 12 nm colloidal gold goat anti-rabbit (Jackson ImmunoResearch Laboratories) for 1.5 h followed by five washes in TBS and two washes in H_2O and then by a 2 min fixation of 2% glutaraldehyde. Contrasting was achieved with saturated aqueous uranyl acetate and lead citrate before air-drying. Samples were viewed as described for negative staining.

Proteolysis and mass spectrometry analysis

The proteins in the gel were reduced with 3 mM DTT in 100 mM ammonium bicarbonate [ABC] (60°C for 30 min), modified with 10 mM iodoacetamide in 100 mM ABC (in the dark, room temperature for 30 min), and digested in 10% acetonitrile and 10 mM ammonium bicarbonate with modified trypsin (Promega) at a 1:10 enzyme-to-substrate ratio, overnight at 37°C. The resulted peptides were desalted using C18 stage tips Uniprot and were analyzed by LC/MS/MS. The peptides were resolved by reverse-phase chromatography on 0.075 × 180-mm fused silica capillaries (J&W) packed with Reprosil reversed-phase material (Dr Maisch GmbH, Germany). They were eluted with linear 60 min gradient of 5–28% 15 min gradient of 28–95% and 15 min at 95% acetonitrile with 0.1% formic acid in water at flow rates of 0.15 μl/min. Mass spectrometry was performed by Q Exactive plus mass spectrometer (Thermo) in a positive mode (m/z 300–1,800, resolution 70,000 for MS1 and 17,500 for MS2) using repetitively full MS scan followed by collision induces dissociation (HCD, at 25 normalized collision energy) of the 10 most dominant ions (> 1 charges) selected from the first MS scan. The AGC settings were 3×10^6 for the full MS and 1×10^5 for the MS/MS scans. A dynamic exclusion list was enabled with an exclusion duration of 20 s. The mass spectrometry data were analyzed using the MaxQuant software 1.5.2.8 for peak picking and identification using the Andromeda search engine, vs. the Saccharomyces cerevisiae section of the uniport database. Oxidation on methionine and acetylation on the N-terminus were accepted as variable modifications, and carbamidomethyl on cysteine was accepted as static modifications. Minimal peptide length was set to six amino acids and a maximum of three miscleavages was allowed. Peptide- and protein-level false discovery rates (FDRs) were filtered to 1% using the target-decoy strategy. Protein tables were filtered to eliminate the identifications from the reverse database and from common contaminants. The data were quantified by label-free analysis using the same software, based on extracted ion currents (XICs) of peptides enabling quantitation from each LC/MS run for each peptide identified in any of the experiments. Annotation and statistical analysis were done using the Perseus and the String software (https://string-db.org/).

Filtering and normalization of LC/MS/MS results

In order to establish a list of identified mitochondrial proteins on the project, LC/MS/MS results from three different repeats were filtered by comparing the results to three sources of yeast mitochondrial annotation: SGD, Uniprot, and Go-annotation analysis of cellular compartment by using STRING gene ontology tools. Protein table was also filtered to eliminate single peptide identifications. Following this filtering, we keep analyzing only the mitochondrial proteins. We calculate the intensity relative percentage of each protein, from the total intensity of all mitochondrial proteins.

ImageStream analysis

The ImageStreamX system is an advanced imaging flow cytometer, combining features of fluorescent microscopy and flow cytometry. Cells in suspension pass through the instrument in a single file where transmitted light, scattered light, and emitted fluorescence are collected, at a rate of up to 5,000 cells s^{-1}. This is accompanied by a dedicated image analysis software (IDEAS), which allows advanced quantification of intensity, location, morphology, population statistics, and more, within tens of thousands of cells per sample. It allows analysis of rare subpopulations in highly heterogenous samples and gives rise to novel applications that were difficult to achieve by either conventional flow cytometry or microscopy (Zuba-Surma *et al*, 2007).

Imaging flow cytometry

Vesicles were stained as described above and imaged by ImageStream mark II (Amnis, Part of Luminex, Au. TX). At least 3×10^4 events were collected from each sample. Lasers used were 488 nm (400 mw), 561 nm (200 mw), and 785 nm (5 mw). Channels acquired were 2 (GFP), 4 (TMRE), 6 (Side scatter), and 1 + 9 for the bright-field image. Inter-channel spillover was compensated by acquiring single-stained controls. Images were analyzed using

 © 2023 Weizmann Institute of Science

IDEAS 6.3 software (Amnis, Part of Luminex, Au. TX). Debris and aggregates were removed by plotting the bright-field area (in square microns) vs. the side scatter intensity (The light scattered 90 degrees of the sample). Only objects with low bright field and low side scatter were taken for the analysis. Positive events for GFP and TMRE were gated by plotting the Max Pixel values (the largest value of the background-subtracted pixels contained in the input mask) of the GFP and TMRE staining. The gate was set according to the single-stained controls.

ATP production and complementation

The ATP production assay was adapted to Yeast and HEK293 cell MDVs based on our previously described work for mitochondria (Shufaro *et al*, 2012). Briefly, 10^{11} MDVs/ml (stored at −20°C and thawed immediately before the assay) were incubated for 15 min at 30°C for yeast MDVs or 37°C for cell MDVs in assay buffer (5 mM K_2HPO_4, 10 mM, 100 mM KCl, 5 mM $MgCl_2$, 0.005 mM EDTA, 75 mM mannitol, 25 mM sucrose, and 0.6 mg/mg fatty acid-free bovine serum albumin, at pH 7.4) in the presence or absence of 0.6 mM ADP, 40 mM pyruvate, 20 mM malate, 11 mM succinate, 4 μM cyanide 3-chlorophenylhydrazone (CCCP), 1.5 μM oligomycin (Sigma-Aldrich-Merck, Darmstadt, Germany). Subsequently, relative ATP levels were determined by luciferin-luciferase ATPlite luminescence assay system (Perkin Elmer, Akron, Ohio, USA) according to the manufacturer's instructions. Luminescence measurements were performed with a Synergy HT microplate reader (Bio-Tek Instruments, Vinooski, VT, USA). For complementation assays mitochondria (10 μg/μl) were preincubated with MDVs for 30 min in assay buffer at 30°C, then washed twice in large volumes of SH buffer (0.6 M sorbitol, 20 Mm Hepes pH 7.4) following centrifugation at 18,000 *g* for 20 min at 4°C. The mitochondria were subsequently resuspended in the original volume and incubated in the presence or absence of ADP for 15 min before assaying ATP.

Statistical analysis

All statistical analyses were performed using the GraphPad Prism (version 8.0) statistical analysis program. If not indicated otherwise, all the *P*-values in the figures measured between the indicated samples were quantified using the unpaired two-tailed Student's *t*-test. Data distribution was assumed to be normal, but this was not formally tested. The significance of the mean comparison is present in each figure.

Data availability

The datasets generated during this study are available from the corresponding authors upon reasonable request. The mass spectrometry proteomics data have been deposited to the ProteomeXchange Consortium via the PRIDE [1] partner repository with the dataset identifier PXD039191 and PXD039192 (http://www.ebi.ac.uk/pride/archive/projects/PXD039191 and http://www.ebi.ac.uk/pride/archive/projects/PXD035192).

Expanded View for this article is available online.

Acknowledgements

We thank Alexander Tzagoloff (Columbia University, USA) for generously providing antisera, Yael Freidman (Hebrew University, Israel) for performing Electron Microscopy Images, and Yoav Shaul for critical reading of the manuscript and data processing. Illustration was generated by the BioRender software (https://app.biorender.com/). OP is grateful for the support from the Israel Science Foundation (ISF; Grant Application no. 1445/17) and the German Israel Project (DIP; Grant no. P17516. The research of NR-R is supported by the Benoziyo Endowment Fund for the Advancement of Science, the Jeanne and Joseph Nissim Foundation for Life Sciences Research, the Dr. Barry Sherman Institute for Medical Chemistry and the Samuel M. Soref and Helene K. Soref Foundation. NR-R is the incumbent of the Enid Barden and Aaron J. Jade President's Development Chair for New Scientists in Memory of Cantor John Y. Jade. NR-R is grateful for the support from the European Research Council (ERC) under the European Union's Horizon 2020 research and innovation program (grant agreement No. 757743), the Minerva Program support (grant number 714142), and the Israel Science Foundation (ISF; Grant Application no. 570/21).

Author contributions

Reut Hazan (Ben-Menachem): Conceptualization; investigation; methodology; writing – original draft; project administration; writing – review and editing. **Dvora Lintzer:** Conceptualization; validation; investigation; methodology; writing – original draft. **Tamar Ziv:** Resources; data curation; software; methodology. **Koyeli Das:** Investigation. **Irit Rosenhek-Goldian:** Software; methodology. **Ziv Porat:** Data curation; software; methodology. **Hila Ben Ami Pilo:** Methodology. **Sharon Karniely:** Conceptualization. **Ann Saada:** Conceptualization; supervision; investigation; methodology; writing – original draft; writing – review and editing. **Neta Regev-Rudzki:** Conceptualization; resources; supervision; project administration; writing – review and editing. **Ophry Pines:** Conceptualization; resources; supervision; writing – original draft; project administration; writing – review and editing.

Disclosure and competing interests statement

The authors declare that they have no conflict of interest.

References

Abels ER, Breakefield XO (2016) Introduction to extracellular vesicles: biogenesis, RNA cargo selection, content, release, and uptake. *Cell Mol Neurobiol* 36: 301 – 312

Andrade-Navarro MA, Sanchez-Pulido L, McBride HM (2009) Mitochondrial vesicles: an ancient process providing new links to peroxisomes. *Curr Opin Cell Biol* 21: 560 – 567

Balaban RS, Nemoto S, Finkel T (2005) Mitochondria, oxidants, and aging. *Cell* 120: 483 – 495

Ben-Menachem R, Tal M, Shadur T, Pines O (2011) A third of the yeast mitochondrial proteome is dual localized: a question of evolution. *Proteomics* 11: 4468 – 4476

Cavelier C, Ohnsorg PM, Rohrer L, von Eckardstein A (2012) The beta-chain of cell surface F(0)F(1) ATPase modulates apoA-I and HDL transcytosis through aortic endothelial cells. *Arterioscler Thromb Vasc Biol* 32: 131 – 139

Cunningham KW, Wickner WT (1989) Yeast KEX2 protease and mannosyltransferase I are localized to distinct compartments of the secretory pathway. *Yeast* 5: 25 – 33

© 2023 Weizmann Institute of Science

D'Acunzo P, Kim Y, Ungania JM, Perez-Gonzalez R, Goulbourne CN, Levy E (2022) Isolation of mitochondria-derived mitovesicles and subpopulations of microvesicles and exosomes from brain tissues. *Nat Protoc* 17: 2517 – 2549

Daum G, Bohni PC, Schatz G (1982) Import of proteins into mitochondria. Cytochrome b2 and cytochrome c peroxidase are located in the intermembrane space of yeast mitochondria. *J Biol Chem* 257: 13028 – 13033

Dekel E, Yaffe D, Rosenhek-Goldian I, Ben-Nissan G, Ofir-Birin Y, Morandi MI, Ziv T, Sisquella X, Pimentel MA, Nebl T *et al* (2021) 20S proteasomes secreted by the malaria parasite promote its growth. *Nat Commun* 12: 1172

Gezsi A, Kovacs A, Visnovitz T, Buzas EI (2019) Systems biology approaches to investigating the roles of extracellular vesicles in human diseases. *Exp Mol Med* 51: 1 – 11

Grevel A, Pfanner N, Becker T (2019) Coupling of import and assembly pathways in mitochondrial protein biogenesis. *Biol Chem* 401: 117 – 129

Hernandez-Barranco A, Nogues L, Peinado H (2021) Could extracellular vesicles contribute to generation or awakening of "sleepy" metastatic niches? *Front Cell Dev Biol* 9: 625221

Jiang X, Wang X (2004) Cytochrome C-mediated apoptosis. *Annu Rev Biochem* 73: 87 – 106

Kalderon B, Pines O (2014) Protein folding as a driving force for dual protein targeting in eukaryotes. *Front Mol Biosci* 1: 23

Kisslov I, Naamati A, Shakarchy N, Pines O (2014) Dual-targeted proteins tend to be more evolutionarily conserved. *Mol Biol Evol* 31: 2770 – 2779

Konig T, Nolte H, Aaltonen MJ, Tatsuta T, Krols M, Stroh T, Langer T, McBride HM (2021) MIROs and DRP1 drive mitochondrial-derived vesicle biogenesis and promote quality control. *Nat Cell Biol* 23: 1271 – 1286

Matheoud D, Sugiura A, Bellemare-Pelletier A, Laplante A, Rondeau C, Chemali M, Fazel A, Bergeron JJ, Trudeau LE, Burelle Y *et al* (2016) Parkinson's disease-related proteins PINK1 and Parkin repress mitochondrial antigen presentation. *Cell* 166: 314 – 327

McLelland GL, Soubannier V, Chen CX, McBride HM, Fon EA (2014) Parkin and PINK1 function in a vesicular trafficking pathway regulating mitochondrial quality control. *EMBO J* 33: 282 – 295

Meningher T, Barsheshet Y, Ofir-Birin Y, Gold D, Brant B, Dekel E, Sidi Y, Schwartz E, Regev-Rudzki N, Avni O *et al* (2020) Schistosomal extracellular vesicle-enclosed miRNAs modulate host T helper cell differentiation. *EMBO Rep* 21: e47882

Morgenstern M, Stiller SB, Lubbert P, Peikert CD, Dannenmaier S, Drepper F, Weill U, Hoss P, Feuerstein R, Gebert M *et al* (2017) Definition of a high-confidence mitochondrial proteome at quantitative scale. *Cell Rep* 19: 2836 – 2852

Nečas D, Klapetek P (2012) Gwyddion: an open-source software for SPM data analysis. *Open Physics* 10: 181 – 188

Neupert W, Herrmann JM (2007) Translocation of proteins into mitochondria. *Annu Rev Biochem* 76: 723 – 749

Neuspiel M, Schauss AC, Braschi E, Zunino R, Rippstein P, Rachubinski RA, Andrade-Navarro MA, McBride HM (2008) Cargo-selected transport from the mitochondria to peroxisomes is mediated by vesicular carriers. *Curr Biol* 18: 102 – 108

Ofir-Birin Y, Heidenreich M, Regev-Rudzki N (2017) Pathogen-derived extracellular vesicles coordinate social behaviour and host manipulation. *Semin Cell Dev Biol* 67: 83 – 90

Picca A, Guerra F, Calvani R, Romano R, Coelho-Junior HJ, Bucci C, Leeuwenburgh C, Marzetti E (2022) Mitochondrial-derived vesicles in skeletal muscle remodeling and adaptation. *Semin Cell Dev Biol* https://doi.org/10.1016/j.semcdb.2022.03.023

Regev-Rudzki N, Wilson DW, Carvalho TG, Sisquella X, Coleman BM, Rug M, Bursac D, Angrisano F, Gee M, Hill AF *et al* (2013) Cell-cell communication between malaria-infected red blood cells via exosome-like vesicles. *Cell* 153: 1120 – 1133

Reichert AS, Neupert W (2004) Mitochondriomics or what makes us breathe. *Trends Genet* 20: 555 – 562

Rizzuto R, Pinton P, Carrington W, Fay FS, Fogarty KE, Lifshitz LM, Tuft RA, Pozzan T (1998) Close contacts with the endoplasmic reticulum as determinants of mitochondrial Ca^{2+} responses. *Science* 280: 1763 – 1766

Rodrigues ML, Nimrichter L, Oliveira DL, Frases S, Miranda K, Zaragoza O, Alvarez M, Nakouzi A, Feldmesser M, Casadevall A (2007) Vesicular polysaccharide export in *Cryptococcus neoformans* is a eukaryotic solution to the problem of fungal trans-cell wall transport. *Eukaryot Cell* 6: 48 – 59

Ryan TA, Phillips EO, Collier CL, Jb Robinson A, Routledge D, Wood RE, Assar EA, Tumbarello DA (2020) Tollip coordinates Parkin-dependent trafficking of mitochondrial-derived vesicles. *EMBO J* 39: e102539

Schneider A (2020) Evolution of mitochondrial protein import – lessons from trypanosomes. *Biol Chem* 401: 663 – 676

Scorrano L, De Matteis MA, Emr S, Giordano F, Hajnoczky G, Kornmann B, Lackner LL, Levine TP, Pellegrini L, Reinisch K *et al* (2019) Coming together to define membrane contact sites. *Nat Commun* 10: 1287

Shufaro Y, Lebovich M, Aizenman E, Miller C, Simon A, Laufer N, Saada A (2012) Human granulosa luteal cell oxidative phosphorylation function is not affected by age or ovarian response. *Fertil Steril* 98: 166 – 172

Sisquella X, Ofir-Birin Y, Pimentel MA, Cheng L, Abou Karam P, Sampaio NG, Penington JS, Connolly D, Giladi T, Scicluna BJ *et al* (2017) Malaria parasite DNA-harbouring vesicles activate cytosolic immune sensors. *Nat Commun* 8: 1985

Song J, Pfanner N, Becker T (2018) Assembling the mitochondrial ATP synthase. *Proc Natl Acad Sci U S A* 115: 2850 – 2852

Soubannier V, McLelland GL, Zunino R, Braschi E, Rippstein P, Fon EA, McBride HM (2012a) A vesicular transport pathway shuttles cargo from mitochondria to lysosomes. *Curr Biol* 22: 135 – 141

Soubannier V, Rippstein P, Kaufman BA, Shoubridge EA, McBride HM (2012b) Reconstitution of mitochondria derived vesicle formation demonstrates selective enrichment of oxidized cargo. *PLoS One* 7: e52830

Todkar K, Chikhi L, Desjardins V, El-Mortada F, Pepin G, Germain M (2021) Selective packaging of mitochondrial proteins into extracellular vesicles prevents the release of mitochondrial DAMPs. *Nat Commun* 12: 1971

Trotter PJ, Voelker DR (1995) Identification of a non-mitochondrial phosphatidylserine decarboxylase activity (PSD2) in the yeast Saccharomyces cerevisiae. *J Biol Chem* 270: 6062 – 6070

Yogev O, Pines O (2011) Dual targeting of mitochondrial proteins: mechanism, regulation and function. *Biochim Biophys Acta* 1808: 1012 – 1020

Zhang AS, Sheftel AD, Ponka P (2005) Intracellular kinetics of iron in reticulocytes: evidence for endosome involvement in iron targeting to mitochondria. *Blood* 105: 368 – 375

Zuba-Surma EK, Kucia M, Abdel-Latif A, Lillard JW Jr, Ratajczak MZ (2007) The ImageStream system: a key step to a new era in imaging. *Folia Histochem Cytobiol* 45: 279 – 290

License: This is an open access article under the terms of the Creative Commons Attribution License, which permits use, distribution and reproduction in any medium, provided the original work is properly cited.

Expanded View Figures

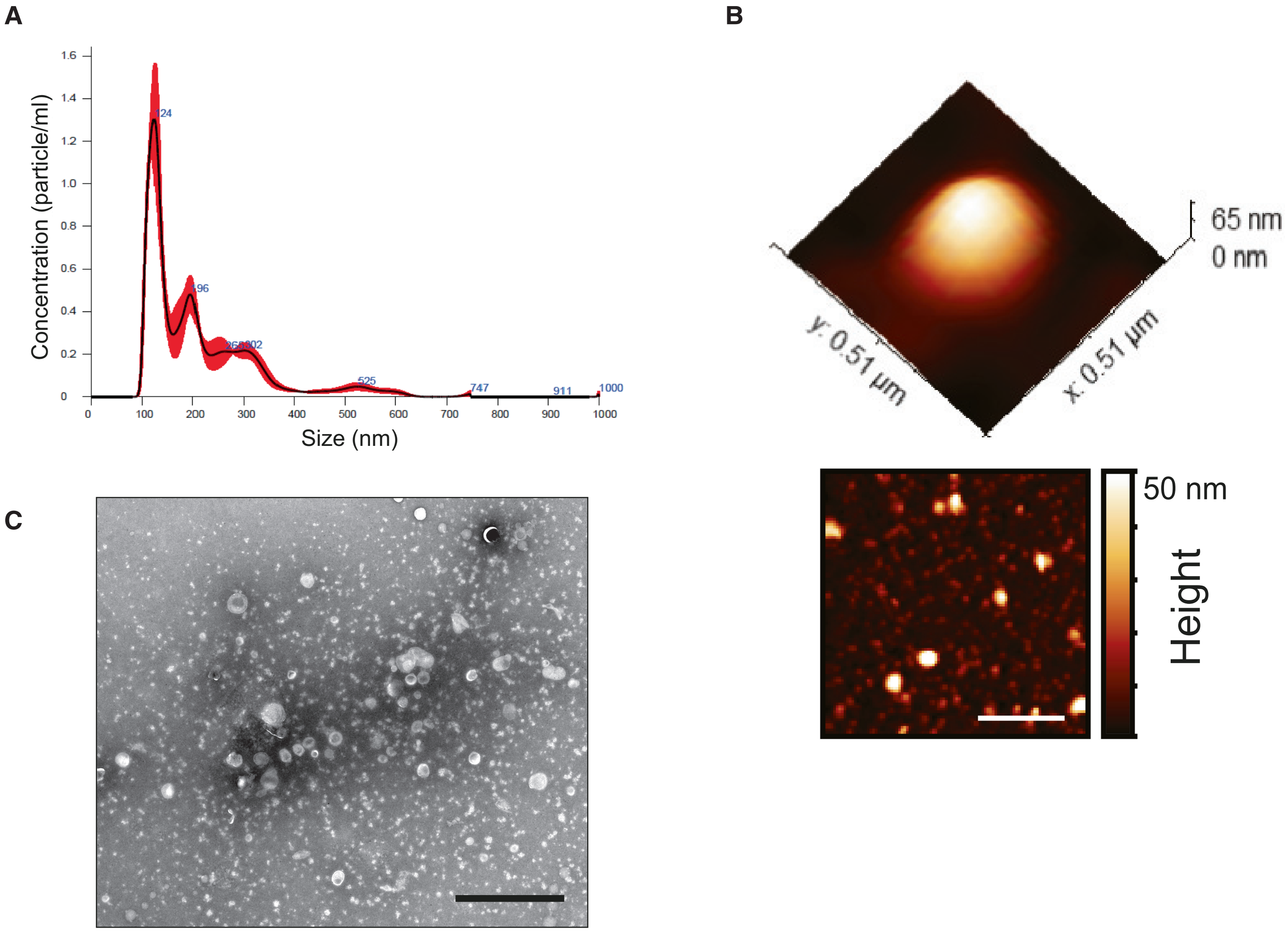

Figure EV1. Characterization of MDVs derived from Dnm1 KO mitochondria.

A Nanoparticle tracking analysis. Vesicle size distribution and concentration were performed using nanoparticle tracking analysis (NTA; Malvern Instruments, Nanosight NS300). Sample size distributions were calibrated in a liquid suspension by the analysis of Brownian motion via light scattering. Nanosight provides single particle size and concentration measurements.

B Atomic force microscopy. Representative AFM image and a 3D AFM image of one representative vesicle (Dnm1 KO), adsorbed on a mica modified with Mg^{2+} and imaged under PBS.

C TEM images of MDVs. Samples were stained with 2.0% uranyl acetate or 2.0% phosphotungstic acid. 5 µl of vesicles was placed on Formvar/carbon-coated copper 200 mesh grids (EMS), mixed with 5 µl of PTA for 10–20 s, while excess stain was blotted off and grids were dried. Samples were examined with Jeol (Jem-1400 Plus) transmission electron microscope. Scale bar—2 µm.

Source data are available online for this figure.

© 2023 Weizmann Institute of Science

Figure EV2. Vesicle characterization.

A Vesicle concentrations according to nanoparticle tracking analysis over time. Vesicle concentration was measured at the indicated time points, n = two repeats.

B Mitochondria ATP levels with time. Isolated mitochondria from wild-type cells were incubated for 0, 4 h, and 24 h at 30°C prior to measuring ATP production, in the absence or presence of succinate, by luciferin-luciferase luminometry as described in the methods section. Each value represents the mean ± SD for $n = 3$ technical repeats. Significant differences were detected using t-test, P-value = 1.9558E-05/1.03993E-06 for 24 and 4 h mitochondria with succinate, respectively.

C Western blot analysis of isolated MDVs. MDVs were purified from wild-type or KO-isolated mitochondria as mentioned, and the presence of Mdh1p and Por1p was assessed by western blot analysis using Mdh1 antisera.

D Vesicles count in WT and ΔDnm1 strains. MDVs isolated from Wild-type and Dnm1 KO strains were analyzed using nanoparticle tracking analysis (NTA; Malvern Instruments, Nanosight NS300). n = two repeats.

Source data are available online for this figure.

 © 2023 Weizmann Institute of Science

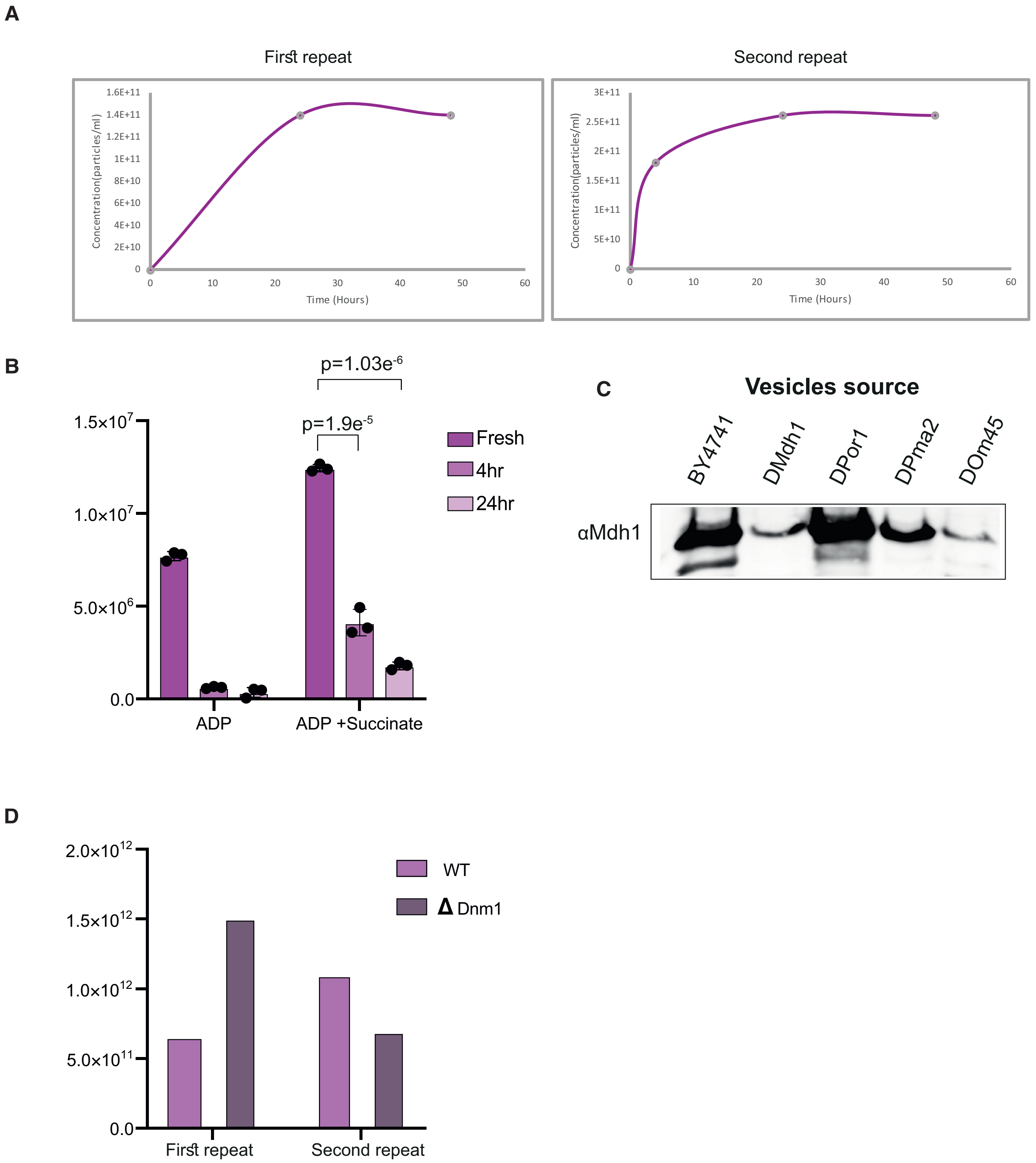
A
First repeat
Concentration(particles/ml)
1.6E+11
1.4E+11
1.2E+11
1E+11
8E+10
6E+10
4E+10
2E+10
0
0 10 20 30 40 50 60
Time (Hours)
Second repeat
Concentration(particles/ml)
3E+11
2.5E+11
2E+11
1.5E+11
1E+11
5E+10
0
0 10 20 30 40 50 60
Time (Hours)
B
p=1.03e-6
p=1.9e-5
1.5x10^7
1.0x10^7
5.0x10^6
0.0
Fresh
4hr
24hr
ADP
ADP +Succinate
C
Vesicles source
BY4741
DMdh1
DPor1
DPma2
DOm45
αMdh1
D
2.0x10^12
1.5x10^12
1.0x10^12
5.0x10^11
0.0
WT
Δ Dnm1
First repeat
Second repeat

Figure EV2.

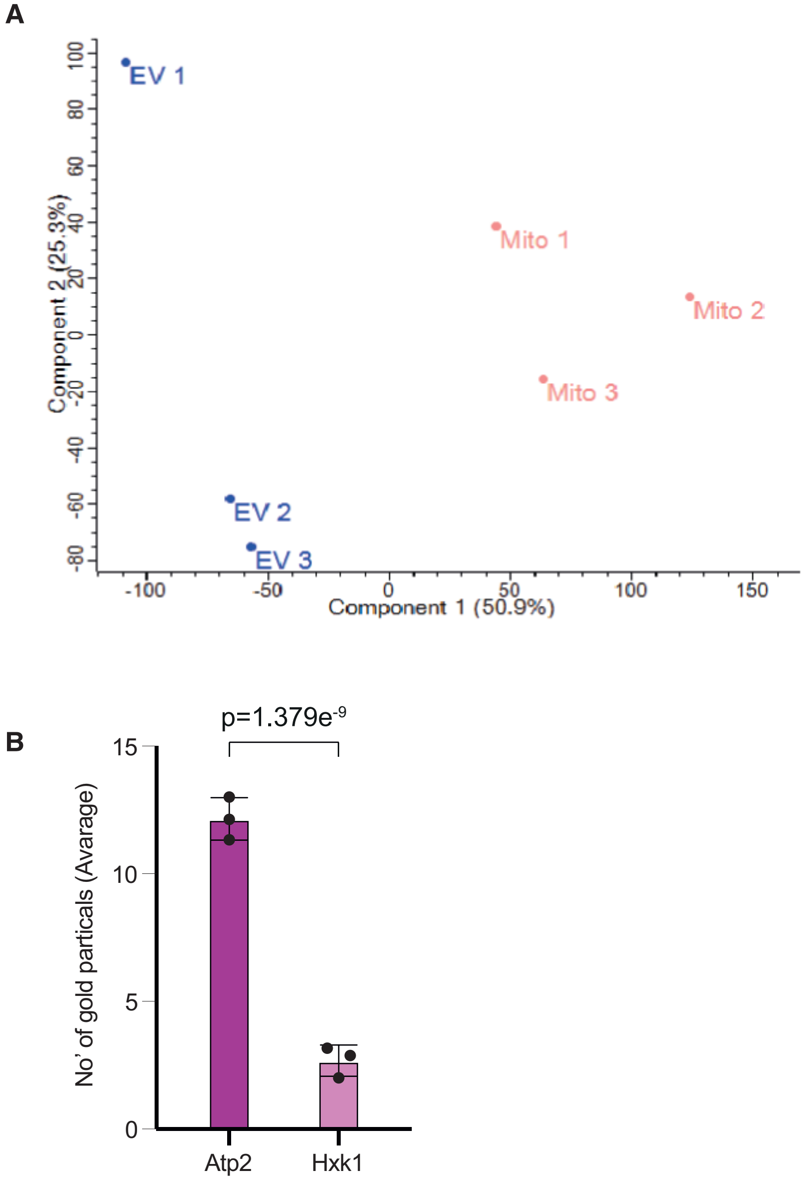

Figure EV3. Data for MDVs from yeast wild-type mitochondria.

A Principal component analysis (PCA) visualizes the projection of the dataset defined by PCA in 2-dimensional viewers, transforming the large set of protein information into a protein profile of each sample. The analysis was done by Perseus software.

B The average number of gold particles of Atp2 is significantly higher compared with the controls. The number of gold particles was measured using ImageJ software (ImageJ.nih.gov). Each value represents the mean ± SD for $n = 3$ biological repeats. Significant differences were detected using *t*-test, *P*-value = 1.379E-09.

Source data are available online for this figure.

 © 2023 Weizmann Institute of Science

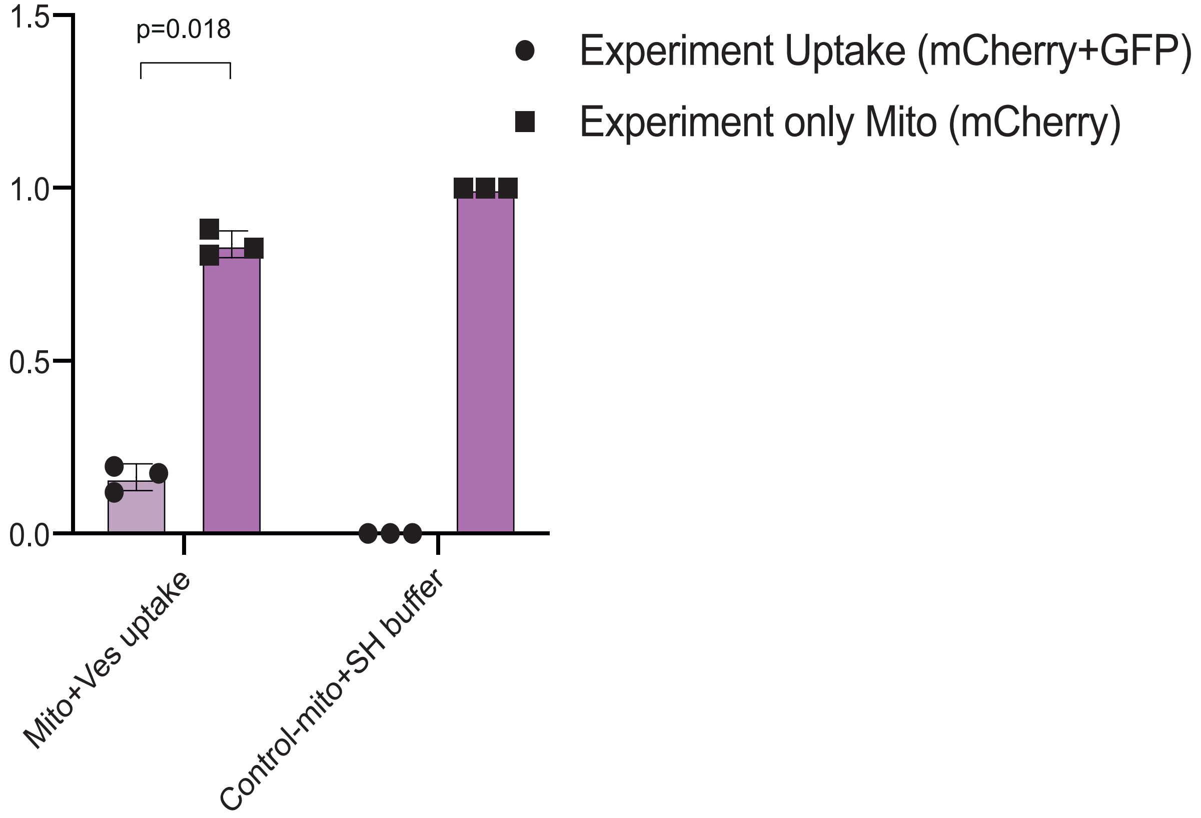

Figure EV4. Co-localization of mitochondria and MDVs as visualized by imaging flow cytometry.

mCherry-labeled mitochondria were preincubated in the presence or absence of GFP-labeled MDVs, washed, and imaged by ImageStreamX mark II (Amnis, Part of Luminex, Au. TX). Each value represents the mean ± SD for $n = 3$ biological repeats. Significant differences were detected using t-test, P-value = 0.018. Mito, mitochondria; Ves, MDVs.

Source data are available online for this figure.

© 2023 Weizmann Institute of Science

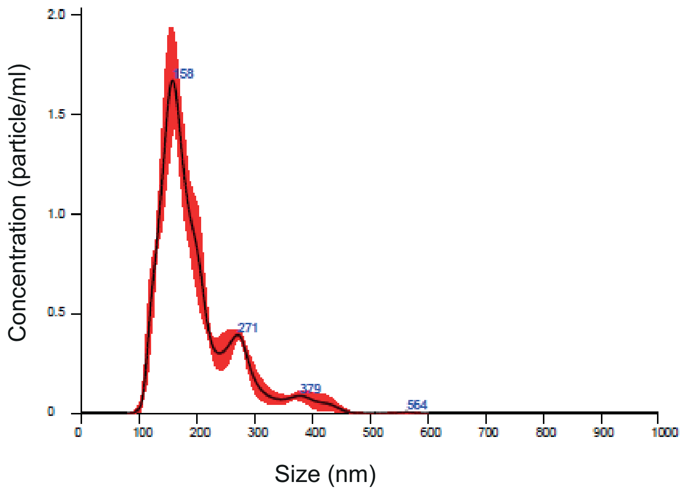

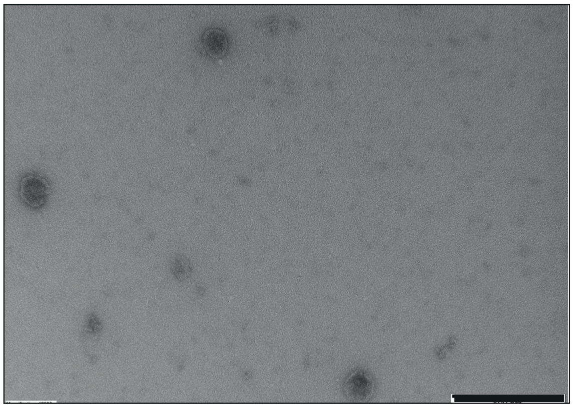

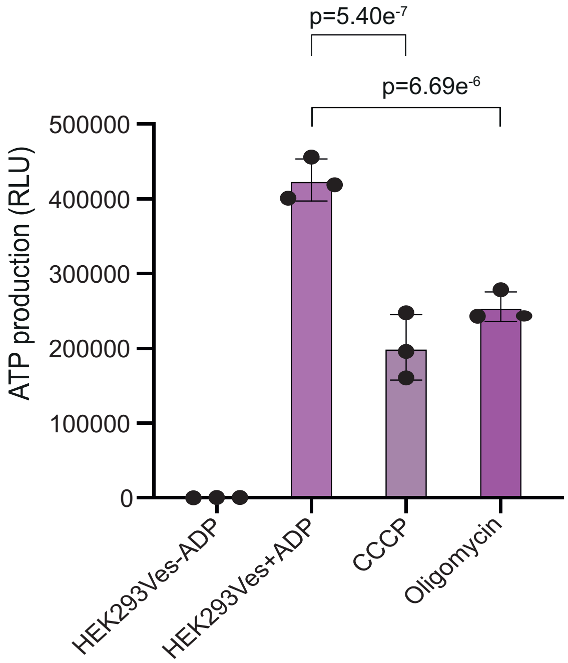

Figure EV5. MDVs derived from HEK293-isolated mitochondria.

A Nanoparticle tracking analysis of HEK293-derived MDVs. Vesicles derived from HEK293-isolated mitochondria were subjected to size distribution and concentration using nanoparticle tracking analysis (NTA).

B TEM images of HEK293-derived MDVs. Samples were stained with 2.0% uranyl acetate or 2.0% phosphotungstic acid. 5 μl of vesicles was placed on Formvar/carbon-coated copper 200 mesh grids (EMS), mixed with 5 μl of PTA for 10–20 s, while excess stain was blotted off and grids were dried. Samples were examined with Jeol (Jem-1400 Plus) transmission electron microscope. Scale bar—500 nm.

C HEK293-derived MDVs, produce ATP in an enzymatic reaction dependent on membrane potential. Mitochondrial-derived vesicles from HEK293-isolated mitochondria were incubated in the presence or absence of ADP, CCCP, or oligomycin for 15 min at 37°C. Subsequently, ATP was measured by luciferin-luciferase luminometry. Each value represents the mean ± SD for $n = 3$ biological repeats. Significant differences were detected using *t*-test, *P*-value = 6.69E-06/5.40E-07 for oligomycin and CCCP, respectively.

Source data are available online for this figure.

 © 2023 Weizmann Institute of Science
