# Supplementary figures and images for: Mitochondrial‐derived vesicles retain membrane potential and contain a functional ATP synthase
Source: EMBO Rep. 2023 Mar 17;24(5):e56114. doi: 10.15252/embr.202256114 (PMC10157309; doi:10.15252/embr.202256114)

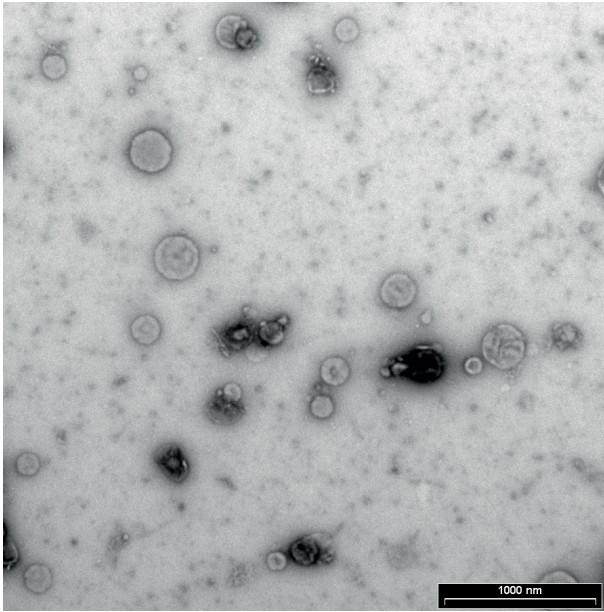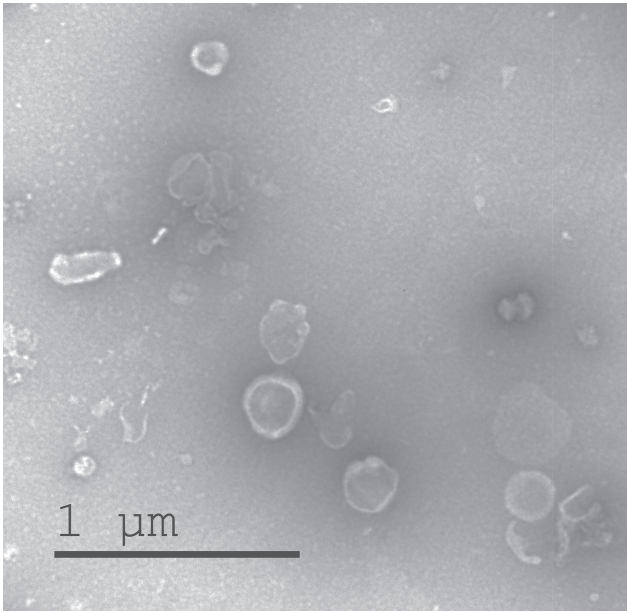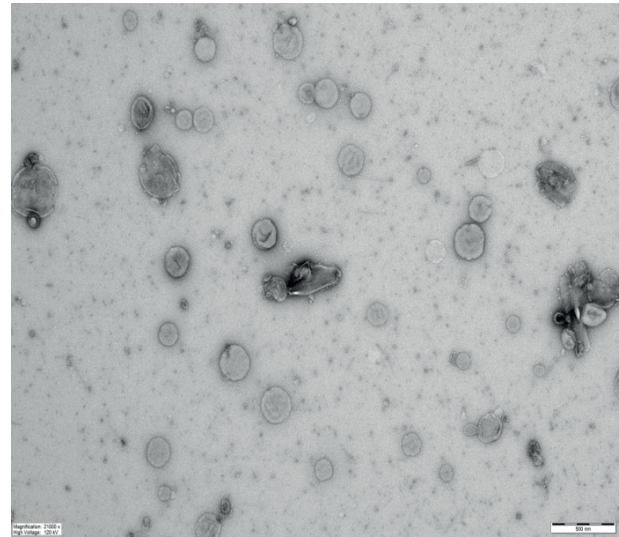

Supplement: Supplementary file 10 — Source Data for Figure 2 [file EMBR-24-e56114-s008.zip › Fig2C TEM vesicles.pdf]

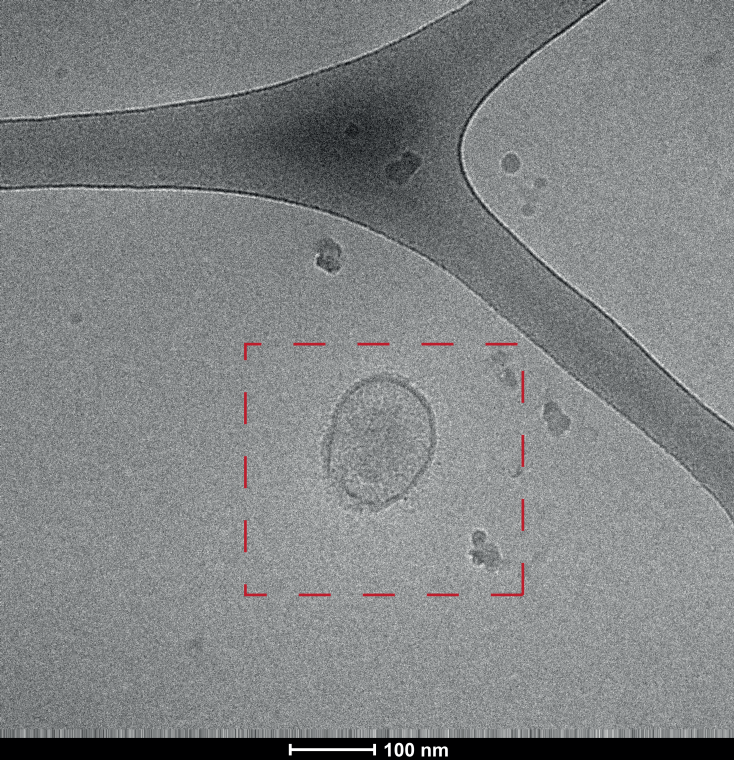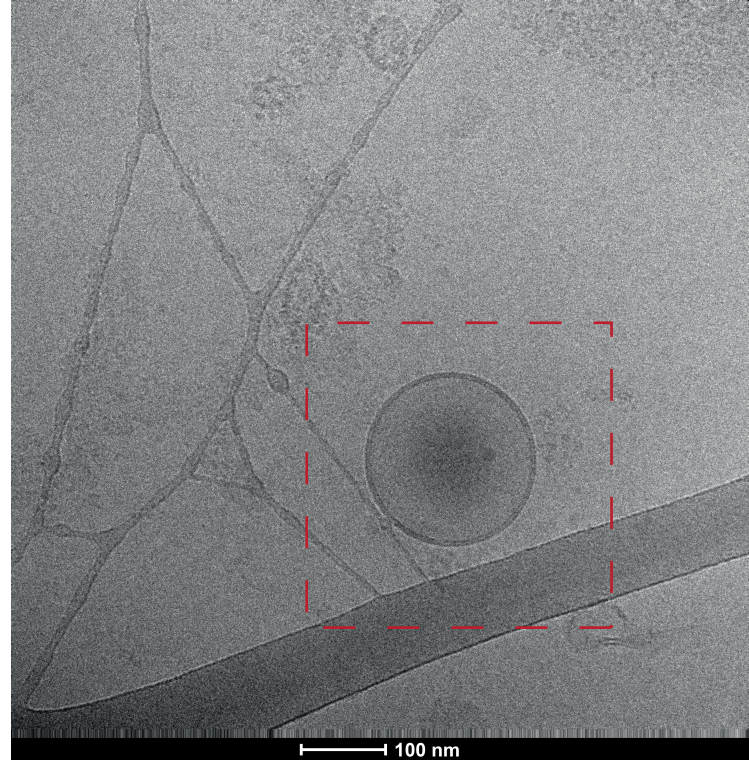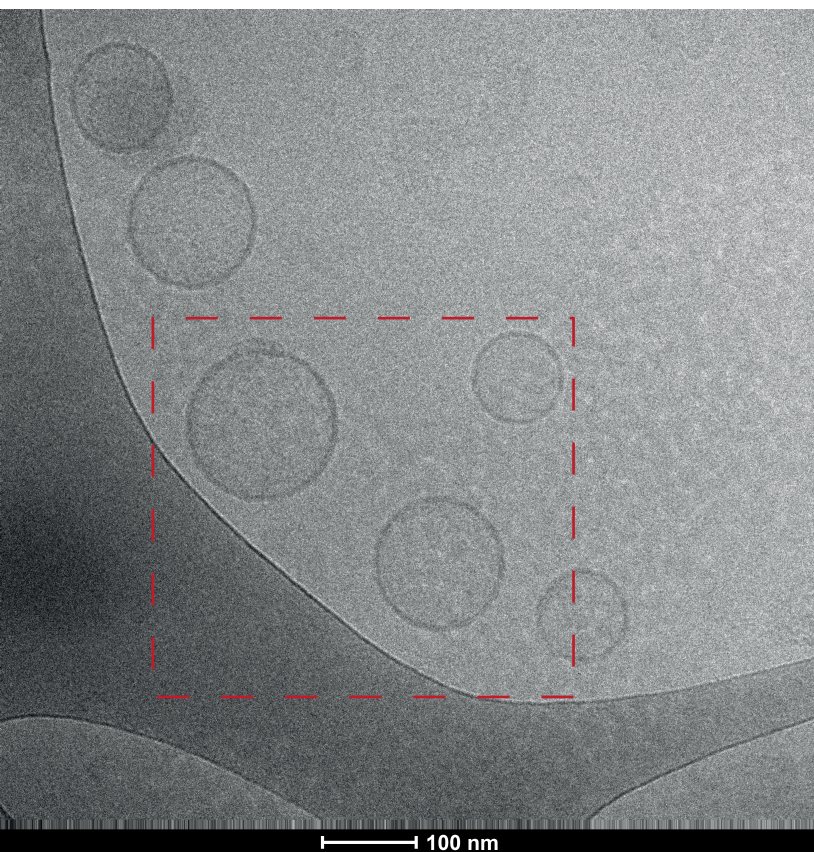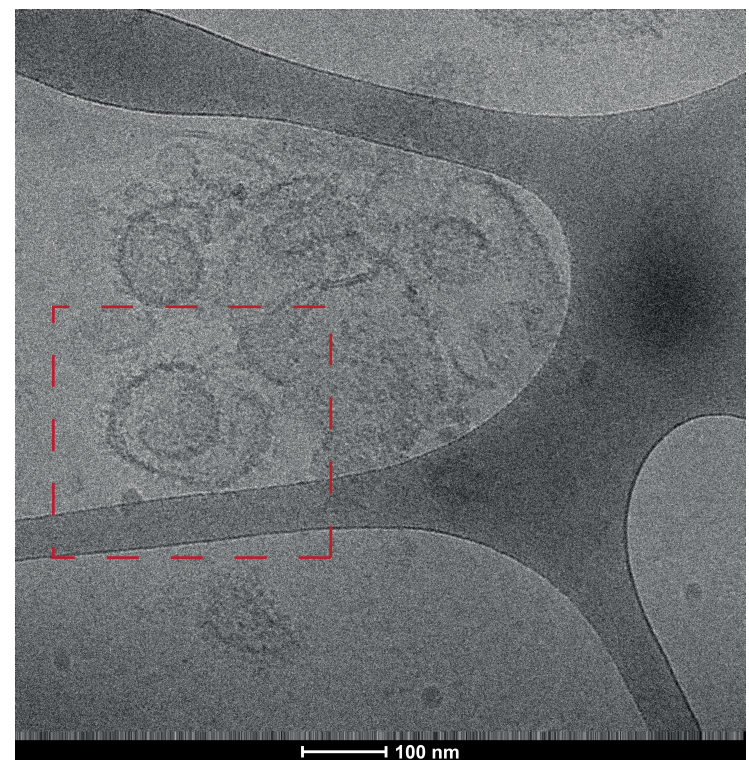

Supplement: Supplementary file 10 — Source Data for Figure 2 [file EMBR-24-e56114-s008.zip › FIG2D cryo-EM.pdf]
