# Supplementary material for: Mitochondrial‐derived vesicles retain membrane potential and contain a functional ATP synthase
Source: EMBO Rep. 2023 Mar 17;24(5):e56114. doi: 10.15252/embr.202256114 (PMC10157309; doi:10.15252/embr.202256114)
Supplement: Supplementary file 11 — Source Data for Figure 3 [file EMBR-24-e56114-s003.zip › Fig3D numerical values.pdf]

Numerical values of % of representative mitochondrial proteins (Figure 3D)

	EV	Mito
**Mitochondrial inner membrane**	8.7203E+11	1.68823E+12
**Mitochondrial outer membrane**	4.24089E+11	2.42232E+11
**Mitochondrial ribosomal proeins**	34794649503	89482433543
**Enzymes**	38206108101	85195115307
**ATPases**	9.5223E+11	1.85266E+12
**others**	1.64109E+12	1.80114E+12
