# Supplementary material for: Mitochondrial‐derived vesicles retain membrane potential and contain a functional ATP synthase
Source: EMBO Rep. 2023 Mar 17;24(5):e56114. doi: 10.15252/embr.202256114 (PMC10157309; doi:10.15252/embr.202256114)
Supplement: Supplementary file 14 [file EMBR-24-e56114-s009.pdf]

EMBO reports

Mitochondrial–derived Vesicles retain membrane potential and contain a functional ATP synthase

Reut Hazan (Ben-Menachem), Dvora Lintzer, Tamar Ziv, Koyeli Das, Irit Rosenhek-Goldian, Ziv Porat, Hila Ben Ami Pilo, Sharon Karniely, Ann Saada, Neta Regev-Rudzki, and Ophry Pines
DOI: 10.15252/embr.202256114

Corresponding author(s): Neta Regev-Rudzki (neta.regev-rudzki@weizmann.ac.il) , Ophry Pines (ophryp@ekmd.huji.ac.il), Ann Saada (Annsr@hadassah.org.il)

Review Timeline:

Submission Date:	12th Sep 22
Editorial Decision:	5th Oct 22
Revision Received:	3rd Jan 23
Editorial Decision:	26th Jan 23
Revision Received:	6th Feb 23
Editorial Decision:	15th Feb 23
Revision Received:	27th Feb 23
Accepted:	28th Feb 23

Editor: Achim Breiling

Transaction Report:

(Note: With the exception of the correction of typographical or spelling errors that could be a source of ambiguity, letters and reports are not edited. Depending on transfer agreements, referee reports obtained elsewhere may or may not be included in this compilation. Referee reports are anonymous unless the Referee chooses to sign their reports.)

Dear Dr. Regev-Rudzki,

Thank you for the submission of your research manuscript to EMBO reports. I have now received the reports from the three referees that were asked to evaluate your study, which can be found at the end of this email.

As you will see, the referees think that these findings are of high interest. However, they have several comments, concerns, and suggestions, indicating that a major revision of the manuscript is necessary to allow publication of the study in EMBO reports. As the reports are below, and all the referee concerns need to be addressed, I will not detail them here.

Given the constructive referee comments, we would like to invite you to revise your manuscript with the understanding that all referee concerns must be addressed in the revised manuscript or in a detailed point-by-point response. Acceptance of your manuscript will depend on a positive outcome of a second round of review. It is EMBO reports policy to allow a single round of revision only and acceptance of the manuscript will therefore depend on the completeness of your responses included in the next, final version of the manuscript.

Revised manuscripts should be submitted within three months of a request for revision. Please contact me to discuss the revision (also by video chat) if you have questions or comments regarding the revision, or should you need additional time.

When submitting your revised manuscript, please also carefully review the instructions that follow below.

PLEASE NOTE THAT upon resubmission revised manuscripts are subjected to an initial quality control prior to exposition to re-review. Upon failure in the initial quality control, the manuscripts are sent back to the authors, which may lead to delays. Frequent reasons for such a failure are the lack of the data availability section (please see below) and the presence of statistics based on n=2 (the authors are then asked to present scatter plots or provide more data points).

When submitting your revised manuscript, we will require:

1) a .docx formatted version of the final manuscript text (including legends for main figures, EV figures and tables), but without the figures included. Figure legends should be compiled at the end of the manuscript text.

2) individual production quality figure files as .eps, .tif, .jpg (one file per figure), of main figures (up to 8) and EV figures. Please upload these as separate, individual files upon re-submission.

The Expanded View format, which will be displayed in the main HTML of the paper in a collapsible format, has replaced the Supplementary information. You can submit up to 5 images as Expanded View. Please follow the nomenclature Figure EV1, Figure EV2 etc. The figure legend for these should be included in the main manuscript document file in a section called Expanded View Figure Legends after the main Figure Legends section. Additional Supplementary material should be supplied as a single pdf file labeled Appendix. The Appendix should have page numbers and needs to include a table of content on the first page (with page numbers) and legends for all content. Please follow the nomenclature Appendix Figure Sx, Appendix Table Sx etc. throughout the text, and also label the figures and tables according to this nomenclature.

For more details, please refer to our guide to authors:
http://www.embopress.org/page/journal/14693178/authorguide#manuscriptpreparation

Please consult our guide for figure preparation:
http://wol-prod-cdn.literatumonline.com/pb-assets/embo-site/EMBOPress_Figure_Guidelines_061115-1561436025777.pdf

See also the guidelines for figure legend preparation:
https://www.embopress.org/page/journal/14693178/authorguide#figureformat

3) a complete author checklist, which you can download from our author guidelines (https://www.embopress.org/page/journal/14693178/authorguide). Please insert page numbers in the checklist to indicate where the requested information can be found in the manuscript. The completed author checklist will also be part of the RPF.

Please also follow our guidelines for the use of living organisms, and the respective reporting guidelines:
http://www.embopress.org/page/journal/14693178/authorguide#livingorganisms

4) that primary datasets produced in this study (e.g. RNA-seq, ChIP-seq, structural and array data) are deposited in an appropriate public database. If no primary datasets have been deposited, please also state this in a dedicated section (e.g. 'No primary datasets have been generated and deposited'), see below.

See also: http://embor.embopress.org/authorguide#datadeposition

Please remember to provide a reviewer password if the datasets are not yet public.

The accession numbers and database should be listed in a formal "Data Availability" section (placed after Materials & Methods) that follows the model below. This is now mandatory (like the COI statement). Please note that the Data Availability Section is restricted to new primary data that are part of this study. This section is mandatory. As indicated above, if no primary datasets have been deposited, please state this in this section

Data availability

The datasets produced in this study are available in the following databases:

- RNA-Seq data: Gene Expression Omnibus GSE46843 (https://www.ncbi.nlm.nih.gov/geo/query/acc.cgi?acc=GSE46843)
- [data type]: [name of the resource] [accession number/identifier/doi] ([URL or identifiers.org/DATABASE:ACCESSION])

*** Note - All links should resolve to a page where the data can be accessed. ***

Moreover, I have these editorial requests:

6) We now request the publication of original source data with the aim of making primary data more accessible and transparent to the reader. Our source data coordinator will contact you to discuss which figure panels we would need source data for and will also provide you with helpful tips on how to upload and organize the files.

7) Our journal encourages inclusion of *data citations in the reference list* to directly cite datasets that were re-used and obtained from public databases. Data citations in the article text are distinct from normal bibliographical citations and should directly link to the database records from which the data can be accessed. In the main text, data citations are formatted as follows: "Data ref: Smith et al, 2001" or "Data ref: NCBI Sequence Read Archive PRJNA342805, 2017". In the Reference list, data citations must be labeled with "[DATASET]". A data reference must provide the database name, accession number/identifiers and a resolvable link to the landing page from which the data can be accessed at the end of the reference. Further instructions are available at: http://www.embopress.org/page/journal/14693178/authorguide#referencesformat

8) Regarding data quantification and statistics, please make sure that the number "n" for how many independent experiments were performed, their nature (biological versus technical replicates), the bars and error bars (e.g. SEM, SD) and the test used to calculate p-values is indicated in the respective figure legends (also for potential EV figures and all those in the final Appendix). Please also check that all the p-values are explained in the legend, and that these fit to those shown in the figure. Please provide statistical testing where applicable. Please avoid the phrase 'independent experiment', but clearly state if these were biological or technical replicates. Please also indicate (e.g. with n.s.) if testing was performed, but the differences are not significant. In case n=2, please show the data as separate datapoints without error bars and statistics. See also: http://www.embopress.org/page/journal/14693178/authorguide#statisticalanalysis

9) Please also note our reference format:
http://www.embopress.org/page/journal/14693178/authorguide#referencesformat

10) Please add scale bars of similar style and thickness to all the microscopic images, using clearly visible black or white bars (depending on the background). Please place these in the lower right corner of the images themselves. Please do not write on or near the bars in the image but define the size in the respective figure legend.

11) We updated our journal's competing interests policy in January 2022 and request authors to consider both actual and perceived competing interests. Please review the policy https://www.embopress.org/competing-interests and update your competing interests if necessary. Please name this section 'Disclosure and Competing Interests Statement' and put it after the Acknowledgements section.

12) We now use CRediT to specify the contributions of each author in the journal submission system. CRediT replaces the author contribution section. Please use the free text box to provide more detailed descriptions. See also guide to authors: https://www.embopress.org/page/journal/14693178/authorguide#authorshipguidelines

Please also add up to 5 key words to the title page, below the abstract.

I look forward to seeing a revised version of your manuscript when it is ready. Please let me know if you have questions or comments regarding the revision.

Please use this link to submit your revision: https://embor.msubmit.net/cgi-bin/main.plex

Yours sincerely,

Achim Breiling
Senior Editor
EMBO Reports

Referee #1:

The manuscript by Reut et al. Describes the properties of MDVs isolated from yeast mitochondria and their ability to produce ATP. This is potentially an important finding. However, while the initial characterization of the MDVs is good, the experiments related to the ATP synthase should be more thoroughly done.

Major points:
1. If Hxk1 is really a cytosolic protein, then the authors cannot claim that the mitochondrial fraction is free of cytosolic contamination since there are bands corresponding to Hxk1 in the mitochondrial fraction (Fig. 1A). However, as hexokinases are known to associate with mitochondria in some circumstances, this might not be the best control (this is also an issue for the immune-gold experiments). Other controls should be used. Similarly, the authors cannot claim that their mitochondrial preparations are pure based on only one protein evaluated.
2. While initial reports suggested that MDVs were DRP1-independent, a recent study showed that DRP1 is required for TOM20-positive MDVs (albeit at lower levels than for fission; 10.1038/s41556-021-00798-4). A more thorough comparison of WT and Dnm1 KO cells, including potential morphological differences (e.g. single vs double membrane) and yield is thus required.
3. It is unclear why the Mdh1 and Om45 strains were chosen. The observation that these strains produce less MDVs in vitro is interesting but would need to be confirmed by measuring MDV production in cells. Also, the authors should provide experimental evidence that the reduced MDV production in these strains is the result of a general mitochondrial defect, since this is what they claim.
4. It would be good to confirm the presence of more than one of the ATP synthase subunits (at least in HEK cells for which there are good antibodies). However, the main question that is not addressed here is whether components of the other ETC complexes are present and could explain the presence of a membrane potential. The presence of these proteins and the effect of ETC inhibition need to be assessed.
5. The small effect of cccp and oligomycin is worrisome and could suggest a mechanism other than the ATP synthase.
6. The ability of the MDVs to produce ATP is intriguing. How much ATP do these MDVs produce relative to mitochondria? Do they consume oxygen? The rescue experiment is also very interesting but lacks an MDV alone control. Also, the possibility that MDVs stay associated with mitochondria in the rescue experiment should be addressed. Is the ATP synthase actually transferred to recipient mitochondria?
6. MDVs with a membrane potential have been observed before (10.1016/j.cub.2011.11.057)
7. The manuscript seems to rely heavily on the Neuspiel paper. However, a number of other publications, including recent ones that are not cited here (for example DOI:10.1016/j.cell.2016.05.039; 10.1038/s41556-021-00798-4; 10.1038/s41467-021-21984-w) have provided insights into the nature and functions of MDVs. The discussion should thus be updated to include recent publications on MDVs and their role in quality control in relation to the present findings.

Referee #2:

The importance of this paper is the focus on mitochondrial derived vesicles (MDVs). The study of mitochondrial physiology and the release of vesicles containing mitochondrial material, their function and activity, is gaining a lot of attention in recent years. It is shown that functional mitochondria, isolated from Saccharomyces cerevisiae release vesicles. A method for the isolation of these vesicles is described and they are characterized by several techniques. It is shown that the MDVs contain only part of the mitochondrial proteasome and that they harbor a functional ATP synthase complex. Novel is the finding that they seem to fuse with naive mitochondria. An important function of these vesicles would be the delivery of ATP and regeneration of ATP deficient mitochondria.

However, several issues should be considered:
• In the introduction it is written that "While there is thorough research on import of proteins into mitochondria, protein export from the organelle is perceived as nonexistent (Grevel et al., 2019; Neupert and Herrmann, 2007; Schneider, 2020)." However, multiple publications report the presence of mitochondrial material in extracellular vesicles. Moreover, a recent study has shown the release of mitochondria-derived extracellular vesicles (mitovesicles) into the extracellular space that suggest the release of at least some type of MDVs by cells (D'Acunzo et al, 2021). These publications show that not only there is export of mitochondrial proteins from mitochondria but they are also secreted by the cell. Moreover, mass spectrometry show mitochondrial proteins in the MDVs isolated, but not all the mitochondrial proteins that are present in mitochondria. This finding is consistent with the proteins identified in mitovesicles (D'Acunzo et al, 2021). These should be discussed and the papers cited.
• While a novel method to isolate MDVs was developed, it should be written clearly that the isolation is not of MDVs found in the cell but of MDVs generated by free mitochondria in vitro. This is especially meaningful because the mitochondria are not suspended in a buffer that simulates cytosol. While the characterization of the isolated MDVs is very important, it should be clear

that the vesicles may not be identical to those generated in the cytoplasm.
• A crucial part of the method is the isolation of mitochondria and a figure showing that in fact mitochondria are separated from other organelles should be presented. It is stated that the isolated mitochondria are free of contaminations, however, only cytosolic protein is tested. Absence of contamination by other organelles should be shown, by testing for the presence/absence of markers of Golgi, ER, endosomes, etc.
• An explanation should be given in each experiment for the reason specific method and/or material was used. For example, why some experiments are done with pooled material from the supernatant and other experiments are done on OptiPrep fractions. If it is true that the vesicles from the supernatant are all MDVs, why is Optiprep separation done for some experiments?
• "The addition of TCA cycle substrates such as succinate, pyruvate or glycerol had no or insignificant effects on vesicle ATP production (Figure 5A). Moreover, MDVs from yeast devoid of malate dehydrogenase (a TCA cycle enzyme) were able to efficiently drive production of ATP (Figure 5A). Accordingly, we deduce that the membrane potential, per se was sufficient to drive ATP production." How do the authors explain how potential is generated in the absence of NADH?
• The fractions of MDVs proteins following separation by OptiPrep gradient were loaded on acrylamide gels and the gels stained by Comassie blue. A figure showing the fractions (3A) is provided as a validation of gradient efficiency and that fractions 3 to 5 contain most of the MDVs. However, all the gel is showing is that higher amount of proteins is present in these fractions. Western blot analysis should be used to prove that mitochondrial proteins are found mainly in these fractions.
• For all Western blots, molecular weights should be provided.
• For all experiments, the number of experiments conducted should be provided as well as statistical analysis.

Referee #3:

The authors of the manuscript „Mitochondrial Derived Vesicles retain membrane potential and contain a functional ATP synthase" observe the in vitro formation of vesicles from isolated mitochondria. They show that these vesicles have a different protein composition than the initially isolated mitochondria and that these vesicles harbor active F1Fo ATPase of the inner mitochondrial membrane. Based on these observations, they suggest that the observed in vitro vesicles correspond to the so called "mitochondrial derived vesicles" observed in vivo by several groups. The authors propose a very interesting concept, yet my main concern is addressed by the authors themselves in the first paragraph of their discussion section, that the observed vesicles indeed are "ruptured degradation products of mitochondria". To show that this is not the case in vitro is admittedly a very difficult task and the identification of proteins involved in MDV formation would be the only final proof, but of course this is beyond the scope of this publication. Furthermore, it could well be that the loss of "stabilization" of mitochondrial morphology (especially the tubular structure) by the cytoskeleton or contacts to other organelles - that are lacking in vitro - could lead to a slow adaptation in membrane curvature that finally leads to passive vesicle formation over time.

To further support the claim that the observed vesicles are of physiological relevance the authors should focus on the following approaches:

I) I suggest that the authors mimic/force degradation of isolated mitochondria e.g. by sonication, hypotonic buffer, mechanical sheer forces, higher temperatures, mildly oxidative conditions. Next, they should compare the thereby formed vesicles with the vesicles formed under their conditions at 30{degree sign}C in the iso-omolar buffer. A clear difference in size and composition would support their claim.

II) Since vesicle formation is suggested to be a physiological relevant active process, it should follow a defined "kinetics" with saturation after some times since mitochondria in vitro will not be supplied with material to keep forming these vesicles forever. Simple degradation would show a linear or even exponential behavior over time. Therefore, vesicle formation should be followed quantitatively over time.

III) They should further characterize the "mitochondrial remnants" after prolonged incubation at 30{degree sign}C - are they still functional in terms of import, membrane potential etc. Do they show lower levels of the proteins that are claimed to be selectively enriched as cargo in the vesicles?! Intactness and reduced levels of the cargo proteins should be expected if no degradation was the case.

More detailed remarks:

Fig 1A.: The authors should analyze contamination of their mitochondrial fraction with other organelles e.g. ER/vacuolar vesicles.
Fig 1B.: This experiment should also be performed with "mitochondrial remnants" after vesicle formation to show that mitochondria "stay functional".
Fig 3A.: The authors should comment on the dominant 65 kD band which is probably BSA from their assay buffer.
Fig 3C.: The authors should analyze whether there is a correlation of proteins identified and their overall abundance in the mitochondrial proteome. Are the most abundant proteins primarily found? Furthermore, if the vesicles are derived from passive vesicle formation one could expect that especially stable proteins with long turn-over rates are found. Is there any correlation?

Fig 3D.: The sum of the bars is max. 40%. Does this correspond to 40% of mitochondrial proteins or all proteins detected in the proteome analysis?
Fig. 3E.: Cells lacking Mdh1 show severe growth deficit on non-fermentable carbon sources, yet cells for mitochondria isolation for the vesicle formation assay are grown on SG medium (material and methods). Were the dmdh1 cells grown on another medium? Does in vitro vesicle formation depend on the carbon source that was used for cultivation of the cells? Internal mitochondrial structure, especially presence of cristae depends on the carbon source, which in turn could impact on vesicle formation. Does loss of any mitochondrial protein result in lower vesicle formation?! The authors should include the results for a mitochondrial protein that does not impact vesicle formation.
Fig. 4B.: A control protein that is not found in vesicles should be included.
"we were not able to isolate [...] MDVs from datp2" : see comment to Fig. 3E. Probably vesicle formation depends on the initial carbon source cells were cultivated on, and the extend of cristae formation of the inner membrane.
Fig. 5: It is hard to estimate the real ATP production rate of vesicles versus mitochondria. One should normalize the ATP production to vesicle or mitochondrial protein to get a "feeling" for the remaining ATPase activity of the vesicles e.g. is it 10 % or much less?! ATP production itself is difficult to interpret in my eyes. If the vesicles are separated properly from mitochondria, I could imagine that always a contamination with passively/mechanically fragmented mitochondria will be observed. Those contaminations could be responsible for the observed ATP production as well as in the uptake of TMRE.

Response to reviewers

We thank the reviewers for their insightful comments, all of which we addressed by adding a total of 17 new figures and tables to include: 3 new figure-sub-panels, 1 Expanded view figure, 2 Expanded view tables and by significantly modifying the text, as detailed below. We also include 9 figures and 2 tables for the reviewers

Referee#1:

COMMENT: The manuscript by Reut et al. Describes the properties of MDVs isolated from yeast mitochondria and their ability to produce ATP. This is potentially an important finding. However, while the initial characterization of the MDVs is good, the experiments related to the ATP synthase should be more thoroughly done.

RESPONSE: The authors thank the reviewer for his/her positive feedback, indicating that this is potentially an important finding, and for detailed discussion and pertinent suggestions. We believe we have addressed all the comments in the best possible way and hope the reviewer will be satisfied with our revised manuscript. To help the review of this document, we have incorporated panels from the figures as they appear now in the revised version of the manuscript. Please, note that the order of the sub-panels may differ from the figure in the manuscript for presentation purposes.

Major points:

COMMENT:1. If Hxk1 is really a cytosolic protein, then the authors cannot claim that the mitochondrial fraction is free of cytosolic contamination since there are bands corresponding to Hxk1 in the mitochondrial fraction (Fig. 1A). However, as hexokinases are known to associate with mitochondria in some circumstances, this might not be the best control (this is also an issue for the immune-gold experiments). Other controls should be used. Similarly, the authors cannot claim that their mitochondrial preparations are pure based on only one protein evaluated.

RESPONSE: We thank the reviewer for the important comment. We have now added new data for the western blot analysis using new antibodies against proteins known to localize with specific subcellular compartments (Mitochondrial [Tim44, Tim23, Atp2 and Nfs1; Cytosolic

[Hxk1 and Tdh1]; Peroxisomal [Pex13]) (Figure 1A in the manuscript, and shown below). Moreover, based on our LC/MS/MS results analysis which we also updated in the revised manuscript [and added a Principal component analysis (PCA) plot to Expanded View figure 3A] we were able to determine that the summed intensity of the mitochondrial proteins in the mitochondria fraction was more than 99% of the total intensity of all proteins detected in this fraction, reinforcing the purity of our mitochondria. We have now added these results (Figure 1B also attached here below and Expanded View table 4).

Regarding the immune-gold experiments, both negative controls- without primary antibody and with anti-Hxk1, showed minimal or no signal in our vesicles (Figure 4C), suggesting that indeed Hxk1 is not detected in our MDVs. Furthermore, Hxk1 was not detected in mitochondrial or MDVs fractions in our LC/MS/MS which is considered as a very sensitive method (Expanded View tables 1 and 2)

Figure 1

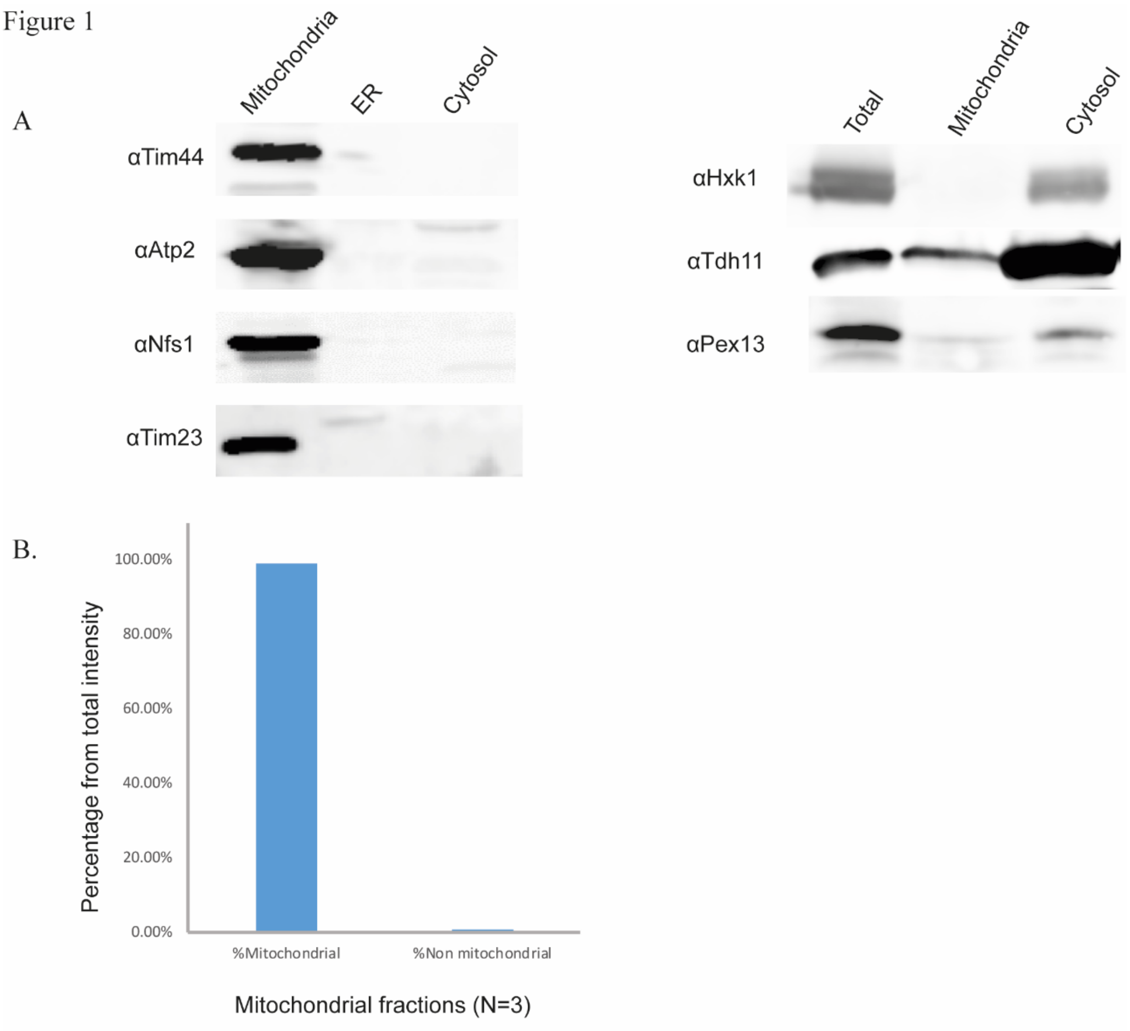

Figure 1A and 1B in the manuscript.

COMMENT:2. While initial reports suggested that MDVs were DRP1-independent, a recent study showed that DRP1 is required for TOM20-positive MDVs (albeit at lower levels than for fission; 10.1038/s41556-021-00798-4). A more thorough comparison of WT and Dnm1 KO cells, including potential morphological differences (e.g. single vs double membrane) and yield is thus required.

RESPONSE: This a valuable comment. We now added a more thorough explanation in the manuscript Discussion. Based on our study, we were able to detect MDVs both for *WT* and *ΔDnm1* yeast cells, as shown in figures 2 and Expanded view figure 1 and as described in an earlier publication from the McBride's Lab (**PMID: 18207745**). Moreover, based on our LC/MS/MS, Tom20 is not detected in our MDVs (Expanded view table 2). All together these results imply that in yeast the formation mechanism of MDVs may be partially different than in higher eukaryotic cells.

COMMENT:3. It is unclear why the Mdh1 and Om45 strains were chosen. The observation that these strains produce less MDVs in vitro is interesting but would need to be confirmed by measuring MDV production in cells. Also, the authors should provide experimental evidence that the reduced MDV production in these strains is the result of a general mitochondrial defect, since this is what they claim.

RESPONSE: We agree with the reviewer that the reason those proteins were chosen was not clear enough. We chose these proteins for two main reasons:

1) Knock out strains of both proteins are viable which enable us to work with the appropriate deletion strains.

2) Each of these proteins represents a different mitochondrial compartment (Om45 is an outer membrane protein while Mdh1 is a matrix protein) and may have a different effect on the mitochondrial function (ΔMdh1 affects the Krebs cycle while ΔOm45 affects import). With respect to mitochondrial function in these knockout backgrounds, a recent study from Shvetsova A, et al. (2021) (PMID: 34713605) has shown that a synthetic lethal phenotype of Om45p and Gem1p disturbs the inner mitochondrial membrane and affects mitochondrial morphology and

function. Moreover, according to our unpublished results, mitochondria from Mdh1 KO cells, produces significantly less ATP as compared to WT, as can be observed in figure 1 for the reviewers [Figures for referees not shown.] .

COMMENT: 4. It would be good to confirm the presence of more than one of the ATP synthase subunits (at least in HEK cells for which there are good antibodies). However, the main question that is not addressed here is whether components of the other ETC complexes are present and could explain the presence of a membrane potential. The presence of these proteins and the effect of ETC inhibition need to be assessed.

RESPONSE: We thank the reviewer for this valuable suggestion. Regarding the first part, we performed Immunogold labeling of MDVs with both anti-Atp1 and anti-Atp2 subunits as shown in figure 4C. Our results confirmed that both Atp1 and Atp2 are present within the vesicles, as compared to the appropriate controls (in the absence of the primary antibody and anti-Hxk1 antibody, a cytosolic protein, non-mitochondrial). Confirming the presence of ATP subunits in HEK293T cells, is indeed one of our follow-up research directions and it is not the main scope of this paper

With respect to the ETC complexes, based on our LC\MS\MS results (Expanded view table 2), we were able to detect most of yeast ETC subunits and this is shown here in table 1 for the Reviewers [Table for referees not shown.] . Additionally, measurements of both cytochrome c oxidase and succinate dehydrogenase activities in MDVs (32 nmol/min/mg and 22 nmol/min/mg protein respectively) indicated that ETC activity is present in MDVs. Although the ETC subunits are detected in our MDVs, as shown in our results (figure 5A), the only crucial substrate needed for this reaction is ADP. Thus, we hypothesized that a proton gradient present across the MDVs membrane might be sufficient to drive the ATP synthesis. To this end, we determined ATP production levels at various pHs as shown herein in figure 2 for the Reviewers [Figures for referees not shown.]. It is clear that the pH in the surrounding medium affects the ATP generation capacity of MDVs. When lowering the external buffer pH to 7 (the vesicles lumen is presumably pH 7.8 as the matrix pH) increasing H^+ in the surrounding environment which in turn increased ATP production. *Vice versa*, when the external pH was raised (pH=7.8), less ATP was generated. These results suggest that the membrane potential, *per se*, was sufficient to drive ATP production. Still as other ETC complexes are present, we cannot exclude that these might also contribute to the membrane potential.

COMMENT: 5 .The small effect of cccp and oligomycin is worrisome and could suggest a mechanism other than the ATP synthase.

RESPONSE: We agree with the reviewer that the effect of CCCP and oligomycin is partial but we see this in a positive manner. In order to isolate our MDVs, we isolate mitochondria, freeze them and then proceed with MDVs purification. The purified MDVs are frozen and these organelles are used for further applications such as ATP production (Figure 5). These two freezing steps might cause uncoupling (between the electron transport and ATP synthesis), thus, reducing the MDVs ATP levels. However, despite such limitations of the assay, we could still detect a significant activity, strengthening the power of this method. Moreover, both CCCP and Oligomycin are poorly dissolvable in aqueous solutions (http://www.chemspider.com/Chemical-Structure.2504.html, http://www.chemspider.com/Chemical-Structure.10308285.html) and might not penetrate the MDVs thus affecting the actual concentration that MDVs were exposed to.

COMMENT: 6. The ability of the MDVs to produce ATP is intriguing. How much ATP do these MDVs produce relative to mitochondria?

RESPONSE: We have now estimated the ATP production relative to protein concentration, both in mitochondria (246496±7824 RLU/min/µg protein) and vesicles (140260±4623 RLU/min/µg protein) Accordingly, MDVs retain a 57% ATP production capacity compared to mitochondria when normalized to protein content. This information is now included in the results section under the paragraph "Functional importance of MDVs".

COMMENT: Do they consume oxygen?

RESPONSE: Due to technical constraints we could not directly measure MDVs oxygen consumption. However, we did measure cytochrome c oxidase complex IV, the final electron acceptor of the mitochondrial electron transfer chain (ETC) which utilizes oxygen as a substrate) in MDVs (32 nmol/min/mg protein) and compared it to mitochondria (361 nmol/min/mg protein). Accordingly, MDVs do utilize oxygen but to a lesser extent than mitochondria. Notably as a control we also measured succinate dehydrogenase activity (22 and 226 nmol/min/mg protein in MDVs and mitochondria, respectively) indicating that ETC is present in MDVs but is approximately ten times less active.

COMMENT: The rescue experiment is also very interesting but lacks an MDV alone control. Also, the possibility that MDVs stay associated with mitochondria in the rescue experiment should be addressed. Is the ATP synthase actually transferred to recipient mitochondria?

RESPONSE: We thank the reviewer for this important comment, and thus need to further clarify our assay. For the complementation experiments the mitochondria were washed prior to the ATP production measurements (please see the methods section-" ATP production and complementation
"). We then validated the efficiency of our washing by measuring ATP content in the supernatant after each wash and after the second wash we detect neglectable ATP production. The uptake experiments (Figure 5), demonstrate that no vesicles remnants are detected after the second wash.
The image stream analysis results showed co-localization of mCherry labelled mitochondria and GFP labelled MDVs (Figure 3 for the Reviewers)[Figures for referees not shown.], suggesting that the mitochondria and the vesicles are co-localized and interact.

COMMENT:6. MDVs with a membrane potential have been observed before (10.1016/j.cub.2011.11.057).
COMMENT 7. The manuscript seems to rely heavily on the Neuspiel paper. However, a number of other publications, including recent ones that are not cited here (for example DOI:10.1016/j.cell.2016.05/10.1038 ;039.s41556-021-00798-4; 10.1038/s41467-021-21984-w) have provided insights into the nature and functions of MDVs. The discussion should thus be updated to include recent publications on MDVs and their role in quality control in relation to the present findings.
RESPONSE: We thank the reviewer for this important suggestion. Corrected. We have now addressed these updated publications in our manuscript (see the Discussion chapter. Pages 9-11).

Referee #2:

COMMENT: The importance of this paper is the focus on mitochondrial derived vesicles (MDVs). The study of mitochondrial physiology and the release of vesicles containing mitochondrial material, their function and activity, is gaining a lot of attention in recent years. It is shown that functional mitochondria, isolated from Saccharomyces cerevisiae release vesicles. A method for the isolation of these vesicles is described and they are characterized by several techniques. It is shown that the MDVs contain only part of the mitochondrial proteasome and that they harbor a functional ATP synthase complex. Novel is the finding that they seem to fuse with naive mitochondria. An important function of these vesicles would be the delivery of ATP and regeneration of ATP deficient mitochondria.

RESPONSE: The authors thank the reviewer for the careful reading of the manuscript, detailed discussion and pertinent suggestions. We have now addressed the comments raised by the reviewer, one by one, which we trust has significantly increased the quality of our manuscript.
To help the review of this document, we have incorporated panels from the figures as they appear in the revised version of the manuscript. Please, note that the order of the sub-panels may differ from the figure in the manuscript for presentation purposes.

However, several issues should be considered:
COMMENT: 1. In the introduction it is written that "While there is thorough research on import of proteins into mitochondria, protein export from the organelle is perceived as nonexistent (Grevel et al., 2019; Neupert and Herrmann, 2007; Schneider, 2020)." However, multiple publications report the presence of mitochondrial material in extracellular vesicles. Moreover, a recent study has shown the release of mitochondria-derived extracellular vesicles (mitovesicles) into the extracellular space that suggest the release of at least some type of MDVs by cells (D'Acunzo et al, 2021). These publications show that not only there is export of mitochondrial proteins from mitochondria but they are also secreted by the cell. Moreover, mass spectrometry shows mitochondrial proteins in the MDVs isolated, but not all the mitochondrial proteins that are present in mitochondria. This finding is consistent with the proteins identified in mitovesicles (D'Acunzo et al, 2021).

RESPONSE: We thank the referee for these important comments. Corrected. These issues are now addressed in the manuscript Discussion chapter and the papers are cited.

COMMENT:2. While a novel method to isolate MDVs was developed, it should be written clearly that the isolation is not of MDVs found in the cell but of MDVs generated by free mitochondria in vitro. This is especially meaningful because the mitochondria are not suspended in a buffer that simulates cytosol. While the characterization of the isolated MDVs is very important, it should be clear that the vesicles may not be identical to those generated in the cytoplasm.

RESPONSE: This is a valuable comment. Accordingly, it is now stated very clearly both in the discussion and in the materials and methods sections that the vesicles are isolated from purified mitochondria. Regarding cytosol- while the characterization of the isolated MDVs is very important, we would like to emphasize that in our work, we described a different method for isolation of MDVs directly from isolated mitochondria and hence, they may not be identical to those generated in the cytoplasm.

COMMENT:3. A crucial part of the method is the isolation of mitochondria and a figure showing that in fact mitochondria are separated from other organelles should be presented. It is stated that the isolated mitochondria are free of contaminations, however, only cytosolic protein is tested. Absence of contamination by other organelles should be shown, by testing for the presence/absence of markers of Golgi, ER, endosomes, etc.

RESPONSE: We thank the reviewer for the important comment;
We have now added new data for the western blot analysis using new antibodies against different subcellular compartment (Mitochondrial [Tim44, Tim23, Atp2 and Nfs1; Cytosolic [Hxk1 and Tdh1]; Peroxisomal [Pex13]) (Figure 1A).
Moreover, based on our LC/MS/MS analysis we can deduce that the summed intensity of the mitochondrial proteins in the mitochondrial fraction was more than 99% of the total intensity of all proteins detected in this fraction reinforcing the purity of our mitochondria. We now added these results (Figure 1B and new Expanded view table 4)

COMMENT: An explanation should be given in each experiment for the reason specific method and/or material was used. For example, why some experiments are done with pooled material from the supernatant and other experiments are done on OptiPrep fractions. If it is true that the vesicles from the supernatant are all MDVs, why is Optiprep separation done for some experiments?

RESPONSE: We understand the reviewer's point and thus need to further clarify our assay. OptiPrep gradient is a well-known and defined method for EV isolation for proteomics analysis (**PMID: 33608523; PMID: 31825165**). This step is performed in order to take extra caution with the purity of the vesicles. The protocol that we used is the following: The initial vesicle pellet was resuspended in SH buffer and further purified on a continuous 10–30% OptiPrep gradient made up in NTE buffer (137mM NaCl, 1mM EDTA and 10mM Tris, pH 7.4). Then centrifuged at 150,000 g for18 h at 4 °C, in a SW41 rotor Fractions were collected from the top and Methanol was added prior freezing the MDVs in -80°C for 18 hours. Each fraction was centrifuged 30 minutes at 4°C, at 10,000g and pellets were suspended in sample buffer and

loaded on acrylamide gel. We now updated this more clarified assay to the materials and methods section.

COMMENT: The addition of TCA cycle substrates such as succinate, pyruvate or glycerol had no or insignificant effects on vesicle ATP production (Figure 5A). Moreover, MDVs from yeast devoid of malate dehydrogenase (a TCA cycle enzyme) were able to efficiently drive production of ATP (Figure 5A). Accordingly, we deduce that the membrane potential, per se was sufficient to drive ATP production." How do the authors explain how potential is generated in the absence of NADH?

RESPONSE: We thank the reviewer for this insightful comment. To address this, we have determined ATP production levels at various pHs. Accordingly, we hypothesize that a proton gradient present across the MDVs membrane might be sufficient to drive the ATP synthesis. As shown in figure 2 for the Reviewers, it is clear that the pH in the surrounding medium affects the ATP generation capacity of MDVs. When lowering the external buffer pH to 7 (the vesicles lumen is presumably pH 7.8 as the matrix pH) increasing H+ in the surrounding environment, ATP production increased (Figure 2 for the Reviewers). Vice versa, when the external pH was raised (pH=7.8), less ATP was generated (Figure 2 for the Reviewers). These data suggest that the membrane potential, per se is sufficient to drive ATP production. Still as other ETC complexes are present, we cannot exclude that these might also contribute to the membrane potential.

COMMENT: The fractions of MDVs proteins following separation by OptiPrep gradient were loaded on acrylamide gels and the gels stained by Coomassie blue. A figure showing the fractions (3A) is provided as a validation of gradient efficiency and that fractions 3 to 5 contain most of the MDVs. However, all the gel is showing is that higher amount of proteins is present in these fractions. Western blot analysis should be used to prove that mitochondrial proteins are found mainly in these fractions.

RESPONSE: We thank the reviewer for this important comment. Accordingly, we ran the OptiPrep gradient fractions on acrylamide gel and performed western blotting analysis

usingAnti-Mdh1 antibody. As shown in figure 4 for the Reviewers, Mdh1, which is a soluble mitochondrial protein, is mainly found in fractions 2 to 6 which are the most concentrated fractions for protein content (Coomassie gel). Furthermore, as shown in figure 4C for the Reviewers[Figures for referees not shown.], Hxk1, a cytosolic protein, is not detected at all in the vesicle fractions.

Referee#3:

COMMENT:

The authors of the manuscript „Mitochondrial Derived Vesicles retain membrane potential and contain a functional ATP synthase" observe the in vitro formation of vesicles from isolated mitochondria. They show that these vesicles have a different protein composition than the initially isolated mitochondria and that these vesicles harbor active F1Fo ATPase of the inner mitochondrial membrane. Based on these observations, they suggest that the observed in vitro vesicles correspond to the so called "mitochondrial derived vesicles" observed in vivo by several groups. The authors propose a very interesting concept, yet my main concern is addressed by the authors themselves in the first paragraph of their discussion section, that the observed vesicles indeed are "ruptured degradation products of mitochondria". To show that this is not the case in vitro is admittedly a very difficult task and the identification of proteins involved in MDV formation would be the only final proof, but of course this is beyond the scope of this publication. Furthermore, it could well be that the loss of "stabilization" of mitochondrial morphology (especially the tubular structure) by the cytoskeleton or contacts to other organelles - that are lacking in vitro - could lead to a slow adaptation in membrane curvature that finally leads to passive vesicle formation over time.
To further support the claim that the observed vesicles are of physiological relevance the authors should focus on the following approaches:

COMMENT: I) I suggest that the authors mimic/force degradation of isolated mitochondria e.g. by sonication, hypotonic buffer, mechanical sheer forces, higher temperatures, mildly oxidative conditions. Next, they should compare the thereby formed vesicles with the vesicles formed under their conditions at 30{degree sign}C in the iso-omolar buffer. A clear difference in size and composition would support their claim.

RESPONSE: We thank the reviewer for the insightful comments. We isolated vesicles from mitochondrial incubated 24 hours, washed and then incubated for additional 24 hours at 30oC (after 48h the mitochondria are dysfunctional and have no import capabilities). As can be shown in figure 5 for the Reviewers[Figures for referees not shown.], no vesicles were detected by NTA analysis after the second incubation (for comparison below figure 5 for the Reviewer we also added figure 2A from the manuscript showing typical MDVs isolated from 24h mitochondria). Together these results suggest that non-functional mitochondria (48h at 30o C) are incapable of MDVs production.

COMMENT:II) Since vesicle formation is suggested to be a physiological relevant active process, it should follow a defined "kinetics" with saturation after some times since mitochondria in vitro will not be supplied with material to keep forming these vesicles forever. Simple

degradation would show a linear or even exponential behavior over time. Therefore, vesicle formation should be followed quantitatively over time.

RESPONSE: We thank the reviewer for this important comment. Following this comment, we performed vesicles isolation at three time points: 4 hours, 24 hours and 48 hours. After 4 hours according to NTA measurement vesicle concentration of 1.8*10^11 particles/ml were obtained (figure 6 for the Reviewers)[Figures for referees not shown.]. After 24 hours we observed 2.6*10^11 particles/ml, while no additional vesicles formation was observed after 48 hours. Thus, as the reviewer suggests there is a saturation in accord with declining vesicle formation capacity over time.

COMMENT: III) They should further characterize the "mitochondrial remnants" after prolonged incubation at 30°C - are they still functional in terms of import, membrane potential etc. Do they show lower levels of the proteins that are claimed to be selectively enriched as cargo in the vesicles?! Intactness and reduced levels of the cargo proteins should be expected if no degradation was the case.

RESPONSE: We thank the reviewer for this comment. From technical reasons we were not able to characterize the “mitochondrial remnants” in terms of cargo proteins. Instead, we measured ATP generation capacity of remnant mitochondrial (Figure 7 for the Reviewers) [Figures for referees not shown.]. We found that

after 4 hours of incubation, the mitochondria retained 33 percentage capacity as compared to mitochondria without incubation, while after 24 hours the capacity was significantly decreased (14% of fresh mitochondria). Thus, the remnant mitochondria are indeed less active in terms of ATP production.

COMMENT: Fig 1A.: The authors should analyze contamination of their mitochondrial fraction with other organelles e.g. ER/vacuolar vesicles.

RESPONSE: We agree with the reviewer and have now added new antibodies against proteins found in different subcellular compartments ((Mitochondrial [Tim44, Tim23, Atp2 and Nfs1; Cytosolic [Hxk1 and Tdh1]; Peroxisomal [Pex13]) (Figure 1A). Moreover, based on our LC/MS/MS analysis we can deduce that the summed intensity of the mitochondrial proteins in the mitochondria fraction was more than 99% of the total intensity of all proteins detected in this fraction (Figure 1B and Expanded view table 4) reinforcing the purity of our mitochondria, and we now added these results (Figure 1B and Expanded view table 4).

COMMENT: Fig 1B.: This experiment should also be performed with "mitochondrial remnants" after vesicle formation to show that mitochondria "stay functional."

RESPONSE: The reviewer is raising a valuable comment. Following this comment, we performed 1) an *in-vitro* import assay as well as 2) ATP generation measurements in freshly isolated mitochondria, and in mitochondrial remnants upon vesicles formation.

1) For the *in-vitro* import assay the labeled transcribed Su9-DHFR precursor, was incubated with pure mitochondria, and the efficiency of the import process, was monitored by the formation of the mature form of the protein (PMID 17445722). As shown in figure 8 for the Reviewers, the mature form of the protein was detected after 1 minute, while after 12 minutes, most of the protein population was processed. These data indicate that the mitochondria are intact and retain a membrane potential. On the contrary, when incubating the labeled in vitro-transcribed Su9-DHFR precursor with 24 or 48 hours' mitochondria (incubated for 24 or 48 hours in order to generate MDVs), the mature form was not detected (Figure 8 for the Reviewers).

2) ATP capacity of 24 hours incubated mitochondria were 14% of the ATP capacity as compared to fresh mitochondria (As can be shown in figure 7 for the Reviewers)[Figures for referees not shown.]. Overall, these results suggest that in order to generate vesicles, the mitochondria should be active, but after mitochondrial incubation, mitochondrial activity decreased significantly and we hypothesize that one possible explanation is that these mitochondria "donate" their activity to MDVs.

COMMENT: Fig 3A.: The authors should comment on the dominant 65 kD band which is probably BSA from their assay buffer.

RESPONSE: According to the literature (PMID: 34322205; 31069026; 23683579; 30973950), OptiPrep gradient (60% iodixanol), is a well-established method accepted to isolate vesicles. Accordingly, the fractions that are loaded on the Coomassie gels contain residual amounts of BSA, therefore its less reasonable that the 65kDa band represent it.

COMMENT: Fig 3C.: The authors should analyze whether there is a correlation of proteins identified and their overall abundance in the mitochondrial proteome. Are the most abundant proteins primarily found? Furthermore, if the vesicles are derived from passive vesicle formation one could expect that especially stable proteins with long turn-over rates are found. Is there any correlation.

RESPONSE: We thank the reviewer for this insightful comment. As can be found in Expanded view tables 1-3 in the manuscript, MDVs proteome consist of abundant and non-abundant proteins. Furthermore, as shown in table 2 for the Reviewers[Table for referees not shown.], when looking on the most dominant mitochondrial proteins identified in the mitochondrial fractions, no correlation was found between the protein half life time and the ratio of the protein intensity in the EV compared to the original mitochondrial fraction. (The half-life time was taken from PMID: 28918244).

Regarding the question of active or passive MDVs formation, in our hands, mitochondria derived from ΔATP2 cells, significantly reduced the level of vesicle production. Accordingly, as shown in figure 5C in the manuscript, these mitochondria are indeed active. Moreover, as shown in figure 6 and 7 for the Reviewers, after 24 hours of incubation, the mitochondria are less active and hence produce much less MDVs than after 4 hours of incubation. Together, these results support the demand for active mitochondria in order to produce MDVs.

COMMENT: Fig 3D.: The sum of the bars is max. 40%. Does this correspond to 40% of mitochondrial proteins or all proteins detected in the proteome analysis?

RESPONSE: We agree with the reviewer. We now display figure 3D in a clearer form. As shown in the new figure 3D (also added below), we now present the percentage of the different groups of mitochondrial proteins, from the total intensity of mitochondrial proteins of each fraction (mitochondria or MDVs).

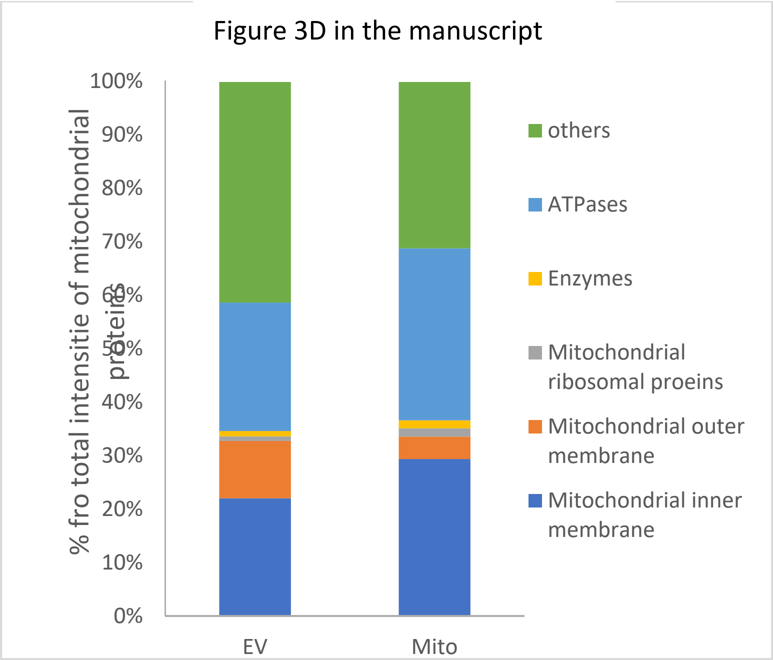

COMMENT: Fig. 3E.: Cells lacking Mdh1 show severe growth deficit on non-fermentable carbon sources, yet cells for mitochondria isolation for the vesicle formation assay are grown on SG medium (material and methods). Were the dmdh1 cells grown on another medium? Does in vitro vesicle formation depend on the carbon source that was used for cultivation of the cells? Internal mitochondrial structure, especially presence of cristae depends on the carbon source, which in turn could impact on vesicle formation. Does loss of any mitochondrial protein result in lower vesicle formation?! The authors should include the results for a mitochondrial protein that does not impact vesicle formation.

RESPONSE: We understand the reviewer comment. Notably only the starter cultures were grown in SG. Subsequently for all experiments, all yeast strains were resuspended and cultured in a more restrictive medium containing 2% lactate and 0.05% glucose as carbon source, in order to isolate mitochondria (lactate is a non-fermentable carbon source). Cells lacking Mdh1 were still able to grow in this medium but as expected, showed decreased growth. Nevertheless, active mitochondria derived from Mdh1 cells grown in this medium were able to produce MDVs (Figure 3E). Moreover, in order to normalize vesicle isolation from different yeast strains, the total amount of mitochondria utilized from each strain for MDVs formation, was identical.

Thus, differences in vesicle formation were due to the strain and not the carbon source. Moreover, as shown in figure 3E, we have showed that mutations in genes encoding for mitochondrial proteins present in MDVs, such as Mdh1 and Om45, decreases the concentration of MDVs as compared to *wild type*.

In contrast, when we isolated MDVs from ΔDnm1 mitochondria, (the mitochondrial fission GTPase) no decrease in MDVs concentration was detected (as shown in figure 9 for the reviewers)[Figures for referees not shown.]. Thus, the loss of a mitochondrial proteins, such as Dnm1, has no impact on the vesicle formation. These results suggest that MDV formation is rather an active process.

Fig. 4B.: A control protein that is not found in vesicles should be included.

RESPONSE: Corrected. Please see figure 4B for the Reviewers.

"we were not able to isolate [...] MDVs from datp2": see comment to Fig. 3E. Probably vesicle formation depends on the initial carbon source cells were cultivated on, and the extend of cristae formation of the inner membrane.

RESPONSE: We thank the reviewer for this comment, and we further want to clarify our assay. In our approach we cultured all yeast cells in 2% lactate and 0.05% glucose containing medium. In this medium most cell types showed no limitations to grow and to use these carbon sources. We than isolated mitochondria and used the same total amount of mitochondria (mg/ml) to isolate secreted vesicle. In this manner, we normalized the vesicle production levels according to mitochondria concentration and avoided differences in cells growth. Furthermore, when we isolated vesicles, we showed variability in MDVs formation dependent on the genetic background and the mitochondrial energy condition (Figure 3E), although all these mitochondria were isolated under the same conditions.

COMMENT: Fig. 5: It is hard to estimate the real ATP production rate of vesicles versus mitochondria. One should normalize the ATP production to vesicle or mitochondrial protein to get a "feeling" for the remaining ATPase activity of the vesicles e.g. is it 10 % or much less?!

ATP production itself is difficult to interpret in my eyes. If the vesicles are separated properly from mitochondria, I could imagine that always a contamination with passively/mechanically fragmented mitochondria will be observed. Those contaminations could be responsible for the observed ATP production as well as in the uptake of TMRE.

RESPONSE: We have now estimated the ATP production relative to protein concentration, both in mitochondria (246496±7824 RLU/min/µg protein) and vesicles (140260±4623 RLU/min/µg protein). Accordingly, MDVs retain a 57% ATP production capacity compared to mitochondria when normalized to protein content. This information is now included in the results section under the paragraph “Functional importance of MDVs”.

To the best of our knowledge mechanically fragmented mitochondria are not able to retain a membrane potential and are thus not capable of ATP production (in fact, free ATP synthase residing in mitochondrial membrane fragments could even act in the reverse reaction by hydrolyzing ATP (PMID: 23356252).

Dear Dr. Regev-Rudzki,

Thank you for the submission of your revised manuscript to our editorial offices. I have now received the reports from the three referees that I asked to re-evaluate your study, you will find below. As you will see, the referees now fully support the publication of your study in EMBO reports. Referee #1 has some comments and suggestions to improve the manuscript, I ask you to address in a final revise manuscript. Please also provide a final p-b-p-response addressing these remaining points.

Moreover, I have these editorial requests I also ask you to address:

- Please provide the abstract written in present tense throughout.

- We plan to publish your manuscript in the Report format, as there are not more than 5 main figures. For a Scientific Report we require that results and discussion sections are combined in a single chapter called "Results & Discussion". Please do this for your manuscript. For more details, please refer to our guide to authors:
http://www.embopress.org/page/journal/14693178/authorguide#researcharticleguide

- Please add up to five keywords to the manuscript text, below the abstract.

- Please add a data availability section to the manuscript text and move the information on deposited datasets (mass spectrometry proteomics) there. Please remove the referee token from the data availability section, add a direct link to the dataset and make sure that the dataset is public latest upon online publication of the study.

- We updated our journal's competing interests policy in January 2022 and request authors to consider both actual and perceived competing interests. Please review the policy https://www.embopress.org/competing-interests and update your competing interests if necessary. Please name this section 'Disclosure and Competing Interests Statement' and put it after the Acknowledgements section.

- Please order the manuscript sections like this (using these names as headings):
Title page - Abstract - Key Words - Introduction - Results & Discussion - Materials and Methods - Data availability section - Acknowledgements - Disclosure and Competing Interests Statement - References - Figure legends - Expanded View Figure Legends

- For the Expanded View figures, please follow the nomenclature Figure EVx (name and call-outs) and add the title 'Expanded View Figure Legends' (see above).

- Regarding data quantification and statistics, please make sure that the number "n" for how many independent experiments were performed, their nature (biological versus technical replicates), the bars and error bars (e.g. SEM, SD) and the test used to calculate p-values is indicated in the respective figure legends (also for potential EV figures and all those in the final Appendix). Please also check that all the p-values are explained in the legend, and that these fit to those shown in the figure. Please provide statistical testing where applicable. Please avoid the phrase 'independent experiment', but clearly state if these were biological or technical replicates. Please also indicate (e.g. with n.s.) if testing was performed, but the differences are not significant. In case n=2, please show the data as separate datapoints without error bars and statistics. See also:
http://www.embopress.org/page/journal/14693178/authorguide#statisticalanalysis

If n<5, please show single datapoints for diagrams. Presently, it seems there are diagrams with partial statistics.

- Please add scale bars of similar style and thickness to the microscopic images, using clearly visible black or white bars (depending on the background). Please place these in the lower right corner of the images. Please do not write on or near the bars in the image but define the size in the respective figure legend.

- Please provide a fully completed author checklist, providing information in column D (select responses using the pull-down menu).

- Appendix Tables 1 and 2 are datasets. Please upload these as original excel files datasets using the names 'Dataset EV1' and 'Dataset EV2'. Please add a title and a legend to the first TAB of the excel file and change the callouts to these items using 'Dataset EV1' and 'Dataset EV2'. Finally, please remove the Appendix file.

- Please provide a fully completed author checklist, providing information in column D (select responses).

- Please use our reference format (names should not be in uppercase, the year in brackets and we need et al if there are more then10 authors):
http://www.embopress.org/page/journal/14693178/authorguide#referencesformat

- Please make sure that all the funding information is also entered into the online submission system and that it is complete and similar to the one in the acknowledgement section of the manuscript text file.

- Please make sure that all figure panels are called out separately and sequentially (main, and EV figures). Presently, there seem to be no callouts for Fig. EV4 and no separate callouts for most of the panels of the other EV figures. Please check.

- The labeling of the axes and the legends in many diagrams is rather small. Could this be improved?

- There are 5 tables uploaded in one excel file. Tables 1-3 and 5 are satasets. Please name these files Dataset EV1-EV4, add title and legend on the first TAB of the excel file and upload these as separate dataset files. Table 4 (the short table) should be named Table EV1 and uploaded as single excel file (with title and legend on the first TAB) as EV table. Finally, please make sure that all these items are called out correctly using the new names. Finally, please remove the legends for these items from the main manuscript text file.

- As I already indicated in my first decision letter, we now request the publication of original source data with the aim of making primary data more accessible and transparent to the reader. It seems you have been contacted already by our source data coordinator, who indicated which figure panels we would need source data for. I attach again the source data checklist and the FAQ. Please make sure that all the requested source data is provided. Please upload all source data for one figure as one pdf per figure or (if there is more than one file) ZIPed together as one folder. Finally, please upload the filled in source data checklist with your final revised files.

- Finally, please find attached a word file of the manuscript text (provided by our publisher) with changes we ask you to include in your final manuscript text and comments. Please use the attached file as basis for further revisions and provide your final manuscript file with track changes, in order that we can see any modifications done.

In addition, I would need from you:
- a short, two-sentence summary of the manuscript (not more than 35 words).
- two to four short bullet points highlighting the key findings of your study (two lines each).
- a schematic summary figure that provides a sketch of the major findings (not a data image) in jpeg or tiff format (with the exact width of 550 pixels and a height of not more than 400 pixels) that can be used as a visual synopsis on our website.

I look forward to seeing the final revised version of your manuscript when it is ready. Please let me know if you have questions regarding the revision.

Please use this link to submit your revision: https://embor.msubmit.net/cgi-bin/main.plex

Best,

Achim Breiling
Senior Editor
EMBO Reports

Referee #1:

In their response to the reviewers' comments, the authors satisfactorily address all my concerns. However, I am not sure why most of the new data addressing the reviewers' comments is presented as figures for reviewers only rather than including this important information in the manuscript. The following should be included within the manuscript:
1) The number of MDVs generated in Dnm1 mutants (the figure la labeled Fig. 4B in the response to reviewers but not included in Fig. 4)
2) At least some of the information provided to clarify the status of ETC components other than the ATP synthase in MDVs (in relation to Fig. 5)(Table 1 for reviewers, Figure 2 for reviewers, COX/SDH activity)
3) Some of the data provided to Reviewer 3 concerning the specificity of the assay (Fig. for reviewers 3, 5-7).

Minor points:
4) Although important, Expanded View figure 1A (PCA analysis) is not mentioned anywhere in the manuscript. The labels on the figure are also small and hard to read.
5) Figure 1B should show error bars if n=3.

Referee #2:

The authors extensively responded to the critiques.

Referee #3:

The authors sufficiently addressed all point raised in my initial review. The improved manuscript provides interesting experimental evidence for the formation of mitochondrial derived vesicles (MDVs) in vitro.

To:
Dr Achim Breiling
Senior Editor *EMBO Reports*

Dear Dr. Breiling,

Following the editorial requests, we have now modified the manuscript accordingly. Below are point by point response.

-Please provide the abstract written in present tense throughout.

We have changed the abstract as requested

-Please add up to five keywords to the manuscript text, below the abstract.
We have added five keywords as requested: ATP Synthase/ Membrane potential/ Mitochondria/ Mitochondrial Derived Vesicles/ Protein distribution.

-Please add a data availability section to the manuscript text and move the information on deposited datasets (mass spectrometry proteomics) there. Please remove the referee token from the data availability section, add a direct link to the dataset and make sure that the dataset is public latest upon online publication of the study.

We have added a data availability section to the manuscript and move the information on deposited datasets (mass spectrometry proteomics) there, as requested

-We updated our journal's competing interests policy in January 2022 and request authors to consider both actual and perceived competing interests. Please review the policy https://www.embopress.org/competing-interests and update your competing interests if necessary. Please name this section 'Disclosure and Competing Interests Statement' and put it after the Acknowledgements section.

We have added the 'Disclosure and Competing Interests Statement' section as requested.

-Please order the manuscript sections like this (using these names as headings):
Title page - Abstract - Key Words - Introduction - Results & Discussion - Materials and Methods - Data availability section - Acknowledgements - Disclosure and Competing Interests Statement - References - Figure legends - Expanded View Figure Legends.

We have ordered the figures and the manuscript sections as requested.
-For the Expanded View figures, please follow the nomenclature Figure EVx (name and call-outs) and add the title 'Expanded View Figure Legends' (see above).

We have changed the nomenclature as requested.

-Regarding data quantification and statistics, please make sure that the number "n" for how many independent experiments were performed, their nature (biological versus technical replicates), the bars and error bars (e.g. SEM, SD) and the test used to calculate p-values is indicated in the respective figure legends (also for potential EV figures and all those in the final Appendix). Please also check that all the p-values are explained in the legend, and that these fit to those shown in the figure. Please provide statistical testing where applicable. Please avoid the phrase 'independent experiment', but clearly state if these were biological or technical replicates. Please also indicate (e.g. with n.s.) if testing was performed, but the differences are not significant. In case n=2, please show the data as separate datapoints without error bars and statistics.
We have addressed all these statistic requirements as requested

If n ,5>please show single datapoints for diagrams. Presently, it seems there are diagrams with partial statistics.

-Please add scale bars of similar style and thickness to the microscopic images, using clearly visible black or white bars (depending on the background). Please place these in the lower right corner of the images. Please do not write on or near the bars in the image but define the size in the respective figure legend.
We made the changes as requested and added new figure 2C since the former figure had the scale bar as part of the original figure.

-Please provide a fully completed author checklist, providing information in column D (select responses using the pull-down menu).
Done

-Appendix Tables 1 and 2 are datasets. Please upload these as original excel files datasets using the names 'Dataset EV1' and 'Dataset EV2'. Please add a title and a legend to the first TAB of the excel file and change the callouts to these items using 'Dataset EV1' and 'Dataset EV2'. Finally, please remove the Appendix file.
We have made the changes as requested.

-Please provide a fully completed author checklist, providing information in column D (select responses).
Completed

-Please use our reference format (names should not be in uppercase, the year in brackets and we need et al if there are more then10 authors):
http://www.embopress.org/page/journal/14693178/authorguide#referencesformat
We have changed the format as requested.

-Please make sure that all the funding information is also entered into the

online submission system and that it is complete and similar to the one in the acknowledgement section of the manuscript text file.

-Please make sure that all figure panels are called out separately and sequentially (main, and EV figures). Presently, there seem to be no callouts for Fig. EV4 and no separate callouts for most of the panels of the other EV figures. Please check.

We have made the changes as requested.

-The labeling of the axes and the legends in many diagrams is rather small. Could this be improved?

We improved the axes and legends as requested.

-There are 5 tables uploaded in one excel file. Tables 1-3 and 5 are datasets. Please name these files Dataset EV1-EV4, add title and legend on the first TAB of the excel file and upload these as separate dataset files. Table 4 (the short table) should be named Table EV1 and uploaded as single excel file (with title and legend on the first TAB) as EV table. Finally, please make sure that all these items are called out correctly using the new names. Finally, please remove the legends for these items from the main manuscript text file.

We have made the changes as requested.

-As I already indicated in my first decision letter, we now request the publication of original source data with the aim of making primary data more accessible and transparent to the reader. It seems you have been contacted already by our source data coordinator, who indicated which figure panels we would need source data for. I attach again the source data checklist and the FAQ. Please make sure that all the requested source data is provided. Please upload all source data for one figure as one pdf per figure or (if there is more than one file) ZIPed together as one folder. Finally, please upload the filled in source data checklist with your final revised files.

We provided all sources of data requested.

-Finally, please find attached a word file of the manuscript text (provided by our publisher) with changes we ask you to include in your final manuscript text and comments. Please use the attached file as basis for further revisions and provide your final manuscript file with track changes, in order that we can see any modifications done.

We have made the changes requested for this file. All changes are marked in track changes.

In addition, I would need from you:
-a short, two-sentence summary of the manuscript (not more than 35 words).

In this work, we have isolated Mitochondrial Derived Vesicles (MDVs) for the first time and reveal a possible delivery mechanism of ATP producing vesicles. This encapsulated ATP synthase complex can potentially regenerate ATP deficient mitochondria and may participate in organelle to organelle communication.

-two to four short bullet points highlighting the key findings of your study (two lines each).

- Selective mitochondrial proteins are loaded into MDVs
- MDVs have a membrane potential and carry a functional F1Fo ATP synthase complex with the capability to produce ATP
- MDVs acquire a major activity and function of mitochondria and, may possibly transfer this ability from one organelle to the other
- MDVs enable export of proteins from the mitochondria into the cellular environment (such a pathway has not been identified by any other mechanism).

-a schematic summary figure that provides a sketch of the major findings (not a data image) in jpeg or tiff format (with the exact width of 550 pixels and a height of not more than 400 pixels) that can be used as a visual synopsis on our website. The figure was generated by the BioRender software (**https://app.biorender.com/**).

Please use this link to submit your revision: https://embor.msubmit.net/cgi-bin/main.plex

----
Referee #1:

In their response to the reviewers' comments, the authors satisfactorily address all my concerns. However, I am not sure why most of the new data addressing the reviewers' comments is presented as figures for reviewers only rather than including this important information in the manuscript. The following should be included within the manuscript:
(1The number of MDVs generated in Dnm1 mutants (the figure la labeled Fig. 4B in the response to reviewers but not included in Fig. 4)
As suggested by the reviewer we have now added this figure as an EV figure 2B.

(2At least some of the information provided to clarify the status of ETC components other than the ATP synthase in MDVs (in relation to Fig. 5)(Table 1 for reviewers, Figure 2 for reviewers, COX/SDH activity)

The information regarding ETC components are available at Expanded View Dataset no' 2 section.

(3 Some of the data provided to Reviewer 3 concerning the specificity of the assay (Fig. for reviewers 3, 5-7).

Following the reviewer's comment, we have now added figure '3 for the reviewers' as EV Figure 4 in the main manuscript. And figures '6-7 for the reviewers' as a Figure EV 2 in the main manuscript. We also added source data for these figures.

Minor points:
(4Although important, Expanded View figure 1A (PCA analysis) is not mentioned anywhere in the manuscript. The labels on the figure are also small and hard to read.

We have improved the labels and mentioned this result.

(5Figure 1B should show error bars if n=3.

We thank the reviewer for this comment and have now added error bars

----
Referee #2:

The authors extensively responded to the critiques.

Referee #3:

The authors sufficiently addressed all point raised in my initial review. The improved manuscript provides interesting experimental evidence for the formation of mitochondrial derived vesicles (MDVs) in vitro.

2nd Revision - Editorial Decision 15th Feb 2023

Dear Dr. Regev-Rudzki

Thank you for the submission of your revised manuscript to our editorial offices. I now went through this and your p-b-p-response, and I consider the remaining referee concerns as adequately addressed.

However, I have these further editorial requests:

- We now request to show single datapoints for all diagrams with n<5. Please do that for all the diagrams shown. I.e. in case n=3, the three data points should be shown together with the mean/medium and the error bars.

- Please provide full statistics for the data shown in the diagrams in Fig. 1B, 5A, EV2B, EV2D, EV4 and EV5C. In all diagrams please also indicate (e.g. with n.s.) if testing was performed, but the differences are not significant.

- There is EV figure source data labelled as being data for Fig. EV6. Please check and rename this correctly.

I look forward to seeing the final revised version of your manuscript when it is ready. Please let me know if you have questions regarding the revision.

Please use this link to submit your revision: https://embor.msubmit.net/cgi-bin/main.plex

Best,

Achim Breiling
Senior Editor
EMBO Reports

The authors have addressed all minor editorial requests.

Dr. Neta Regev-Rudzki
Weizmann Institute of Science
Biomolecular Sciences
Herzel 243
Rehovot 7610001
Israel

Dear Dr. Regev-Rudzki,

I am very pleased to accept your manuscript for publication in the next available issue of EMBO reports. Thank you for your contribution to our journal.

At the end of this email I include important information about how to proceed. Please ensure that you take the time to read the information and complete and return the necessary forms to allow us to publish your manuscript as quickly as possible.

As part of the EMBO publication's Transparent Editorial Process, EMBO reports publishes online a Review Process File to accompany accepted manuscripts. As you are aware, this File will be published in conjunction with your paper and will include the referee reports, your point-by-point response and all pertinent correspondence relating to the manuscript.

If you do NOT want this File to be published, please inform the editorial office within 2 days, if you have not done so already, otherwise the File will be published by default [contact: emboreports@embo.org]. If you do opt out, the Review Process File link will point to the following statement: "No Review Process File is available with this article, as the authors have chosen not to make the review process public in this case." Please note that the author checklist will still be published even if you opt out of the transparent process.

Thank you again for your contribution to EMBO reports and congratulations on a successful publication. Please consider us again in the future for your most exciting work.

Yours sincerely,

Achim Breiling
Editor
EMBO Reports

****

THINGS TO DO NOW:

Once your article has been received by Wiley for production, the corresponding author will receive an email from Wiley's Author Services system which will ask them to log in and will present them with the appropriate license for completion.

You will receive proofs by e-mail approximately 2-3 weeks after all relevant files have been sent to our Production Office; you should return your corrections within 2 days of receiving the proofs.

Please inform us if there is likely to be any difficulty in reaching you at the above address at that time. Failure to meet our deadlines may result in a delay of publication, or publication without your corrections.

All further communications concerning your paper should quote reference number EMBOR-2022-56114V4 and be addressed to emboreports@wiley.com.

Should you be planning a Press Release on your article, please get in contact with emboreports@wiley.com as early as possible, in order to coordinate publication and release dates.

EMBO Press Author Checklist

Corresponding Author Name: Neta-Regev Rudzki	
Journal Submitted to: EMBO Reports	
Manuscript Number: 56114V2	

USEFUL LINKS FOR COMPLETING THIS FORM
The EMBO Journal - Author Guidelines
EMBO Reports - Author Guidelines
Molecular Systems Biology - Author Guidelines
EMBO Molecular Medicine - Author Guidelines

Reporting Checklist for Life Science Articles (updated January 2022)
This checklist is adapted from Materials Design Analysis Reporting (MDAR) Checklist for Authors. MDAR establishes a minimum set of requirements in transparent reporting in the life sciences (see Statement of Task: 10.31222/osf.io/9sm4x). Please follow the journal's guidelines in preparing your manuscript.
Please note that a copy of this checklist will be published alongside your article.

Abridged guidelines for figures
1. Data
The data shown in figures should satisfy the following conditions:
- the data were obtained and processed according to the field's best practice and are presented to reflect the results of the experiments in an accurate and unbiased manner.
- ideally, figure panels should include only measurements that are directly comparable to each other and obtained with the same assay.
- plots include clearly labeled error bars for independent experiments and sample sizes. Unless justified, error bars should not be shown for technical replicates.
- if n<5, the individual data points from each experiment should be plotted. Any statistical test employed should be justified.
- Source Data should be included to report the data underlying figures according to the guidelines set out in the authorship guidelines on Data Presentation.

2. Captions
Each figure caption should contain the following information, for each panel where they are relevant:
- a specification of the experimental system investigated (eg cell line, species name).
- the assay(s) and method(s) used to carry out the reported observations and measurements.
- an explicit mention of the biological and chemical entity(ies) that are being measured.
- an explicit mention of the biological and chemical entity(ies) that are altered/varied/perturbed in a controlled manner.
- the exact sample size (n) for each experimental group/condition, given as a number, not a range;
- a description of the sample collection allowing the reader to understand whether the samples represent technical or biological replicates (including how many animals, litters, cultures, etc.).
- a statement of how many times the experiment shown was independently replicated in the laboratory.
- definitions of statistical methods and measures:
  - common tests, such as t-test (please specify whether paired vs. unpaired), simple χ2 tests, Wilcoxon and Mann-Whitney tests, can be unambiguously identified by name only, but more complex techniques should be described in the methods section;
  - are tests one-sided or two-sided?
  - are there adjustments for multiple comparisons?
  - exact statistical test results, e.g., P values = x but not P values < x;
  - definition of 'center values' as median or average;
  - definition of error bars as s.d. or s.e.m.

Please complete ALL of the questions below.
Select "Not Applicable" only when the requested information is not relevant for your study.

Materials

Newly Created Materials	Information included in the manuscript?	In which section is the information available? (Reagents and Tools Table, Materials and Methods, Figures, Data Availability Section)
New materials and reagents need to be available; do any restrictions apply?	Yes	Materials and Methods

Antibodies	Information included in the manuscript?	In which section is the information available? (Reagents and Tools Table, Materials and Methods, Figures, Data Availability Section)
For **antibodies** provide the following information:<br>- Commercial antibodies: RRID (if possible) or supplier name, catalogue number and or/clone number<br>- Non-commercial: RRID or citation	Yes	Materials and Methods

DNA and RNA sequences	Information included in the manuscript?	In which section is the information available? (Reagents and Tools Table, Materials and Methods, Figures, Data Availability Section)
**Short novel DNA or RNA including primers, probes**: provide the sequences.	Not Applicable	

Cell materials	Information included in the manuscript?	In which section is the information available? (Reagents and Tools Table, Materials and Methods, Figures, Data Availability Section)
**Cell lines:** Provide species information, strain. Provide accession number in repository **OR** supplier name, catalog number, clone number, and/**OR** RRID.	Yes	Materials and Methods
**Primary cultures:** Provide species, strain, sex of origin, genetic modification status.	Not Applicable	
Report if the cell lines were recently **authenticated** (e.g., by STR profiling) and tested for mycoplasma contamination.	Not Applicable	

Experimental animals	Information included in the manuscript?	In which section is the information available? (Reagents and Tools Table, Materials and Methods, Figures, Data Availability Section)
**Laboratory animals or Model organisms:** Provide species, strain, sex, age, genetic modification status. Provide accession number in repository **OR** supplier name, catalog number, clone number, **OR** RRID.	Yes	Materials and Methods
**Animal observed in or captured from the field:** Provide species, sex, and age where possible.	Not Applicable	
Please detail **housing and husbandry conditions**.	Not Applicable	

Plants and microbes	Information included in the manuscript?	In which section is the information available? (Reagents and Tools Table, Materials and Methods, Figures, Data Availability Section)
**Plants:** provide species and strain, ecotype and cultivar where relevant, unique accession number if available, and source (including location for collected wild specimens).	Not Applicable	
**Microbes:** provide species and strain, unique accession number if available, and source.	Not Applicable	

Human research participants	Information included in the manuscript?	In which section is the information available? (Reagents and Tools Table, Materials and Methods, Figures, Data Availability Section)
If collected and within the bounds of privacy constraints report on age, sex and gender or ethnicity for all study participants.	Not Applicable	

Core facilities	Information included in the manuscript?	In which section is the information available? (Reagents and Tools Table, Materials and Methods, Figures, Data Availability Section)
If your work benefited from core facilities, was their service mentioned in the acknowledgments section?	Yes	Acknowledgements

Design

Study protocol	Information included in the manuscript?	In which section is the information available? (Reagents and Tools Table, Materials and Methods, Figures, Data Availability Section)
If study protocol has been **pre-registered, provide DOI in the manuscript**. For clinical trials, provide the trial registration number **OR** cite DOI.	Not Applicable	
Report the **clinical trial registration number** (at ClinicalTrials.gov or equivalent), where applicable.	Not Applicable	

Laboratory protocol	Information included in the manuscript?	In which section is the information available? (Reagents and Tools Table, Materials and Methods, Figures, Data Availability Section)
Provide DOI OR other citation details if **external detailed step-by-step protocols** are available.	Yes	Materials and Methods

Experimental study design and statistics	Information included in the manuscript?	In which section is the information available? (Reagents and Tools Table, Materials and Methods, Figures, Data Availability Section)
Include a statement about **sample size** estimate even if no statistical methods were used.	Yes	Figure legends
Were any steps taken to minimize the effects of subjective bias when allocating animals/samples to treatment (e.g. **randomization procedure**)? If yes, have they been described?	Not Applicable	
Include a statement about **blinding** even if no blinding was done.	Not Applicable	
Describe **inclusion/exclusion criteria** if samples or animals were excluded from the analysis. Were the criteria pre-established?<br><br>If sample or data points were omitted from analysis, report if this was due to attrition or intentional exclusion and provide justification.	Not Applicable	
For every figure, are **statistical tests** justified as appropriate? Do the data meet the assumptions of the tests (e.g., normal distribution)? Describe any methods used to assess it. Is there an estimate of variation within each group of data? Is the variance similar between the groups that are being statistically compared?	Yes	Figure legends

Sample definition and in-laboratory replication	Information included in the manuscript?	In which section is the information available? (Reagents and Tools Table, Materials and Methods, Figures, Data Availability Section)
In the figure legends: state number of times the experiment was **replicated** in laboratory.	Yes	Figure legends
In the figure legends: define whether data describe **technical or biological replicates**.	Yes	Figure legends

Ethics

Ethics	Information included in the manuscript?	In which section is the information available? (Reagents and Tools Table, Materials and Methods, Figures, Data Availability Section)
Studies involving **human participants**: State details of **authority granting ethics approval** (IRB or equivalent committee(s), provide reference number for approval.	Not Applicable	
Studies involving **human participants**: Include a statement confirming that **informed consent** was obtained from all subjects and that the experiments conformed to the principles set out in the WMA Declaration of Helsinki and the Department of Health and Human Services Belmont Report.	Not Applicable	
Studies involving **human participants:** For publication of **patient photos**, include a statement confirming that consent to publish was obtained.	Not Applicable	
Studies involving experimental **animals**: State details of **authority granting ethics approva**l (IRB or equivalent committee(s), provide reference number for approval. Include a statement of compliance with ethical regulations.	Not Applicable	
Studies involving **specimen and field samples:** State if relevant **permits** obtained, provide details of authority approving study; if none were required, explain why.	Not Applicable	

Dual Use Research of Concern (DURC)	Information included in the manuscript?	In which section is the information available? (Reagents and Tools Table, Materials and Methods, Figures, Data Availability Section)
Could your study fall under dual use research restrictions? Please check biosecurity documents and list of **select agents and toxins** (CDC): https://www.selectagents.gov/sat/list.htm	Not Applicable	
If you used a select agent, is the security level of the lab appropriate and reported in the manuscript?	Not Applicable	
If a study is subject to dual use research of concern regulations, is the name of the **authority granting approval and reference number** for the regulatory approval provided in the manuscript?	Not Applicable	

Reporting

The MDAR framework recommends adoption of discipline-specific guidelines, established and endorsed through community initiatives. Journals have their own policy about requiring specific guidelines and recommendations to complement MDAR.

Adherence to community standards	Information included in the manuscript?	In which section is the information available? (Reagents and Tools Table, Materials and Methods, Figures, Data Availability Section)
State if relevant guidelines or checklists (e.g., **ICMJE, MIBBI, ARRIVE, PRISMA**) have been followed or provided.	Not Applicable	
For **tumor marker prognostic studies,** we recommend that you follow the **REMARK** reporting guidelines (see link list at top right). See author guidelines, under 'Reporting Guidelines'. Please confirm you have followed these guidelines.	Not Applicable	
For **phase II and III randomized controlled trials**, please refer to the **CONSORT** flow diagram (see link list at top right) and submit the CONSORT checklist (see link list at top right) with your submission. See author guidelines, under 'Reporting Guidelines'. Please confirm you have submitted this list.	Not Applicable	

Data Availability

Data availability	Information included in the manuscript?	In which section is the information available? (Reagents and Tools Table, Materials and Methods, Figures, Data Availability Section)
Have **primary datasets** been deposited according to the journal's guidelines (see 'Data Deposition' section) and the respective accession numbers provided in the Data Availability Section?	Yes	Data Availability Section
Were **human clinical and genomic datasets** deposited in a public access-controlled repository in accordance to ethical obligations to the patients and to the applicable consent agreement?	Not Applicable	
Are **computational models** that are central and integral to a study available without restrictions in a machine-readable form? Were the relevant accession numbers or links provided?	Not Applicable	
If publicly available data were reused, provide the respective **data citations in the reference list**.	Not Applicable	
